# Polyphenolic glycosides isolated from *Pogostemon cablin* (Blanco) Benth. as novel influenza neuraminidase inhibitors

**DOI:** 10.1186/s13065-016-0192-x

**Published:** 2016-08-10

**Authors:** Fang Liu, Wei Cao, Chao Deng, Zhaoquan Wu, Guangyao Zeng, Yingjun Zhou

**Affiliations:** College of Pharmacy, Central South University, Changsha, 410013 Hunan People’s Republic of China

**Keywords:** Octaketide, Polyphenolic glycosides, *Pogostemon cablin* (Blanco) Benth., Neuraminidase (NA) inhibitory activity

## Abstract

**Background:**

Influenza is historically an ancient disease that causes annual epidemics and, at irregular intervals, pandemics. At present, the first-line drugs (oseltamivir and zanamivir) don’t seem to be optimistic due to the spontaneously arising and spreading of oseltamivir resistance among influenza virus. *Pogostemon cablin* (Blanco) Benth. (*P. cablin*) is an important traditional Chinese medicine herb that has been widely used for treatment on common cold, nausea and fever. In our previous study, we have identified an extract derived from *P. cablin* as a novel selective neuraminidase (NA) inhibitor.

**Results:**

A series of polyphenolic compounds were isolated from *P. cablin* for their potential ability to inhibit neuraminidase of influenza A virus. Two new octaketides (**1**, **2**), together with other twenty compounds were isolated from *P. cablin*. These compounds showed better inhibitory activity against NA. The significant potent compounds of this series were compounds **2** (IC_50_ = 3.87 ± 0.19 μ mol/ml), **11**, **12**, **14**, **15**, **19** and **20** (IC_50_ was in 2.12 to 3.87 μ mol/ml), which were about fourfold to doubled less potent than zanamivir and could be used to design novel influenza NA inhibitors, especially compound **2**, that exhibit increased activity based on these compounds. With the help of molecular docking, we had a preliminary understanding of the mechanism of the two new compounds (**1–2**)’ NA inhibitory activity.

**Conclusions:**

Fractions 6 and polyphenolic compounds isolated from fractions 6 showed higher NA inhibition than that of the initial plant exacts. The findings of this study indicate that polyphenolic compounds and fractions 6 derived from *P. cablin* are potential NA inhibitors. This work is one of the evidence that *P. cablin* has better inhibitory activity against influenza, which not only enriches the compound library of *P. cablin*, but also facilitates further development and promises its therapeutic potential for the rising challenge of influenza diseases.

**Electronic supplementary material:**

The online version of this article (doi:10.1186/s13065-016-0192-x) contains supplementary material, which is available to authorized users.

## Background

Influenza can cause serious public health and economic problems, which affects millions of people worldwide. Despite advances in the understanding of molecular and cellular aspects of influenza, the disease remains the major cause of mortality and morbidity among patients with respiratory diseases [1].

Influenza viruses have several proteins that are implicated in virulence: the surface proteins hemagglutinin (HA) and neuraminidase (NA), the polymerase complex (including the PB1, PB2 and PA proteins), and the non-structural proteins [2]. NA is an antiviral target of high pharmaceutical interest because of its essential role in cleaving sialic acid residues from cell surface glycoprotein and facilitating release of virions from infected cells.

The anti-influenza drugs approved for clinical use are the NA inhibitors (orally administered oseltamivir trade name Tamiflu and inhaled zanamivir trade name Relenza). Both of them are sialic acid (Neu5Ac) analogues. Because such inhibitors may be structurally recognized as inhibitors by the cellular NA from the host, this might result in side effects. Therefore, developing novel NA inhibitors to combat influenza virus is desirable.

Natural products, especially those derived from traditional Chinese medicine herbs (TCMH), are still the major source of innovative therapeutic agents for infectious diseases, cancer, lipid disorders and immunomodulation [3]. *Pogostemon cablin* is an annual herb mostly distributed in the tropical and subtropical regions of Asia. *P. cablin* has been recorded in Chinese Pharmacopoeia as a traditional herbal medicine for its therapeutic functions, including eliminating heat and dampness, calming nerves, and alleviating fatigue. It is used in traditional Chinese medicine for the treatment of upset stomach, vomiting and diarrhea, headache, and fever [4]. Chemical and pharmacological researches on *P. cablin* have been carried out in recent years [5]. A number of mono- and sesquiterpenoids [6], triterpenoids and steroids [7], flavonoids [8], alkaloids [9] and phenylpropanoid glycosides [10] have been discovered from the title plant.

*P. cablin* and polyphenolic compounds present in them have gained a lot of interest due to their beneficial health implications. Dietary polyphenolic compounds, especially phenylpropanoid glycosides, exert antioxidant properties and are better inhibitors of NA of influenza A virus [11]. In our ongoing effort to characterize new natural compounds used in Traditional Chinese Medicine (TCM) herbs with interesting chemical structures and/or pharmaceutical activities, we studied on the chemical constituents of the aerial parts of *P. cablin*, which led to the isolation of two new octaketides (**1**, **2**), together with other twenty compounds were isolated from *P. cablin*. This is the first report that presents compounds **1–9**, **11** and **21–22** in this genus.

**Fig. 1:**
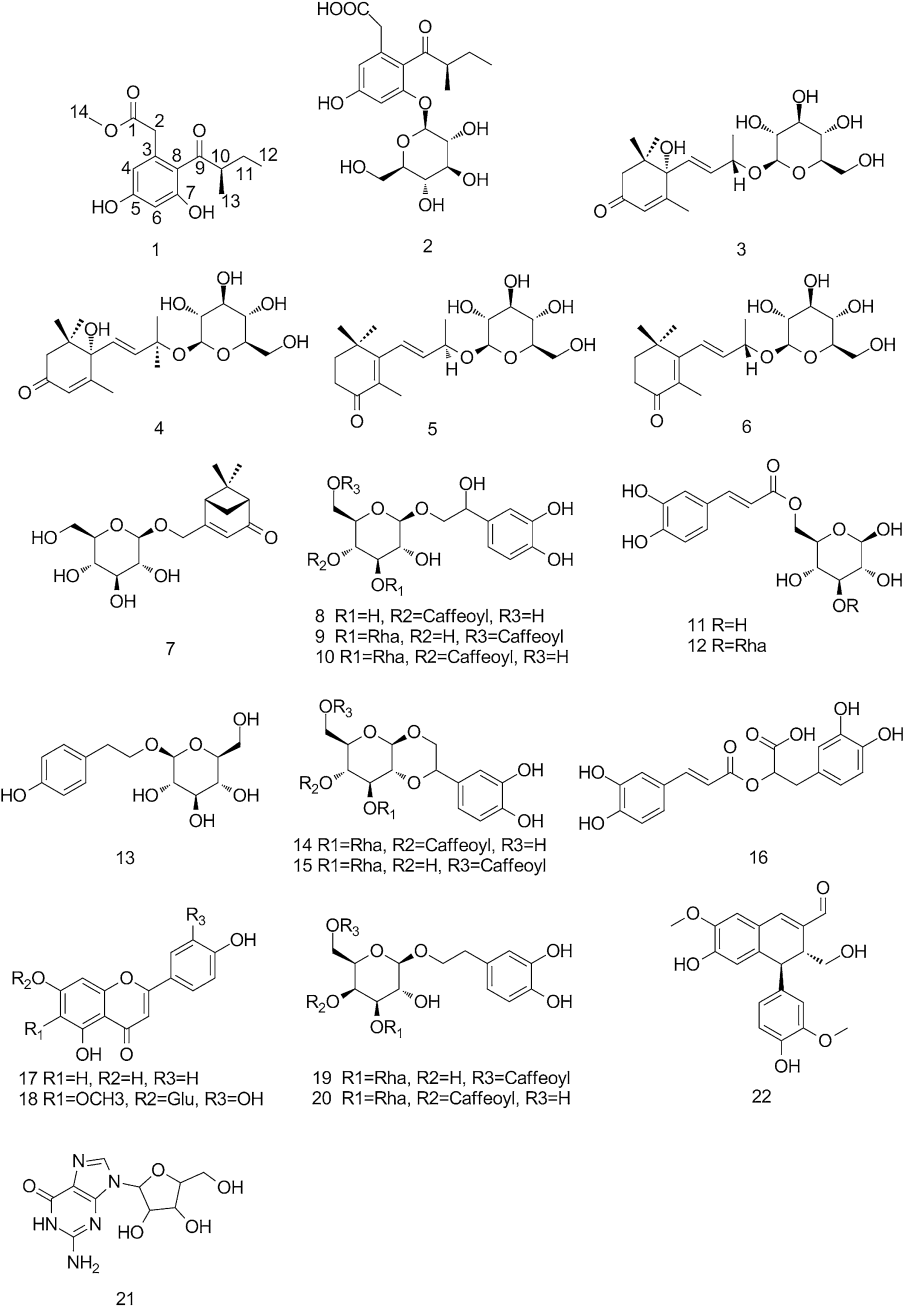
Chemical structures of compound **1–22**

In a previous study from our research group, several extracts derived from *P. Cablin* have better inhibitory activity on NA. In extending these studies, we examined the effects of these compounds against NA activity. According to the results obtained, the extracts exhibited better inhibitory activity against NA, and the polyphenolic compounds presents in them are responsible for their biological properties. Our current results imply that these specific plant extracts are a possible source of new natural NA inhibitors (Fig. 1).

## Results and discussion

### Structures elucidation of compounds

**Compound 1:**

**Fig. 2:**
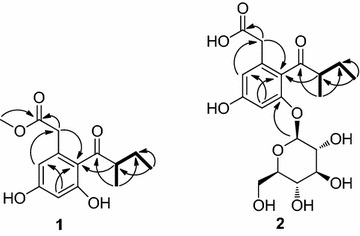
Key HMBCs (→) and ^1^H-^1^H COSY (*bold*) correlations of compound **1**–**2**

 Named cytosporone VI, colorless noodle-like crystal with a negative optical rotation ([*α*]_*D*_^15^ − 9.5, c = 0.5, CHCl_3_). The molecular formula of compound **1** was determined as C_14_H_18_O_5_ from its positive mode HR-ESI MS data at m/z 289.1051 [M + Na]^+^ (calcd for C_14_H_18_O_5_Na, 289.1052), which was compatible with ^1^H NMR and ^13^C NMR data. The ^1^H NMR and ^13^C NMR spectral data (Table 1) of compound **1**, in combination with HSQC, indicated the co-existence in the molecule of two metacoupled aromatic methines: δ_H_ 6.29 (1H, d, *J* = 4 Hz), δ_C_ 101.42 and δ_H_ 6.25 (1H, d, *J* = 4 Hz), δ_C_ 110.43; one carboxyl group: δ_C_ 172.75; and a methylene: δ_H_ 3.50 (2H), δ_C_ 38.94, which is presumably located between the phenyl and carboxyl groups. Furthermore, a side chain was indicated by one ketone group: δ _C_ 211.28; two methyl groups: δ_H_ 0.90 (3H, t), δ_C_ 10.91 and δ_H_ 1.08 (3H, d, *J* = 8.5 Hz), δ_C_ 14.87, a methylene: δ_H_ 1.35 (1H, m), 1.75 (1H, m), δ_C_ 25.94, and a methine: δ_H_ 3.40 (1H, m), δ_C_ 47.23. The side chain was determined to be 2- methylbutan-1-one by the ^1^H-^1^H COSY and TOCSY spectra, revealing the ^1^H -^1^ H spin systems of H-10/H-11/H-12 and H-10/H-13, and the HMBC spectra correlations of H-12/C-10, H-12/C-11, H-13/C-9 and H-13/C-11 (Fig. 2). In conjunction with other key HMBC correlations of H-2/C-1, H-2/C-8, H-4/C-2, H-6/C-4, H-6/C-8, and H-10/C-8, these observations suggested that compound **1** was assigned as a 5, 7-dihydroxy-8-(2-methylbutan-1-onyl)-ethyl phenylmethyl ester. This is structurally associated with cytosporone analogues. The absolute configuration of C-10 in the side chain was established as R by comparing the specific rotation value ([*α*]_*D*_^15^ − 9.5, c = 0.5, CHCl_3_) for 1 to those known synthetic isomeric compounds, which showed a negative specific rotation for the R-configuration and a positive specific rotation for the S-configuration in the side chain of the related synthetic ones ((2R)-l-phenyl-2-methylbutan-l-one, [*α*]_*D*_^15^− 36.9 and (2S)-l-phenyl-2-methylbutan-l-one, [*α*]_*D*_^15^ + 36.8) [12]. On the basis of above data, the structure of **1** was elucidated as 5, 7-dihydroxy-8-((2R)-2-methylbutan-1-onyl)-methyl phenylacetate.

**Table 1:** ^1^H (500 MHz) and ^13^C (125 MHz) NMR spectral data of compounds **1** and **2**

Position	1^a^		2^b^	
	δ_C_	δ_H_	δ_C_	δ_H_
1	172.75		172.08	
2	38.94	3.50	38.77	3.33
3	135.79		135.80	
4	119.83		122.53	
5	158.30		156.88	
6	101.42	6.29, d (4)	100.86	6.49, br s
7	159.95		159.59	
8	110.43	6.25, d (4)	112.28	6.37, br s
9	211.28		209.76	
10	47.23	3.40, m	47.44	3.25
11	25.94	1.35, m 1.75, m	25.75	1.24, m 1.65, m
12	10.91	0.90, t	11.90	0.82, t
13	14.87	1.08, d (8.5)	15.76	0.98, d (6.5)
14-OCH3	51.02	3.68, s		
Glu-1			100.25	4.93, d (7.5)
Glu-2			77.54	3.31
Glu-3			77.45	3.31
Glu-4			69.94	3.16
Glu-5			73.82	3.19
Glu-6			61.00	3.70 3.49

^a^Measured in CD_3_OD

^b^Measured in DMSO-*d*_6_, *δ* Chemical shifts are given in ppm, *J* values are in parentheses and reported in Hz

**Compound 2:**

**Fig. 3:**
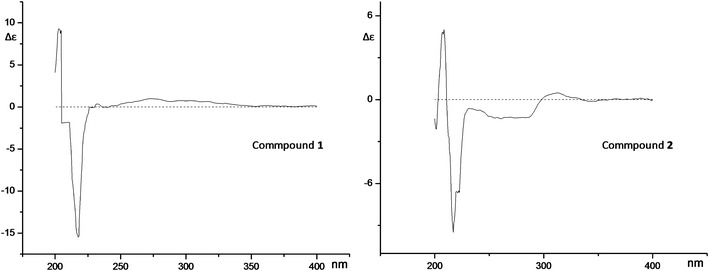
The CD curves of compounds **1** and **2**

 White amorphous powder (MeOH), the molecular formula of compound **2** was determined to be C_19_H_26_O_10_ on the basis of HR-ESI MS (m/z 437.1390 [M + Na]^+^, calcd for C_19_H_26_O_10_Na, 437.1424) in the positive mode HR-ESI MS. For the ^1^ H NMR and ^13^ C NMR spectral data of compound **2** see Table 1. The aglucone of compound **2** was an analogue compound of **1**, and the HMBC spectra correlation between H-1′ and C-7 confirmed the position of glucopyranosyl moiety. The absolute configuration of C-10 in the side chain was established as R for the CD spectra of **2** (217 nm, Δε −9.49; 208 nm, Δε +5.01) which in accordance with compound **1** (218 nm, Δε −15.47; 205 nm, Δε +9.12) (Fig. 3). On the basis of above data, the structure of **2** was elucidated as 5, 7-dihydroxy-8-((2R)-2-methylbutan-1-onyl)-phenylacetic acid 7-O-β-D-glucopyranoside.

**Fig. 4:**
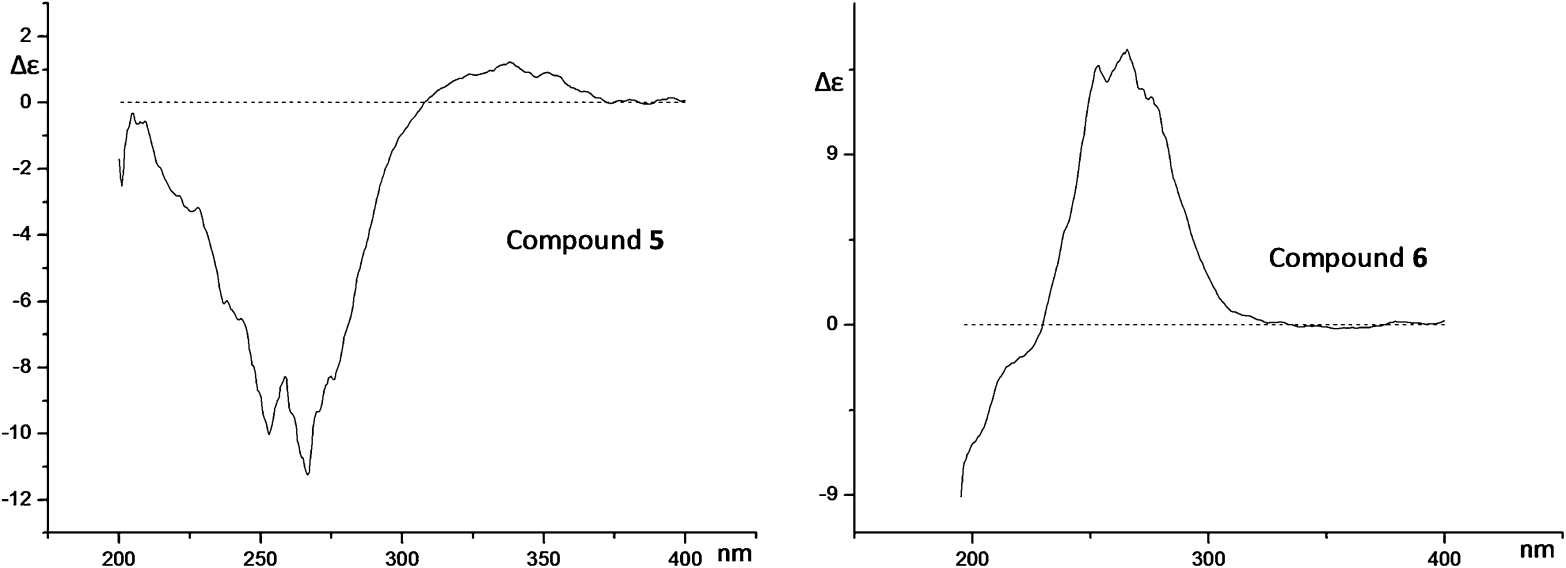
The CD curves of compounds **5** and **6**

The structures of compound (7 E, 9 S)-9-Hydroxy-5, 7-megastigmadien-4-one 9-O-β-D-glucopyranoside (**5**) and (7 E, 9 R)-9-Hydroxy-5, 7-megastigmadien-4-one 9-O-β-D-glucopyranoside (**6**) [13], which were isolated from *P. cablin* for the first time, were deduced by analyzing the MS, 1D, 2D NMR spectra, rotation and CD curves (Fig. 4). The CD curves of compounds **5** and **6** were also firstly reported in this report.

The compounds **3**, **4** and **7–22** were identified by comparison of their physicochemical data (NMR, MS, [α]) with those reported in the literature as (6 S, 7 E, 9 S)-6, 9-Dihydroxy-4, 7-megastigmadien-3-one 9- O-β-D-glucopyranoside (**3**) [14], (6 S, 7 Z, 9 R)-6, 9-Dihydroxy-4, 7-megastigmadien -3-one 9- O-β-D-glucopyranoside (**4**) [15], and Vervenone- 10-O-β-D-glucopyranoside (**7**) [16], 2- (3, 4-dihydroxyphenyl)-2-hydroxyethyl, 4- [(2E)-3- (3, 4-dihydroxyphenyl)-2-propenoate] β- D- Glucopyranoside (**8**) [17], isocampneoside II (**9**), campneoside II (**10**), 4- [(2E)-3- (3, 4-dihydroxyphenyl)-2-propenoate β- D- Glucopyranoside (**11**), cistanoside F (**12**), descaffeoyl crenatoside (**13**) [18, 19], crenatoside (**14**), isocrenatoside (**15**) [20], rosmarinic acid (**16**), apigenin (**17**) [21], nepetin (**18**), [22] isopedicularioside G (**19**), pedicularioside G (**20**) [23], guanosine (**21**) [24], 6-Hydroxy-4-(4-hydroxy-3-methoxyphenyl)-3-hydroxymethyl-7-methoxy-3, 4-dihydro-2-naphthaldehyde (**22**) [25], respectively (Additional file 1). The compounds **1–9**, **11, 18, 19** and **21–22** were isolated from *P. cablin* for the first time.

### Evaluation of NA inhibition activity

**Fig. 5:**
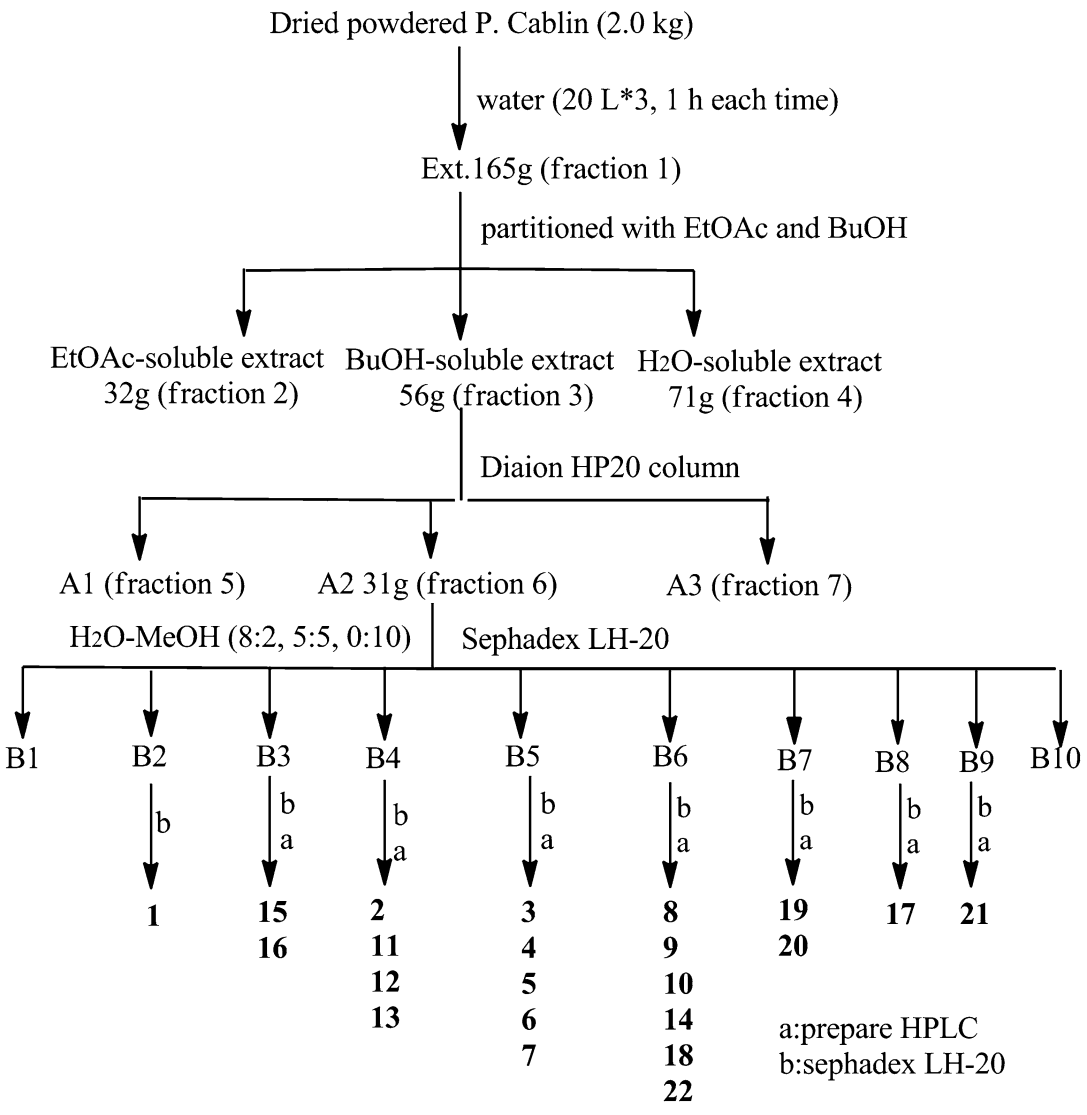
Process for the separation of compounds **1–22**

NA remains an attractive anti-influenza drug target, while the emergence of viruses resistant to currently available drugs has presented a new challenge. Therefore, compounds **1–22** and fractions 1–7 (Fig. 5) were tested for their inhibitory effects against the influenza virus NA in vitro with the commercial NA inhibitory screening kit. Even though a number of biological activity studies on this plant have been performed, so far only a few anti-influenza virus constituents from *P. cablin* have been reported. In this study, the half inhibitory concentration (IC_50_) of compounds **1–22** were evaluated for their inhibitory effects against the influenza virus NA in vitro as a screening system. The NA inhibitory activity experiment results are shown in Tables 2 and 3 (Additional file 2).

**Table 2:** NA inhibition activity of compounds **1–22**

Compound	IC_50_ (μ mol/ml)	Compound	IC_50_ (μ mol/ml)	Compound	IC_50_ (μ mol/ml)
1	8.40 ± 1.20	9	6.08 ± 0.20	17	4.69 ± 0.29
2	3.87 ± 0.19	10	6.53 ± 0.38	18	3.29 ± 0.04
3	11.62 ± 0.48	11	3.60 ± 0.02	19	2.74 ± 0.03
4	10.99 ± 1.15	12	2.99 ± 0.12	20	2.12 ± 0.04
5	10.93 ± 0.48	13	7.87 ± 0.13	21	32.67 ± 4.73
6	19.94 ± 1.95	14	3.30 ± 0.12	22	4.70 ± 0.05
7	>200	15	3.64 ± 0.17		
8	6.32 ± 0.38	16	2.27 ± 0.09	Zanamivir	0.93 ± 0.02

Zanamivir was the positive control; each value represents the mean ± SD (n = 3)

**Table 3:** NA inhibition activity of fraction **1**–**7**

Fractions	Inhibition rate % (1 mg/ml, DMSO)
1	44.71 ± 1.53
2	35.71 ± 1.15
3	69.70 ± 1.16
4	20.05 ± 1.00
5	26.38 ± 0.58
6	90.69 ± 1.53
7	18.72 ± 0.58

Each value represents the mean ± SD (n = 3)

Good oral availability can be achieved by right balance between partitioning and solubility properties. To understand the properties of the proposed compounds better, we utilized *Molinspiration* [26] to predict some properties of the typical compounds (**1**, **2**, **16**, **20** and **22**) (Table 4), and applied the Lipinski’s rule of five [27] to see whether all passed the criteria. Lipinski’s rule of five acts as a filter for drug like properties and states that a potential molecule is orally active if it’s molecular weight is ⩽500 da, log P ⩽5, number of hydrogen bond acceptors ⩽10, number of hydrogen bond donors ⩽5. Under the Lipinski’s rule of five, compounds (**1**, **2**, **16**, **20** and **22**) presenting mi log P (< 5) suggested that they may all have good oral bioavailability, and compounds **1** and **22** might be two lead compounds for anti-influenza. (mi log P: logarithm of compound partition coefficient between n-octanol and water).

**Table 4:** Theoretical prediction of properties of compounds **1**, **2**, **16**, **20** and **22**

Compound	mi log P	TPSA	MW	nON	nOHNH	nviolations	Volume	nrotb
1	2.49	83.83	266.29	5	2	0	247.15	6
2	−0.38	173.98	414.41	10	6	1	361.74	8
16	1.63	144.52	360.32	8	5	0	303.54	7
20	−0.45	245.29	624.59	15	9	3	532.50	11
22	2.01	96.22	356.37	6	3	0	316.61	5

*mi log P* logarithm of compound partition coefficient between n-octanol and water; *TPSA* topological polar surface area; *MW* molecular weight; *nON* number of hydrogen bond acceptors; *nOHNH* number of hydrogen bond donors; *Nrotb* number of rotatable bonds

### Molecular docking studies

Earlier crystallographic and ensuing SAR studies have revealed that the active site of NA could be divided into four major binding sites [28]. All NA inhibitors on the market or in clinical phases possess strong structural resemblance in those parts, which correspond to the fact that the four pockets are critical for interaction with the active site of NA.

The pocket C1 is comprised of positively charged guanidino groups of arginines 118, 292 and 371 and interacts with the carboxylate. In pocket C5, Arg 152 functions as the hydrogen-bond donor. Trp 178 and Ile 222 comprise a small hydrophobic region. In pocket C4, usually a guanidine or an amine group participates in charge–charge interactions and hydrogen bonds to Glu 119, Asp 151, and/or Glu 227. In pocket C6, Glu 276, the side chain of Arg 152, the amidic carbonyl of Trp 178 and Asp 151 form a new hydrophobic binding pocket. Moreover, Glu 277 and Tyr 406 are believed to play a critical role in the catalytic activity of NA [29, 30].

**Fig. 6:**
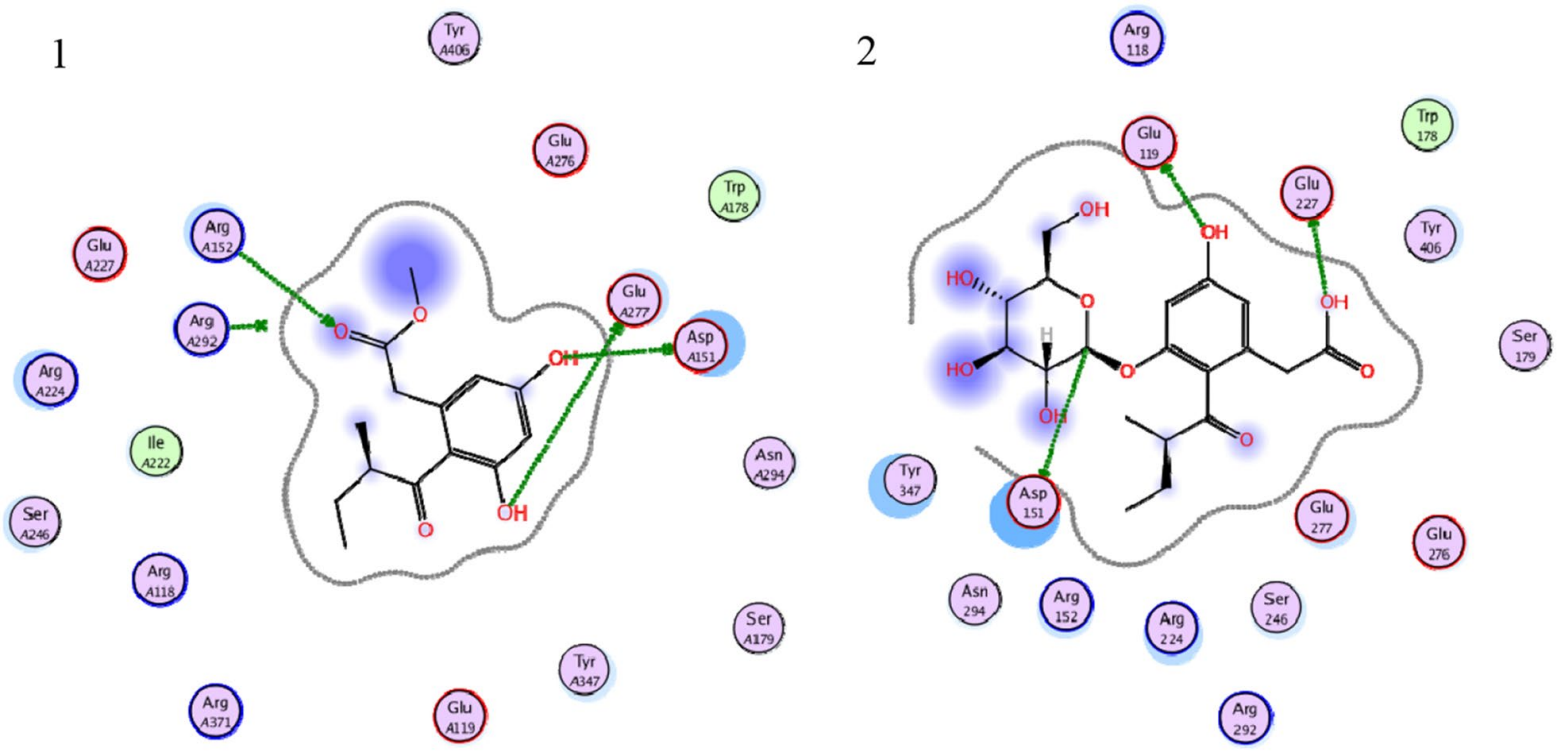
Molecular models of compounds **1** and **2** binding to active site of NA

From the activity assay results, compounds **1** and **2** showed better inhibitory activities against NA. To provide a further insight on the observed activities, the binding of compounds **1** and **2** in the active site of NA is shown in Fig. 6. we find that the-COOH group of compound **2** interacts with the pocket C4 of NA active site by hydrogen bond with Glu 119 of this subsite, anomeric carbon of glucose binds to the pocket C4 by hydrogen bond interaction with Asp 151, and 5-OH group forms hydrogen bond with Glu 227 of pocket C4.

**Fig. 7:**
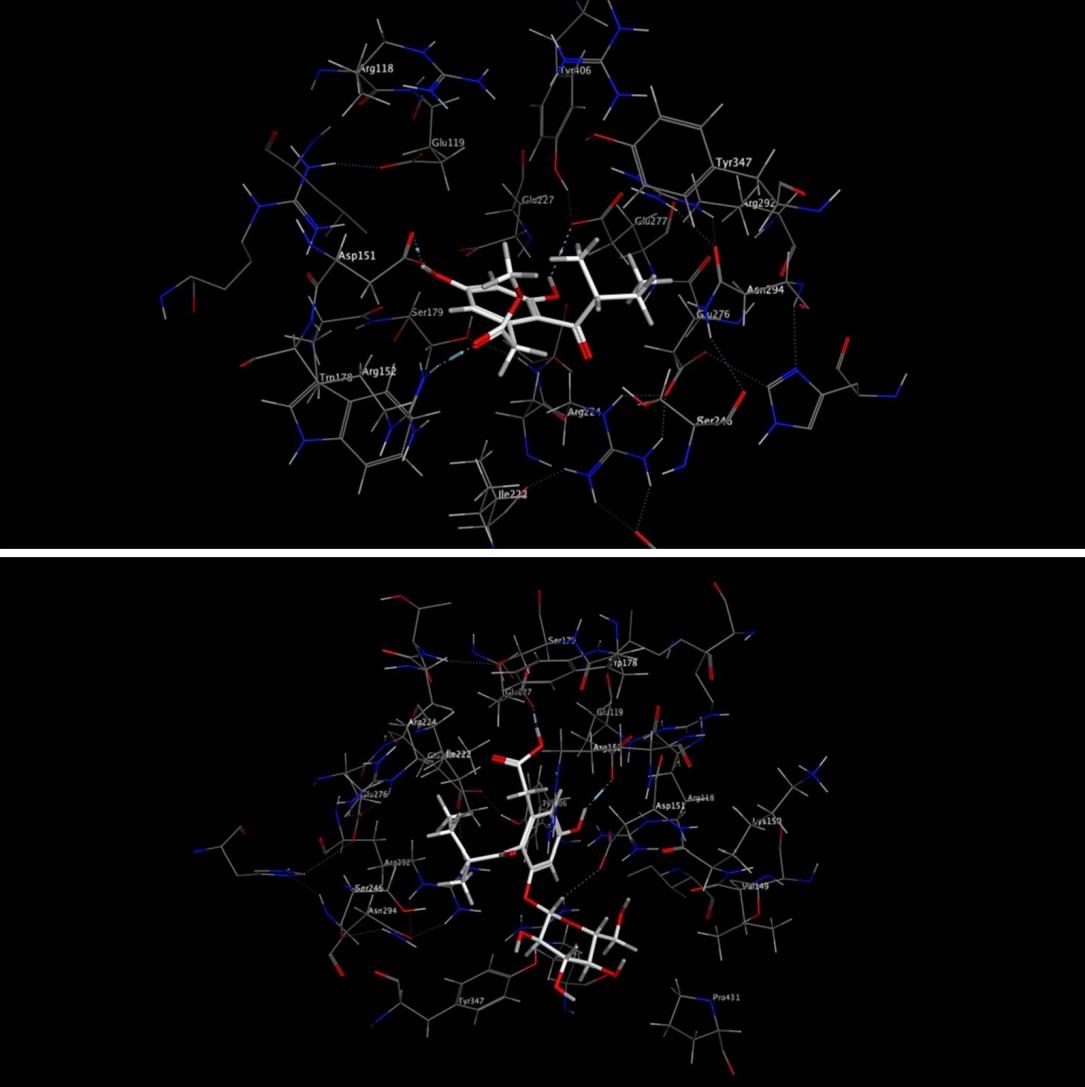
Detailed view of the docking results of compounds **1** and **2** in the active site of neuraminidase (PDB ID: 2HU4). The *Sky blue lines* and *numbers* show the potential hydrogen bonds and bond length. The first one is compound **1**, and the second one is compound **2**

Moreover, for compound **1**, the 7-OH group binds to the pocket C6 by hydrogen bond interaction with Glu 277, the 1-CO-group forms a hydrogen bond with Arg 152 and Arg 292 of pocket C1, and the 5-OH group binds to the pocket C4 by hydrogen bond interaction with Asp151 (Fig. 7).

The binding of compound **1** in the active site of NA showed that the three pockets (C1, C4, C6) of the active site of NA were occupied, although not so well as zanamivir, but still can be a lead compound.

## Methods

### General information

Optical rotations were recorded on a Jasco P-2000 automatic digital polarimeter. The ^1^ H NMR, ^13^C NMR, ^1^H-^1^H COSY, HSQC and HMBC spectra were recorded on a Bruker AM 500 spectrometer with TMS as the internal standard at 500 MHz and 125 MHz for ^1^ H and ^13^ C. The enzyme activity inhibition assay was carried out on a microplate spectrophotometer (Gemini EM; Molecular Devices). Circular dichroism (CD) spectra were recorded on a CD spectrometer (JASCO, J-815-150S, Japan). Optical rotations were recorded on an automatic digital polarimeter (Shenguang SGW-3, China). Preparative HPLC: Agilent 1100 Series HPLC system, a reverse-phase C18 column (YMC-Pack ODS-A, 250*20 mm, 5 μm, YMC Co., Ltd, Kyoto, Japan). Column chromatography was performed with Diaion HP20 (Mitsubishi, Japan) and Sephadex LH-20 (Pharmacia (GE)). TLC was carried out on precoated silica gel GF 254 plates (Qingdao Haiyang Chemical Co. Ltd), and spots were visualized under UV light (254 or 365 nm) or detected by spraying with 10 % H_2_SO_4_ in EtOH followed by heating.

### Plant material

The aerial part of *P. cablin* was purchased from Suixi county, Guangdong province, China, in September 2014. The botanical identification was made by Associate Prof. Jin-ping Li. A voucher specimen (NO.GHX140918) was deposited in College of Pharmacy, Central South University.

### Extraction and isolation

Dried powdered *P. Cablin* (2.0 kg) was extracted with water (20 L × 3, 1 h each time) by reflux. The extracts were then concentrated under vacuum to afford a crude extract (165 g), which was suspended in H_2_O and successively partitioned with EtOAc and BuOH, yielding 32 g of EtOAc—soluble extract, 56 g of BuOH-soluble extract and 71 g of H_2_O-soluble extract. BuOH—Soluble extract (56 g) was applied to a Diaion HP20 column (10 × 200 cm) with a step gradient elution of EtOH-H_2_O (v/v 0:1, 4:6, 9.5:0.5) to provide three factions: A1, A2 and A3. A2 (31 g) was chromatographed over a Sephadex LH-20 column (6 × 250 cm) eluted with H_2_O-MeOH system (8:2, 5:5, 0:10) to give B1–B10.

B2 (300 mg) was chromatographed on a Sephadex LH-20 column (2 × 150 cm) eluted with MeOH to yield B2-1, then B2-1 on a Sephadex LH-20 column (2 × 90 cm) CH_2_Cl_2_-MeOH system (8:2) to give compound **1** (11 mg, TLC: CH_2_Cl_2_-MeOH 10-0.1, Rf = 0.3).

B3 was chromatographed on a Sephadex LH-20 column (2 × 150 cm) eluted with MeOH system and then was purified by preparative reverse-phase HPLC eluted with 40 % MeOH/H_2_O (+0.2 % formic acid (FA)) to give compound **15** (7 mg, t_R_ = 23 min) and compound **16** (8 mg, t_R_ = 19 min).

B4 was chromatographed on a Sephadex LH-20 column (2 × 150 cm) eluted with MeOH system, and then five fractions (D1–D5) were got. D2 was on a Sephadex LH-20 column (2 × 150 cm) eluted with MeOH system to give two fractions D2-1 and D2-2, then D2-1 and D2-2 were chromatographed on a Sephadex LH-20 column (2 × 90 cm) eluted with CH_2_Cl_2_-MeOH system (8:2) to give compound **11** (8 mg) and compound **12** (9 mg). D3 eluted with MeOH was purified by a Sephadex LH-20 column (2 × 150 cm), and then to give three fractions: D3-1, D3-2 and D3-3. D3-1 was purified by a Sephadex LH-20 column (2 × 90 cm) eluted with CH_2_Cl_2_-MeOH system (1:1) and then was purified by preparative reverse-phase HPLC eluted with 15 % MeCN/H_2_O (+0.2 % FA) to give compound **13** (7 mg, t_R_ = 16.5 min). D3-3 was purified by a Sephadex LH-20 column (2 × 90 cm) eluted with MeOH and then eluted with CH_2_Cl_2_-MeOH system (1:1) and purified by a Sephadex LH-20 column (2 × 150 cm) to give compound **2** (21 mg, TLC: EtOAc-FA-H_2_O: 10-1-1, Rf = 0.4).

B5 (1.1 g) was chromatographed on a Sephadex LH-20 column (2 × 150 cm) eluted with CH_2_Cl_2-_MeOH system (5:5) to give C1–C8, C3 (107 mg) chromatographed on a Sephadex LH-20 column (2 × 90 cm) eluted with H_2_O_-_MeOH system (5:5) to yield three fractions: C3-1 (36 mg), C3-2 (26 mg), C3-3 (50 mg). C3-1 was subsequently purified by preparative reverse-phase HPLC eluted with 11 % MeCN/H_2_O (+0.2 % FA) to give compounds **3** (9 mg, t_R_ = 18.5 min), **4** (13 mg, t_R_ = 20.5 min), C3-3 was subsequently purified by preparative reverse-phase HPLC eluted with 14 % MeCN/H_2_O (+0.2 % FA) to give **7** (12 mg, t_R_ = 27.5 min). C4 (98 mg) was subsequently purified by a Sephadex LH-20 column (2 × 90 cm) eluted with H_2_O-MeOH system (5:5) to yield one fraction: C4-1 (33 mg). C4-1 was subsequently purified by preparative reverse-phase HPLC eluted with 12 % MeCN/H_2_O (+0.2 % FA) to give compound **5** (8 mg, t_R_ = 25.5 min) and **6** (16 mg, t_R_ = 26.5 min).

B6 eluted with MeOH was purified by a Sephadex LH-20 column (4 × 150 cm), to yield five fractions: E1–E5. E2 was purified by preparative reverse-phase HPLC eluted with 17 % MeCN/H_2_O (+0.2 % FA) to give compound 11 (t_R_ = 16.5 min) and compound 10 (t_R_ = 23.5 min), then compounds **10** and **9** were purified by a Sephadex LH-20 column (2 × 40 cm) eluted with MeOH system to give compounds **10** (7 mg) and **9** (9 mg), respectively. E3 was purified by preparative reverse-phase HPLC eluted with 18 % MeCN/H_2_O (+0.2 % FA) to give compounds **14** (t_R_ = 26.5 min) and **8** (t_R_ = 30.5 min), and then compounds **14** and **8** were purified by a Sephadex LH-20 column (2 × 40 cm) eluted with MeOH system to give compounds **14** (8 mg) and **8** (6.5 mg), respectively. E4 was chromatographed on a Sephadex LH-20 column (2 × 150 cm) eluted with MeOH system to give E4-1 and E4-2, E4-2 was purified by preparative reverse-phase HPLC eluted with 37 % MeOH/H_2_O (+0.2 % FA) to give compound **18** (7 mg, t_R_ = 29 min) and E4-1 was chromatographed on a Sephadex LH-20 column (2 × 150 cm) eluted with MeOH system to give compound **22** (10 mg).

B7 was purified with a Sephadex LH-20 column (2 × 150 cm) eluted with MeOH system, and then four fractions (B7-1, B7-2, B7-3 and B7-4) were got. B7-2 was prepared on reverse-phase HPLC eluted with 41 % MeOH/H_2_O (+0.2 % FA) to give compound **19** (7 mg, t_R_ = 21 min), B7-3 was prepared on reverse-phase HPLC eluted with 35 % MeOH/H_2_O (+0.2 % FA) to give compound **20** (7 mg, t_R_ = 20 min).

B8 was chromatographed on a Sephadex LH-20 column (2 × 150 cm) eluted with MeOH system and then was purified by preparative reverse-phase HPLC eluted with 50 % MeOH/H_2_O (+0.2 % FA) to give compound **17** (6 mg, t_R_ = 31 min).

B9 was chromatographed on a Sephadex LH-20 column (2 × 150 cm) eluted with MeOH system and then was purified by preparative reverse-phase HPLC eluted with 55 % MeOH/H_2_O (+0.2 % FA) to give compound **21** (7 mg, t_R_ = 29 min).

**Compound 1:**

5, 7-dihydroxy-8-((2R)-2-methylbutan-1-onyl)-methyl phenylacetate.

Colorless noodle-like crystal, C_14_H_18_O_5_, [*α*]_*D*_^15^ − 9.5° (c 0.5, CHCl_3_), HR-ESI MS (positive ion mode) m/z: 289.1051 [M + Na]^+^ (calcd. for C_14_H_18_O_5_Na, 289.1052). ^1^ H (500 M, CD_3_OD) and ^13^ C (125 MHz, CD_3_OD) NMR data, see Table 1.

**Compound 2:**

5, 7-dihydroxy-8-((2R)-2-methylbutan-1-onyl)-phenylacetic acid 7-O-β-D-glucopyranoside.

White amorphous powder (MeOH), C_19_H_26_O_10_, HR-ESI MS (positive ion mode) m/z: 437.1390 [M + Na]^+^ (calcd. for C_19_H_26_O_10_Na, 437.1424). ^1^H (500 M, DMSO-*d*_*6*_) and ^13^C (125 MHz, DMSO-*d*_*6*_) NMR data, see Table 1.

### Neuraminidase inhibition activity

NA inhibitory activity was determined by the commercial NA inhibitory screening kit (P0309, Beyotime Institute of Biotechnology, Jiangsu, China). The compound 2’-(4-methylumbelliferyl)-a-D-acetylneuraminic acid (MUNANA) is the substrate of NA. And cleavage of this substrate by NA produces a fluorescent product, 322 nm was the excitation wavelength and 450 nm was the emission wavelength. The intensity of fluorescence can reflect the activity of NA sensitively. The IC_50_ was calculated by plotting percent inhibition versus the inhibitor concentration and determination of each point was performed in duplicate. The actual and detailed experimental which was prepared according to literature method [31].

The inhibition rates were calculated as follows: [A1–A(background)-[A2–A(background)]/[A1–A (background)] × 100], where A1 is the absorbance of the control, and A2 is the absorbance of the sample. IC_50_ was determined by plotting the percentage of NA activity against inhibitor concentration using software that came with the microplate reader. The values are expressed as the mean ± SD of triplicate experiments.

### Molecular docking

The cocrystal complex of N1 NA in complex with corresponding ligand oseltamivir downloaded from the protein data bank. (PDB ID code 2HU4) [32]. Before docking, the pre-existing ligand was removed out and hydrogen atoms and charges were added. The docking studies were performed using the Surflex-Dock module of Sybyl 8.1, and the maximum number of poses per ligand was set to 10. The active site of the protein was automatically explored and created based on the previous ligand oseltamivir by the Surflex-Dock Protomol Generation Programme, and other parameters were set as default.

## Conclusions

The two new compounds (**1**, **2**) and compounds **11**, **12**, **14**, **15**, **19** and **20** showed better inhibitory activity against NA in vitro. By comparing with the structures of compound **11**, **12**, **14**, **15**, **19** and **20**, they all have one caffeoyl, and this is a possible reason that these compounds have better inhibitory activity against NA than other polyphenolic compounds. With the help of molecular docking, we had a preliminary understanding of the mechanism of the two new compounds (**1–2**)′ NA inhibitory activity. According to the Lipinski’s rule of five, compound **1** may be a better lead compound for anti- influenza.

Fractions 6 and polyphenolic compounds isolated from fractions 6 showed higher NA inhibition than that of the initial plant exacts (Tables 2, 3). The findings of this study indicate that polyphenolic compounds and fractions 6 derived from *P. cablin* are potential NA inhibitors. This work was one of the evidence that *P. cablin* has better inhibitory activity against influenza, which not only enriches the compound library of *P. cablin*, but also facilitates further development and promises its therapeutic potential for the rising challenge of influenza diseases.

## Additional files

10.1186/s13065-016-0192-x Spectra of isolated compounds **1**–**22**.
10.1186/s13065-016-0192-x The data of NA inhibition experiments.
