# Supplementary material for: Polyphenolic glycosides isolated from Pogostemon cablin (Blanco) Benth. as novel influenza neuraminidase inhibitors
Source: Chem Cent J. 2016 Aug 10;10:51. doi: 10.1186/s13065-016-0192-x (PMC4980783; doi:10.1186/s13065-016-0192-x)
Supplement: Supplementary file 2 — 10.1186/s13065-016-0192-x The data of NA inhibition experiments. [file 13065_2016_192_MOESM2_ESM.pdf]

Supplementary Materials: polyphenolic glycosides isolated from *Pogostemon cablin* (Blanco) Benth. as novel influenza neuraminidase inhibitors

Fang LIU[1], Wei CAO[1], Chao DENG[1], Zhaoquan WU[1], Guangyao ZENG[1] and Yingjun ZHOU*[1]

Figure S1. ^{1}H-NMR spectrum (500 MHz) of compound 1 in CD_3OD.

Figure S2. ^{13}C-NMR spectrum (125 MHz) of compound 1 in CD_3OD.

Figure S3. HSQC spectrum (500 MHz) of compound 1 in CD_3OD.

Figure S4. HMBC spectrum (500 MHz) of compound 1 in CD_3OD.

Figure S5. ^{1}H-NMR spectrum (500 MHz) of compound 2 in DMSO.

Figure S6. ^{13}C-NMR spectrum (125 MHz) of compound 2 in DMSO.

Figure S7. ^{1}H-^{1}H COSY spectrum (500 MHz) of compound 2 in DMSO.

Figure S8. TOCSY spectrum (500 MHz) of compound 2 in DMSO.

Figure S9. HSQC spectrum (500 MHz) of compound 2 in DMSO.

Figure S10. HMBC spectrum (500 MHz) of compound 2 in DMSO.

Figure S11. HR ESI (+) MS spectrum of compound 1.

Figure S12. HR ESI (+) MS spectrum of compound 2.

Figure S13-62 ^{1}H-NMR spectrum (500 MHz), ^{13}C-NMR spectrum (125 MHz) and HR ESI (+) MS spectrum of the compounds 3-22.

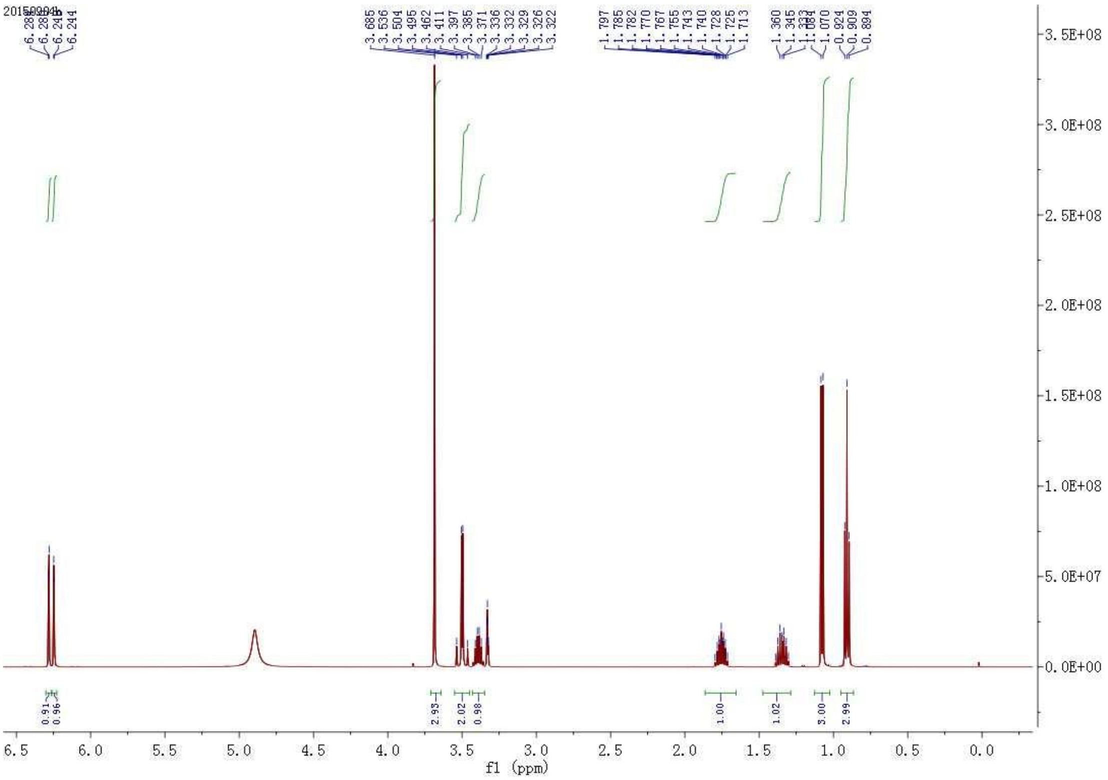

Figure S1. ^{1}H-NMR spectrum (500 MHz) of compound 1 in CD_3OD.

*Correspoundence : fisher203@126.com (Y. Z.)

[1] College of pharmacy, Central South University, Changsha 410013, PR China
Full list of author information is available at the end of the article.

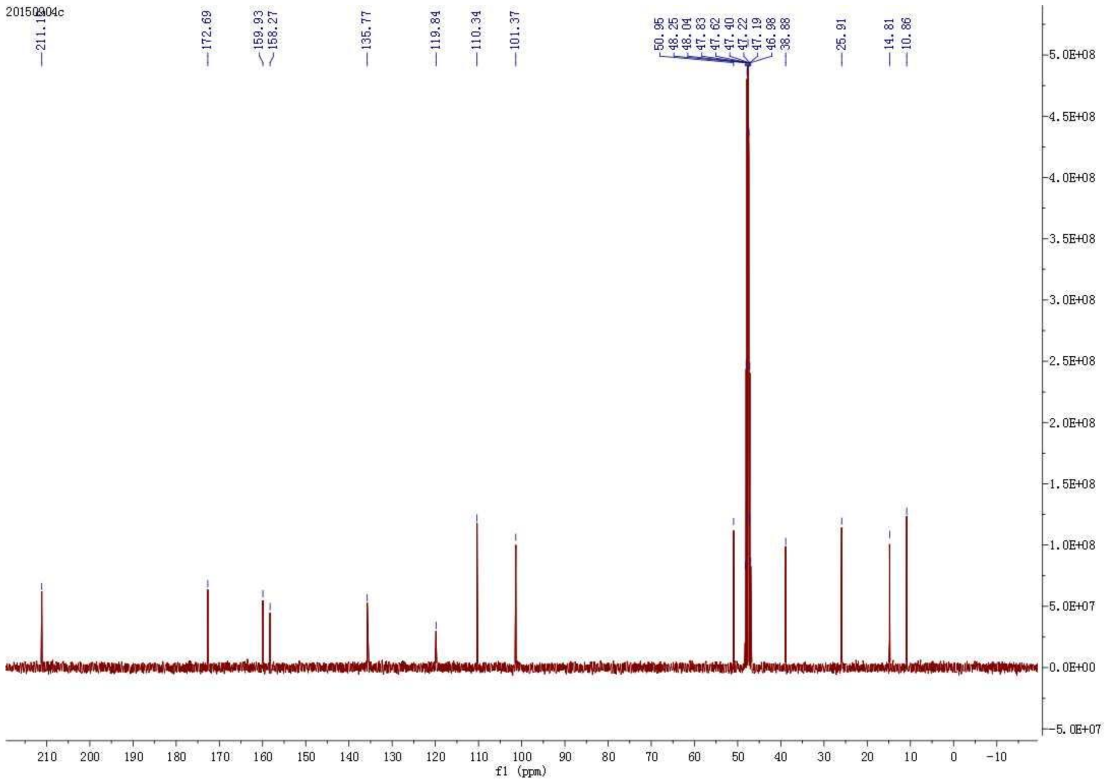

Figure S2. ^{13}C-NMR spectrum (125 MHz) of compound 1 in CD_3OD.

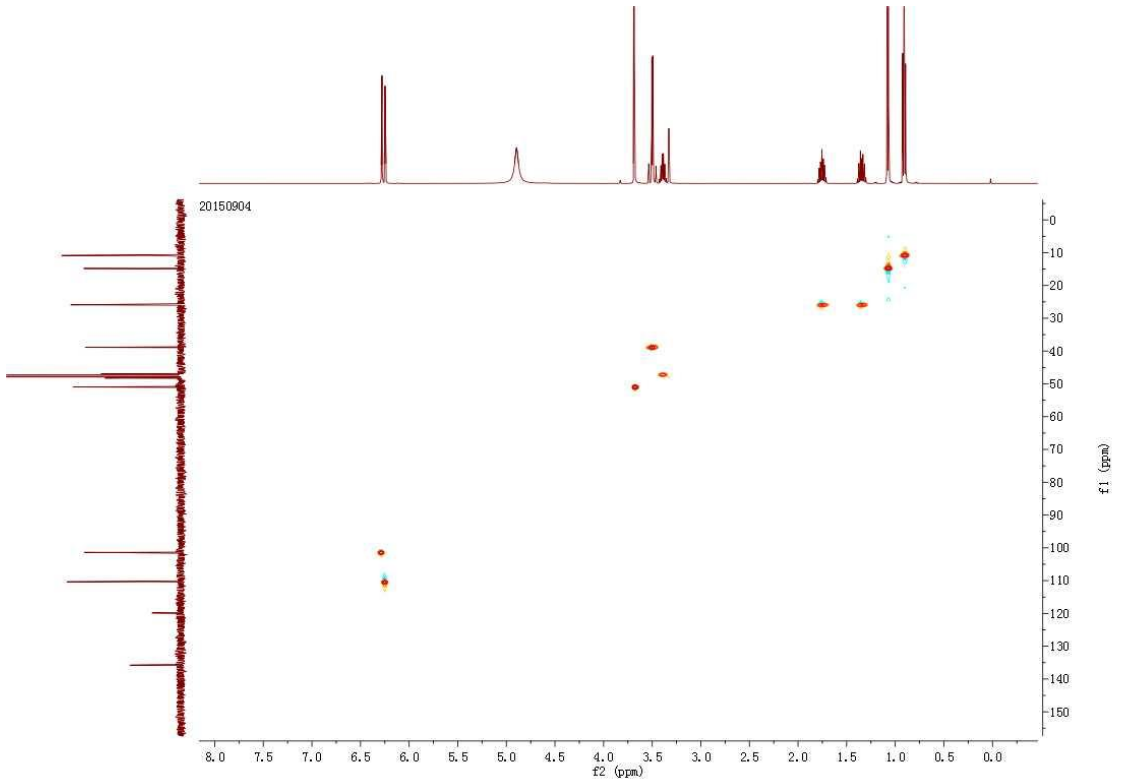

Figure S3. HSQC spectrum (500 MHz) of compound 1 in CD_3OD.

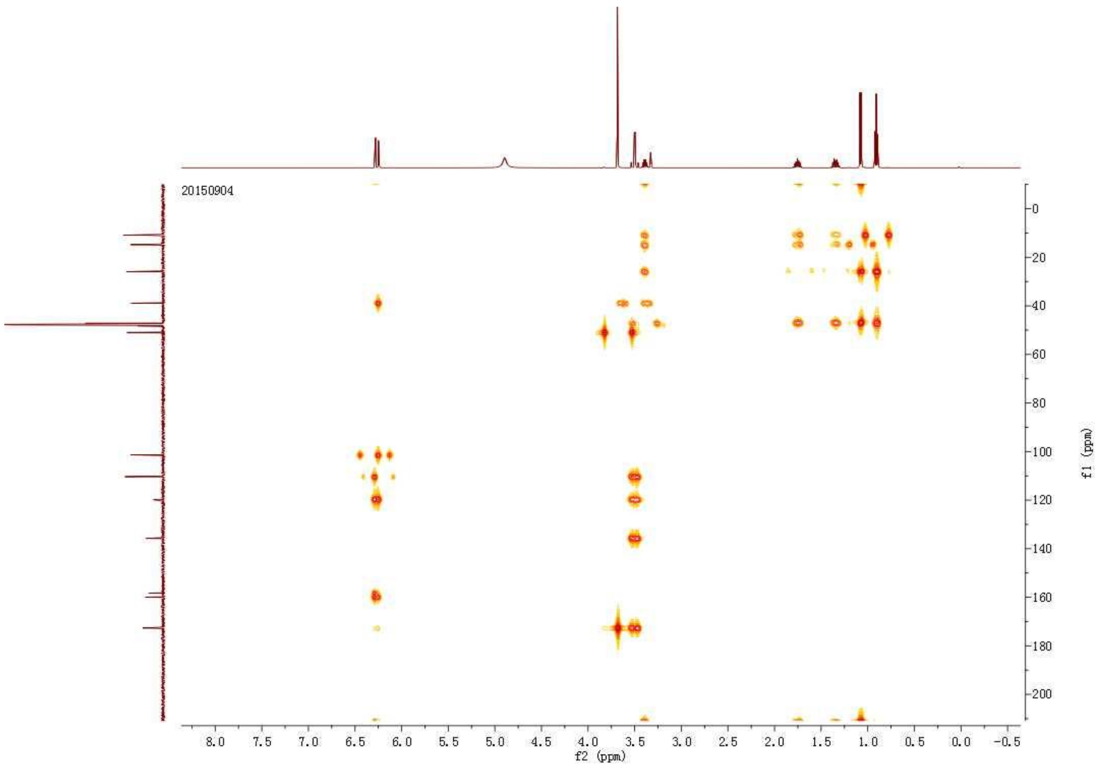

Figure S4. HMBC spectrum (500 MHz) of compound 1 in CD_3OD.

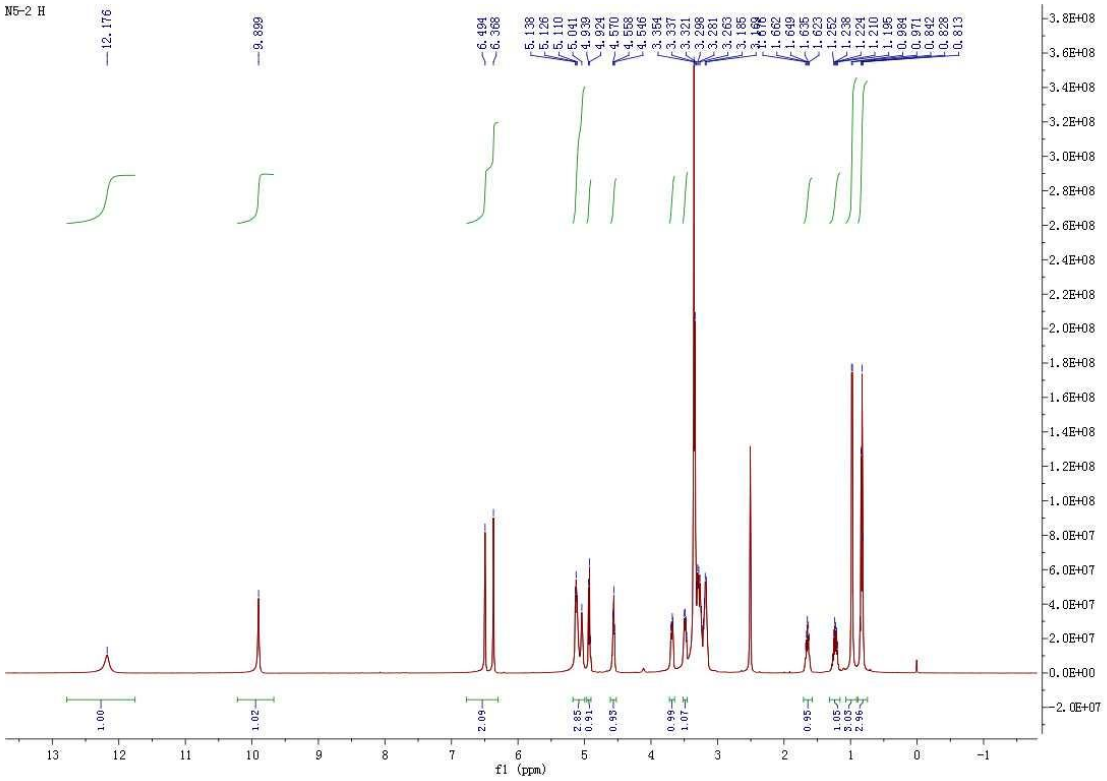

Figure S5. ^{1}H-NMR spectrum (500 MHz) of compound 2 in DMSO.

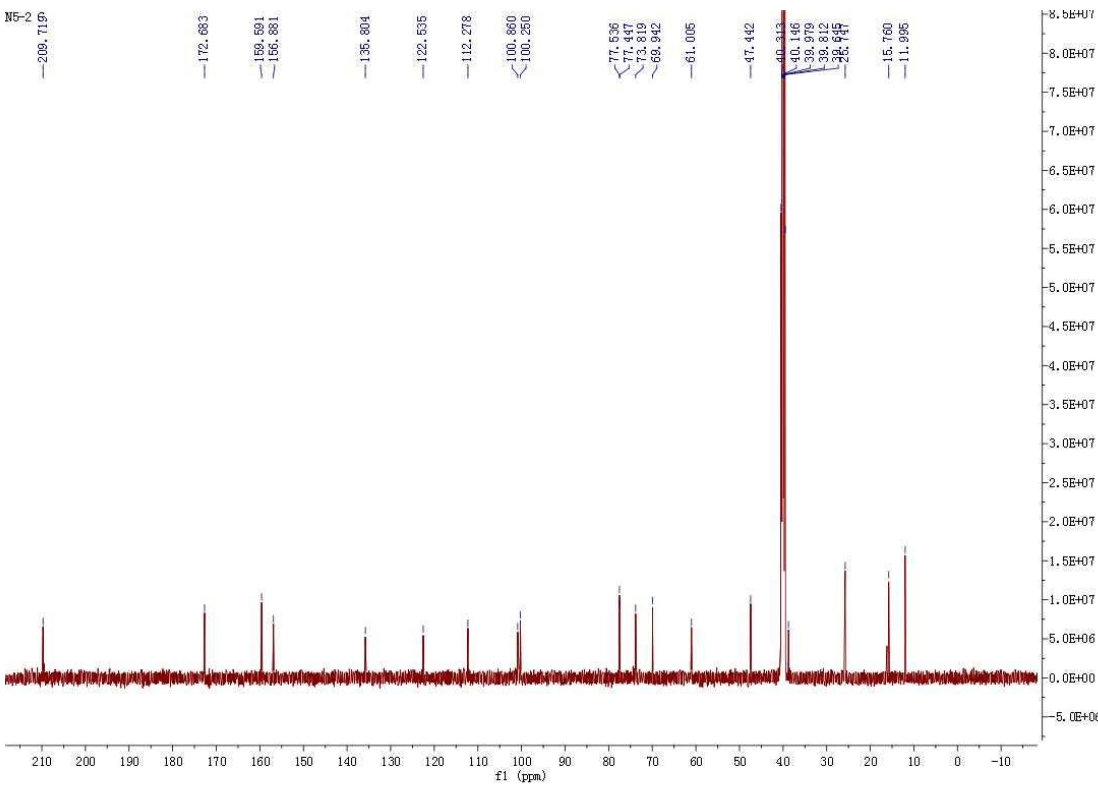

Figure S6. ^{13}C-NMR spectrum (125 MHz) of compound 2 in DMSO.

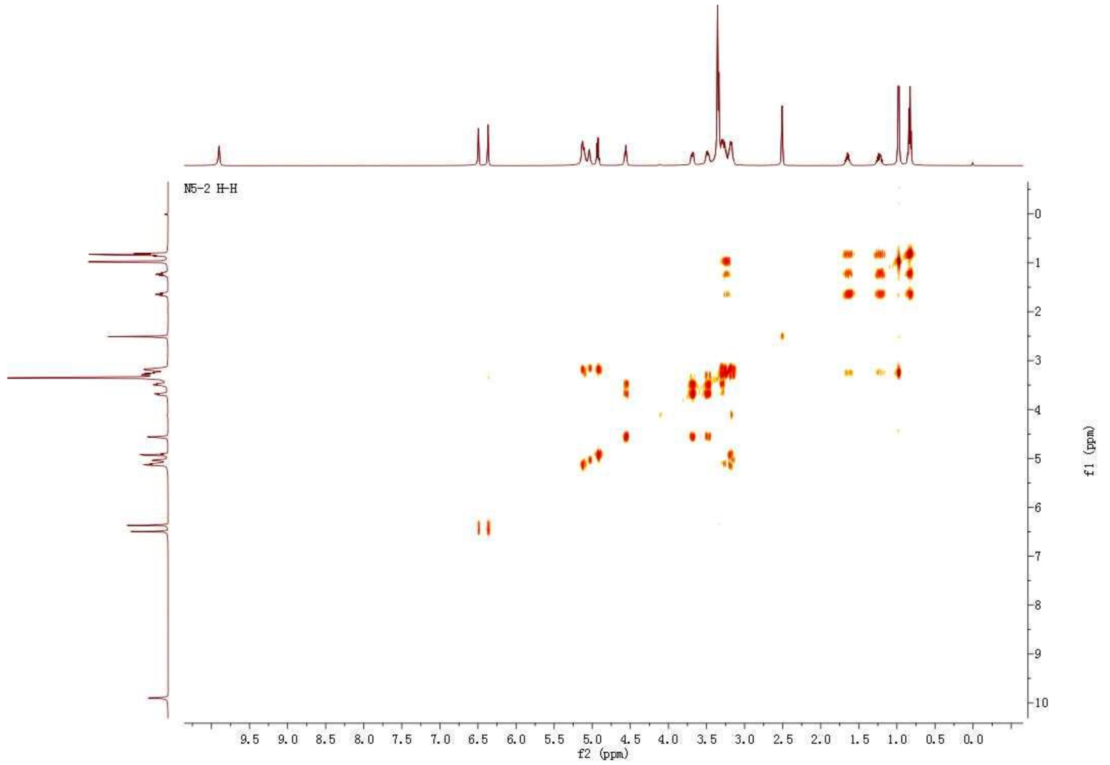

Figure S7. ^{1}H- ^{1}H COSY spectrum (500 MHz) of compound 2 in DMSO.

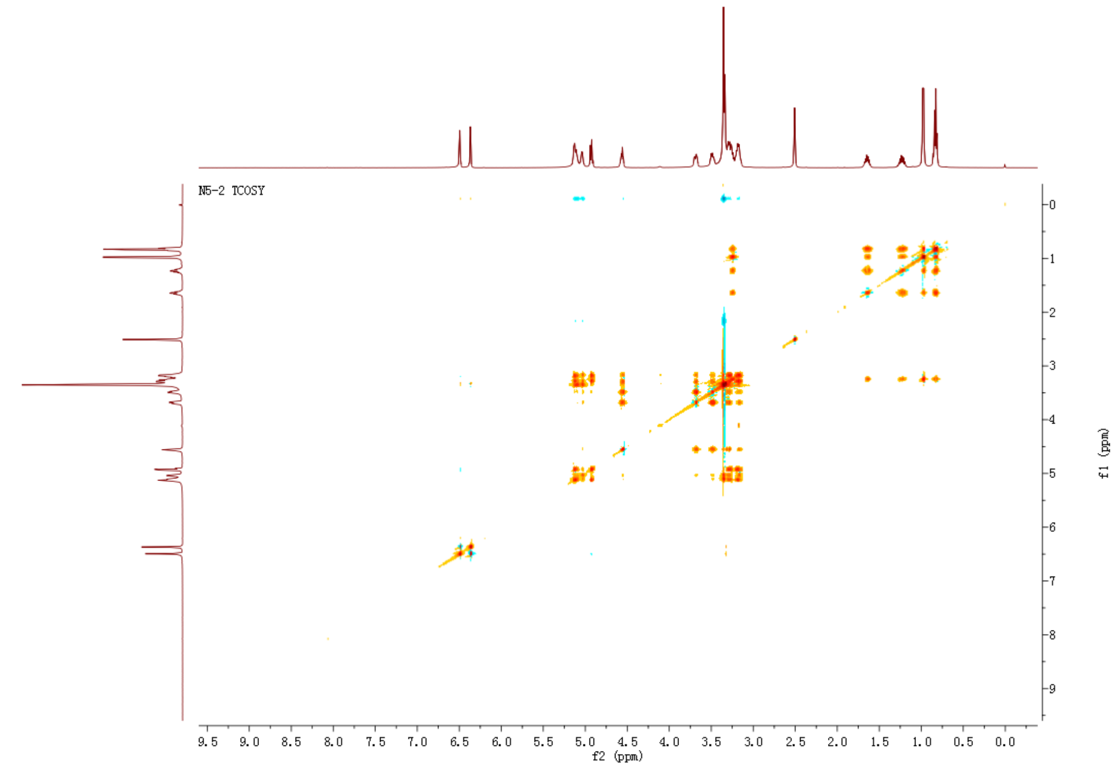

Figure S8. TOCSY spectrum (500 MHz) of compound 2 in DMSO.

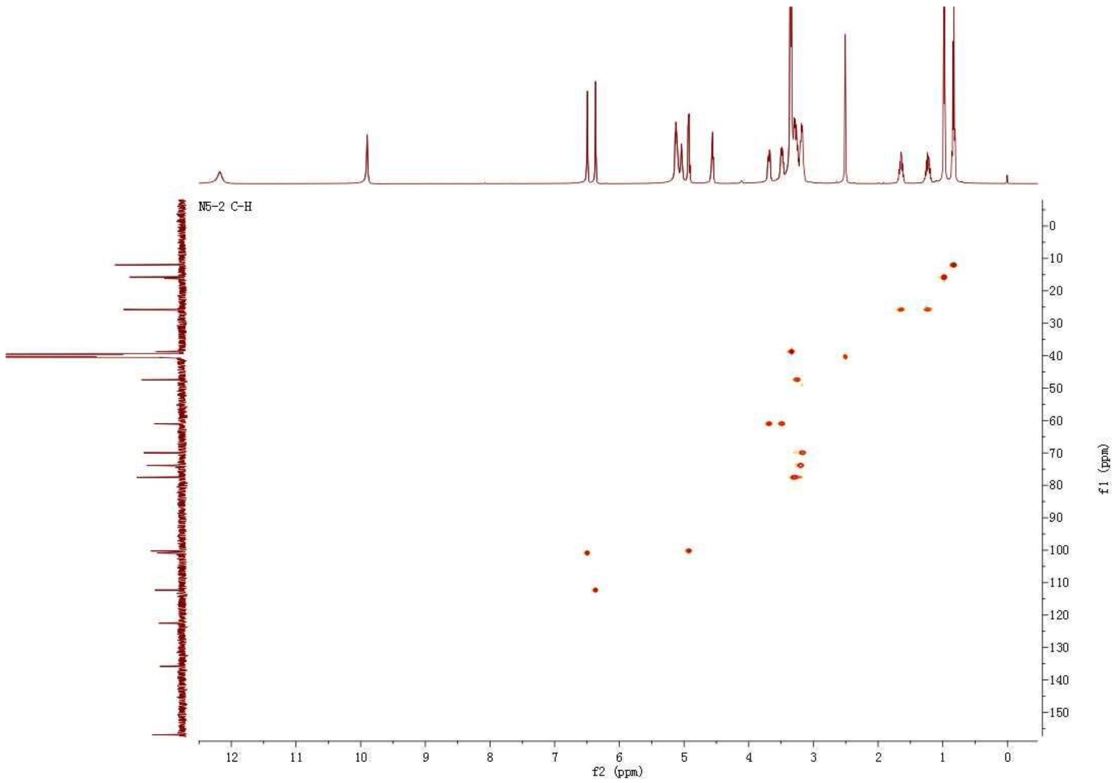

Figure S9. HSQC spectrum (500 MHz) of compound 2 in DMSO.

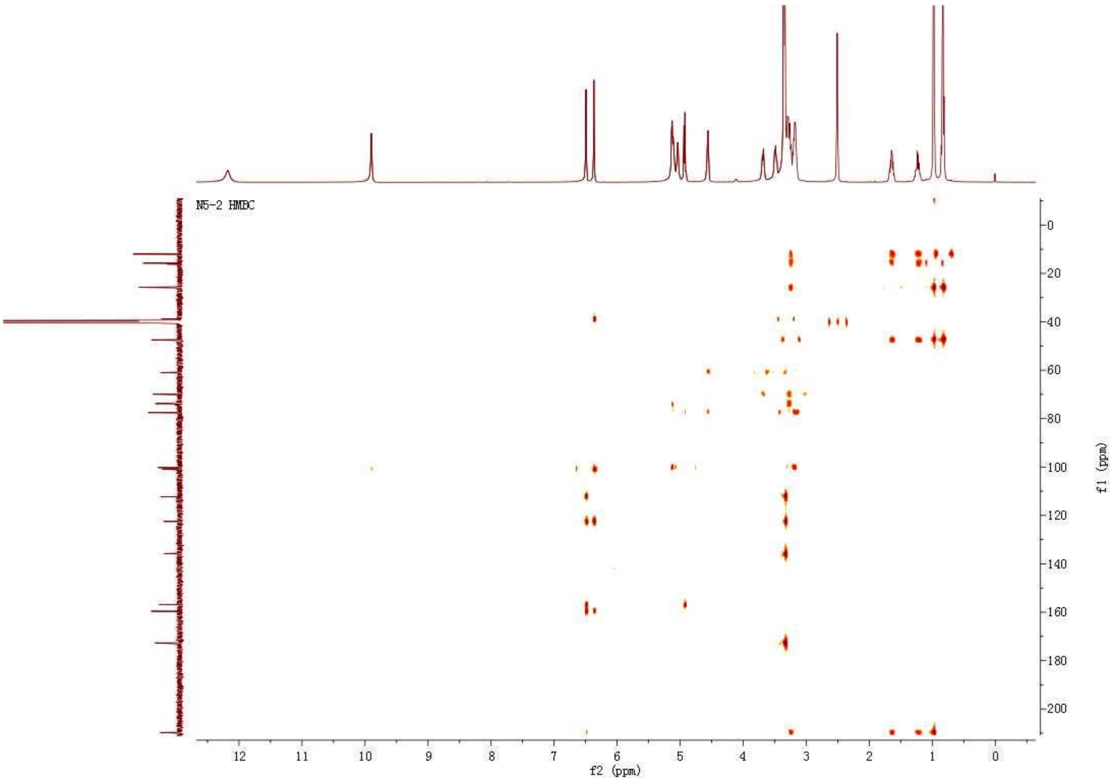

Figure S10. HMBC spectrum (500 MHz) of compound 2 in DMSO.

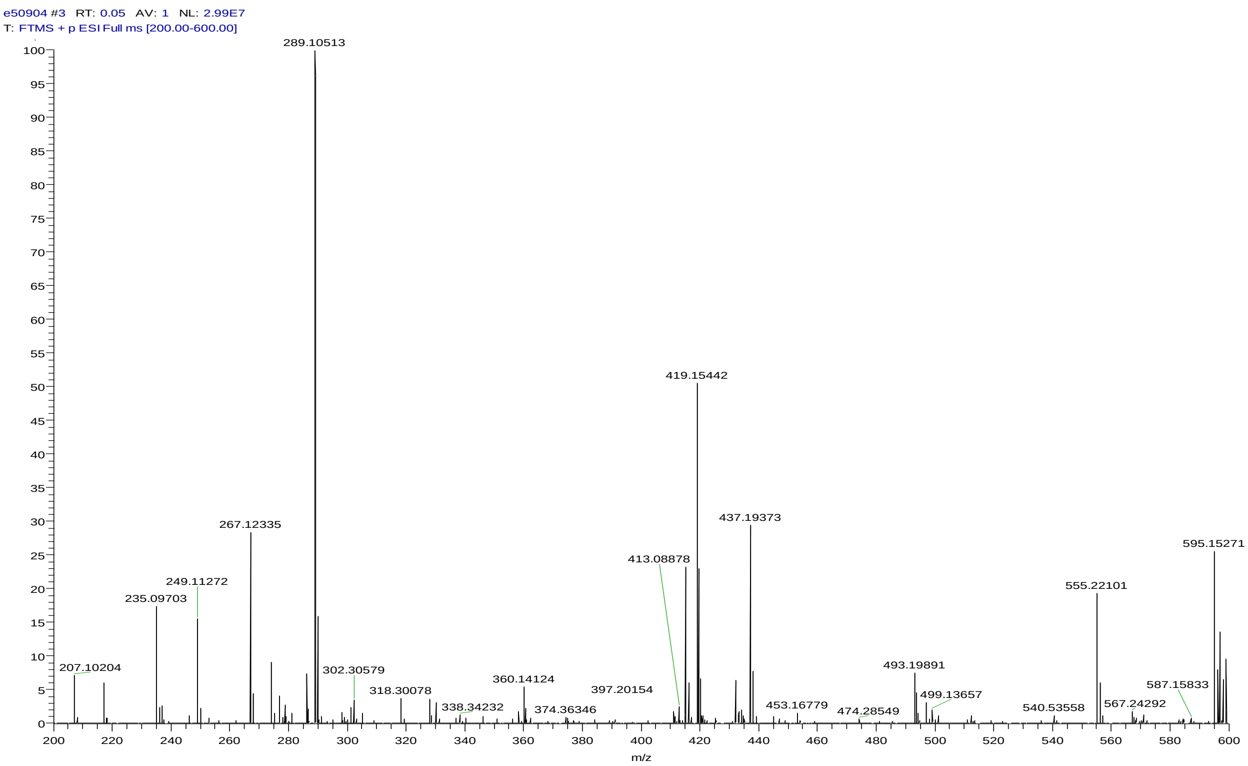

Figure S11. HR ESI (+)MS spectrum of compound 1.

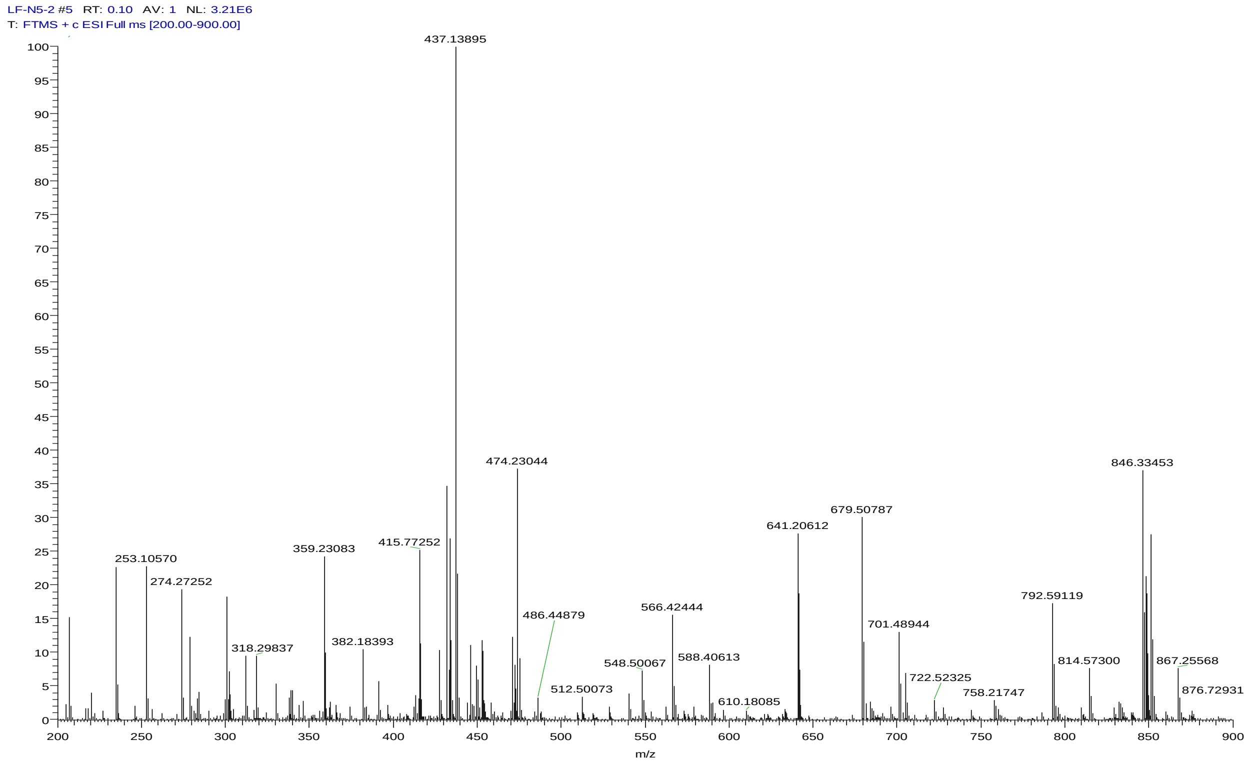

Figure S12. HR ESI (+)MS spectrum of compound 2.

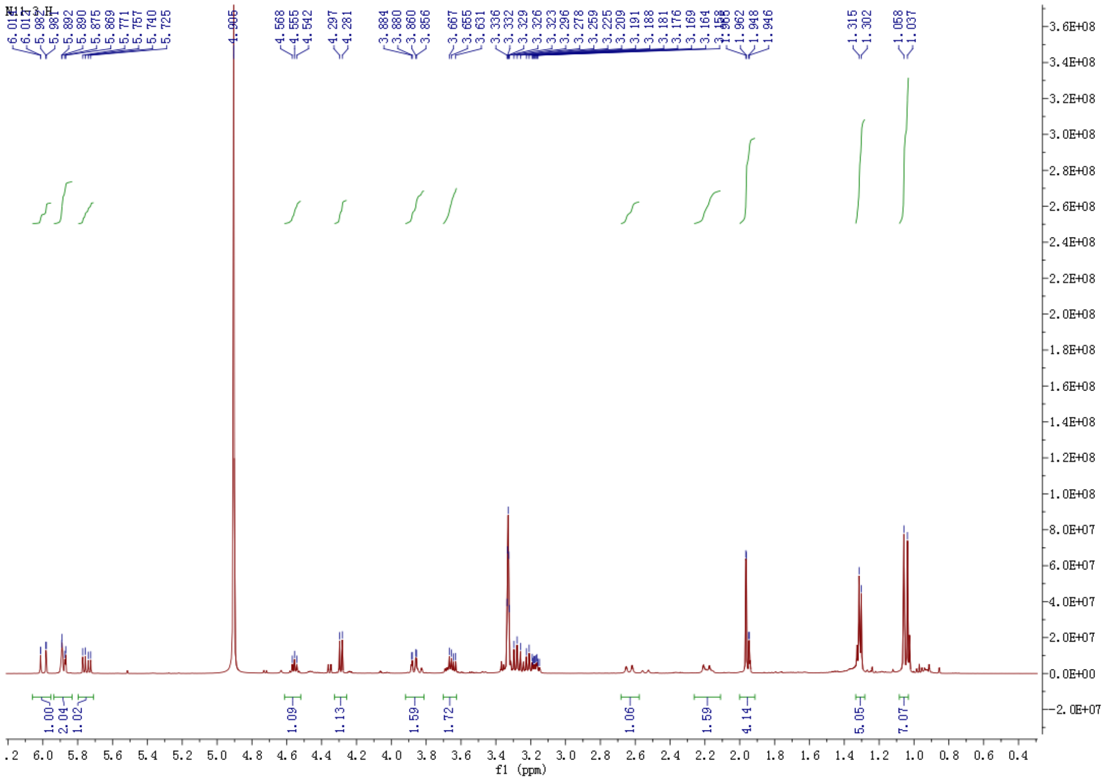

Figure S13. ^{1}H-NMR spectrum (500 MHz) of compound 3 in CD_3OD.

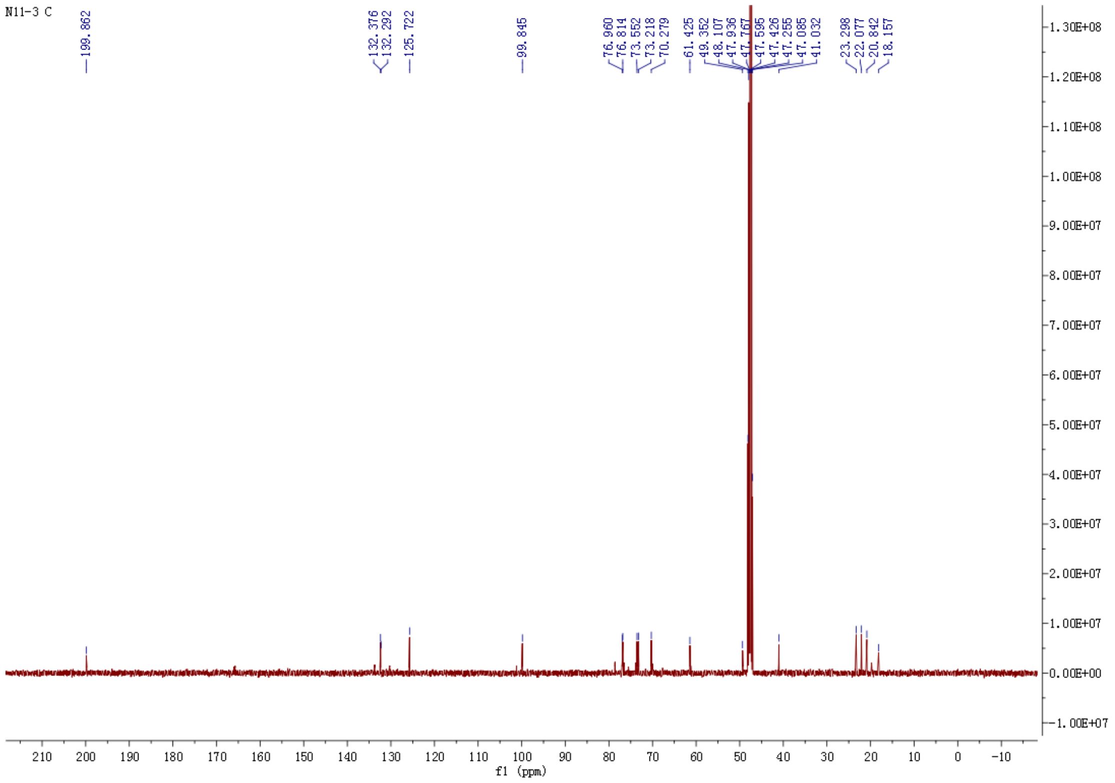

Figure S 14. ^{13}C-NMR spectrum (125 MHz) of compound 3 in CD_3OD.

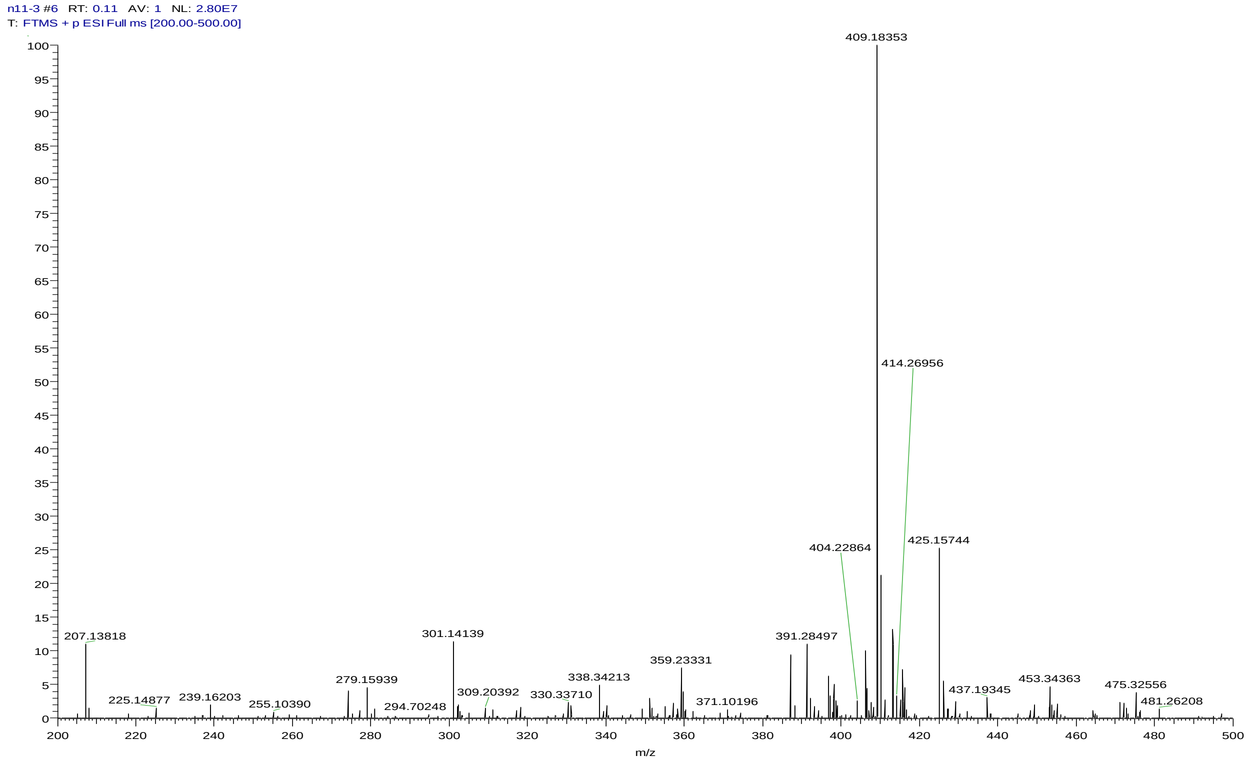

Figure S 15. HR ESI (+) MS spectrum of compound 3.

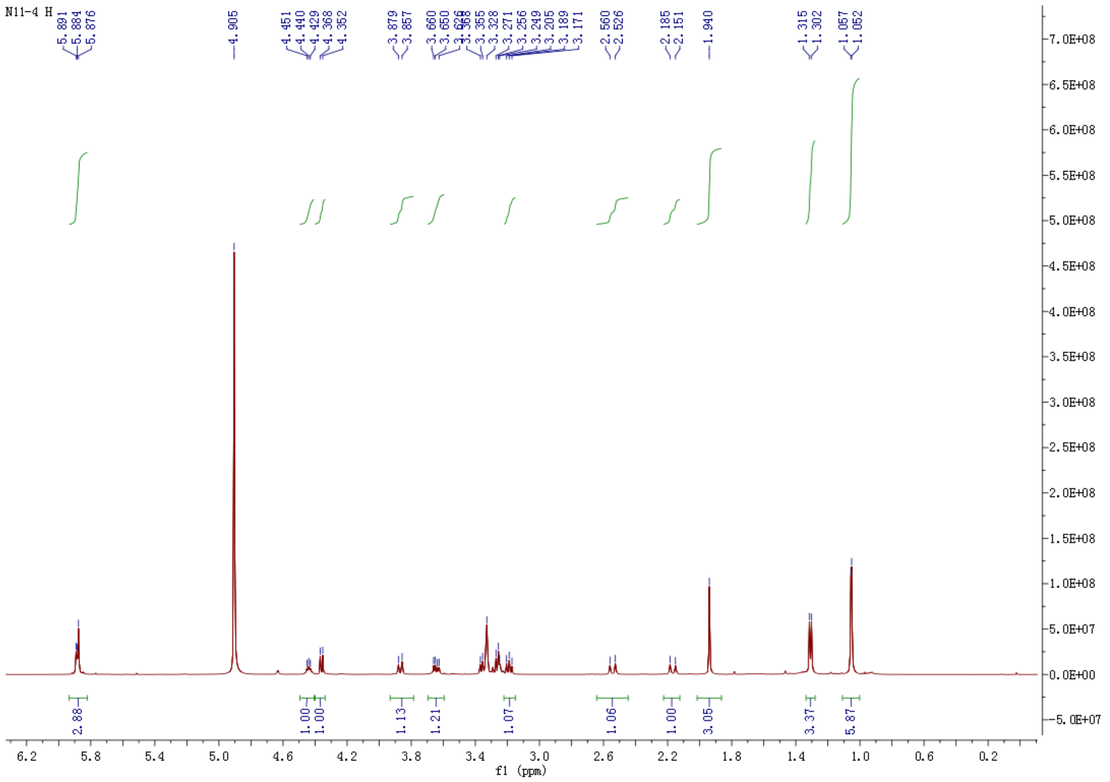

Figure S16. ^{1}H-NMR spectrum (500 MHz) of compound 4 in CD_3OD.

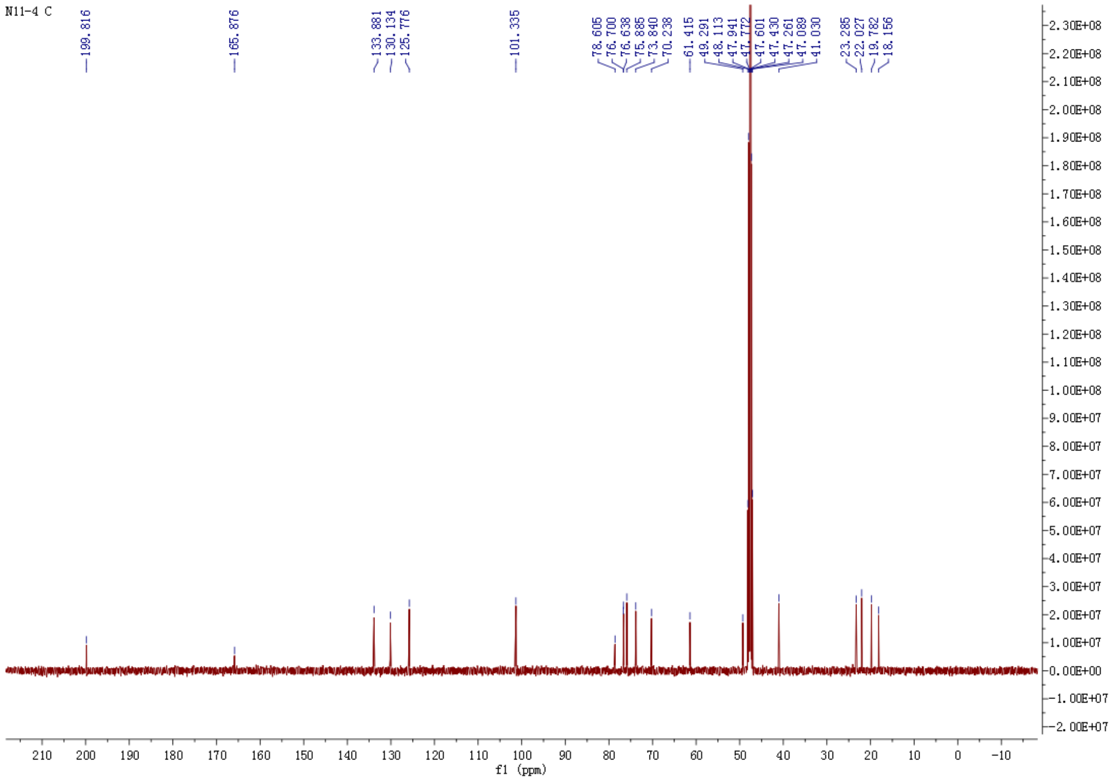

Figure S 17. ^{13}C-NMR spectrum (125 MHz) of compound 4 in CD_3OD.

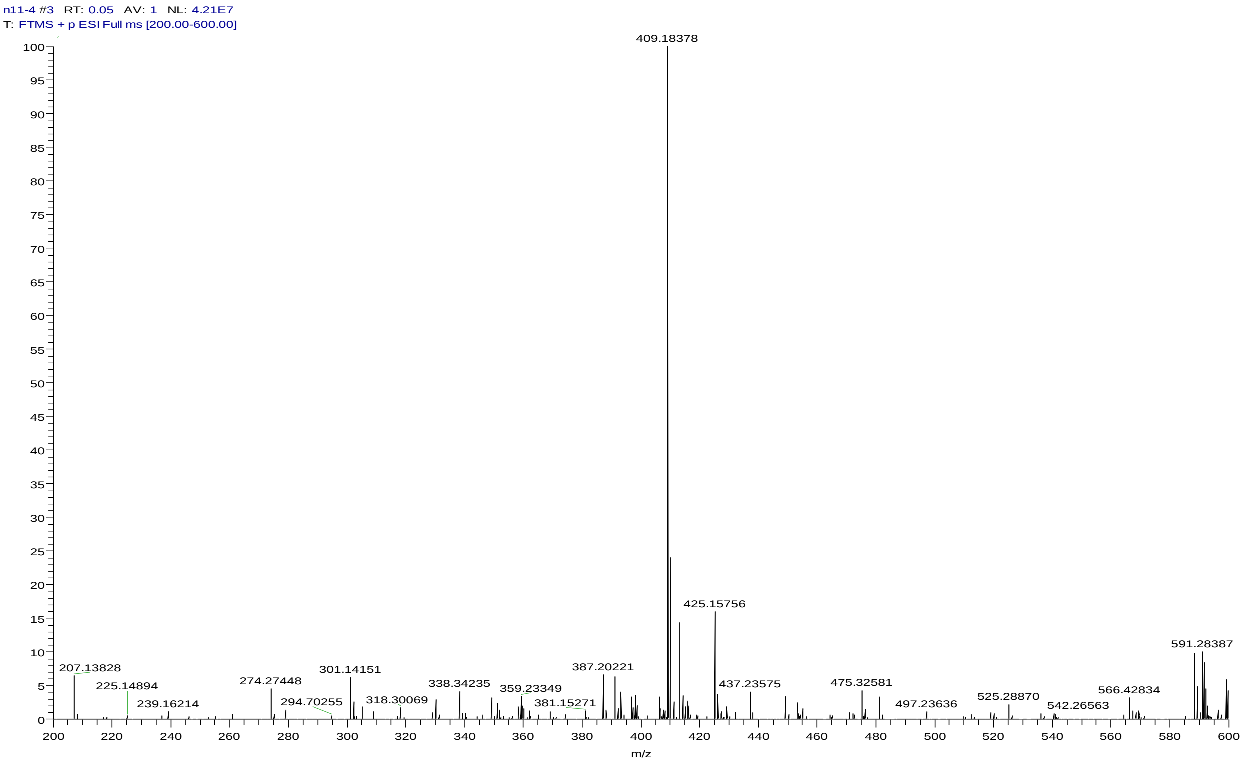

Figure S18. HR ESI (+) MS spectrum of compound 4.

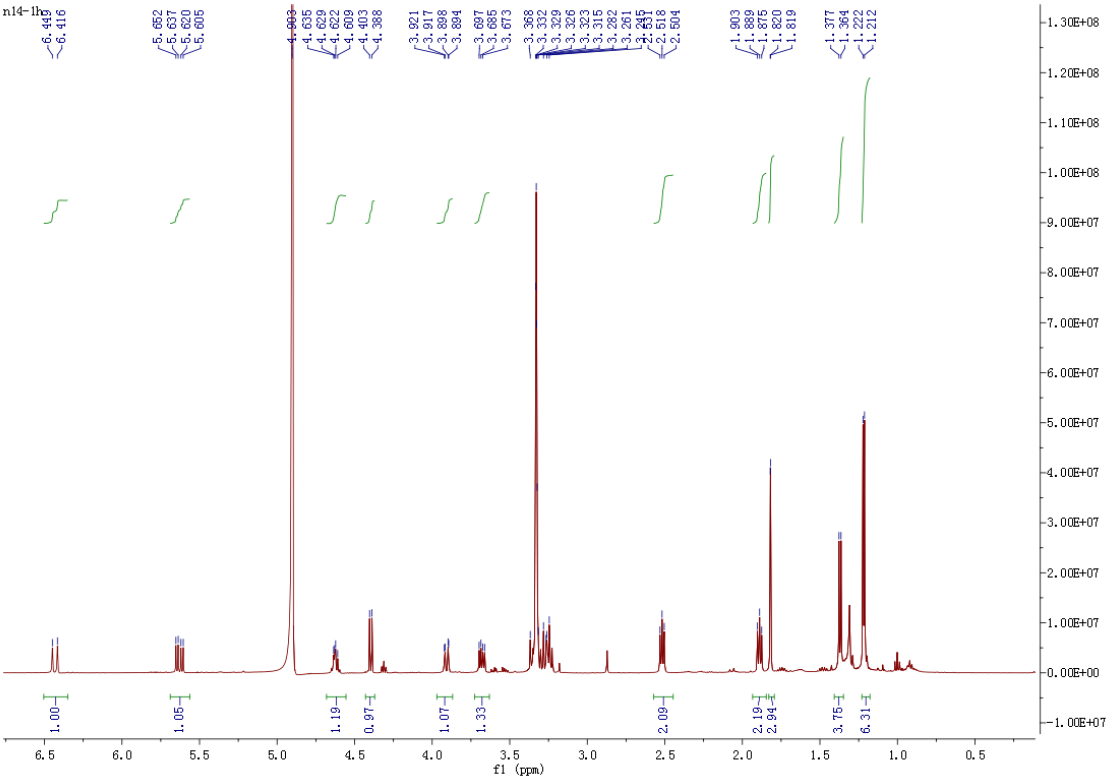

Figure S19. ^{1}H-NMR spectrum (500 MHz) of compound 5 in CD_3OD.

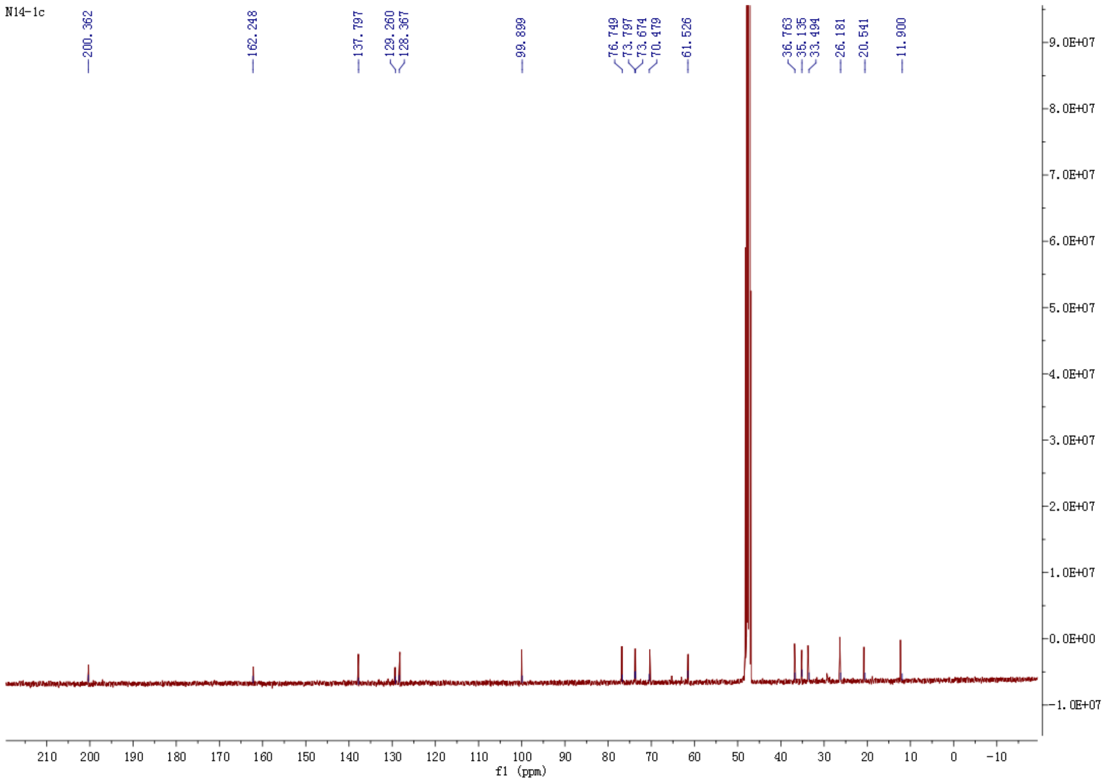

Figure S 20. ^{13}C-NMR spectrum (125 MHz) of compound 5 in CD_3OD.

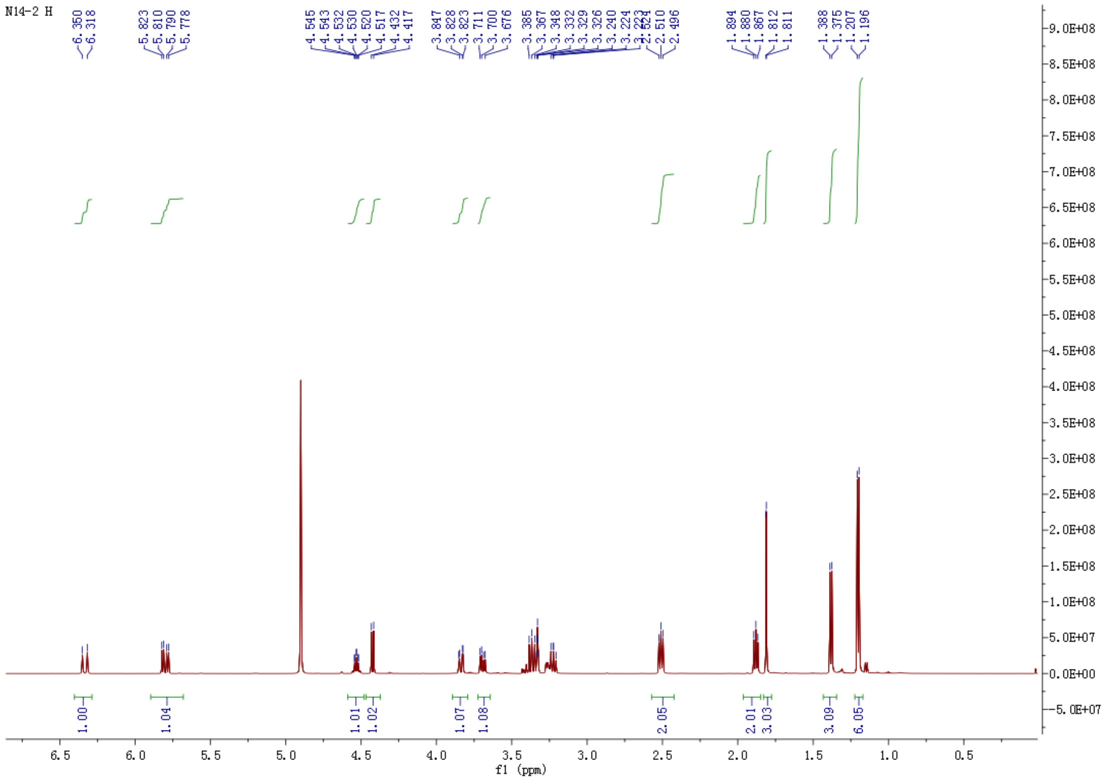

Figure S21. ^{1}H-NMR spectrum (500 MHz) of compound 6 in CD_3OD.

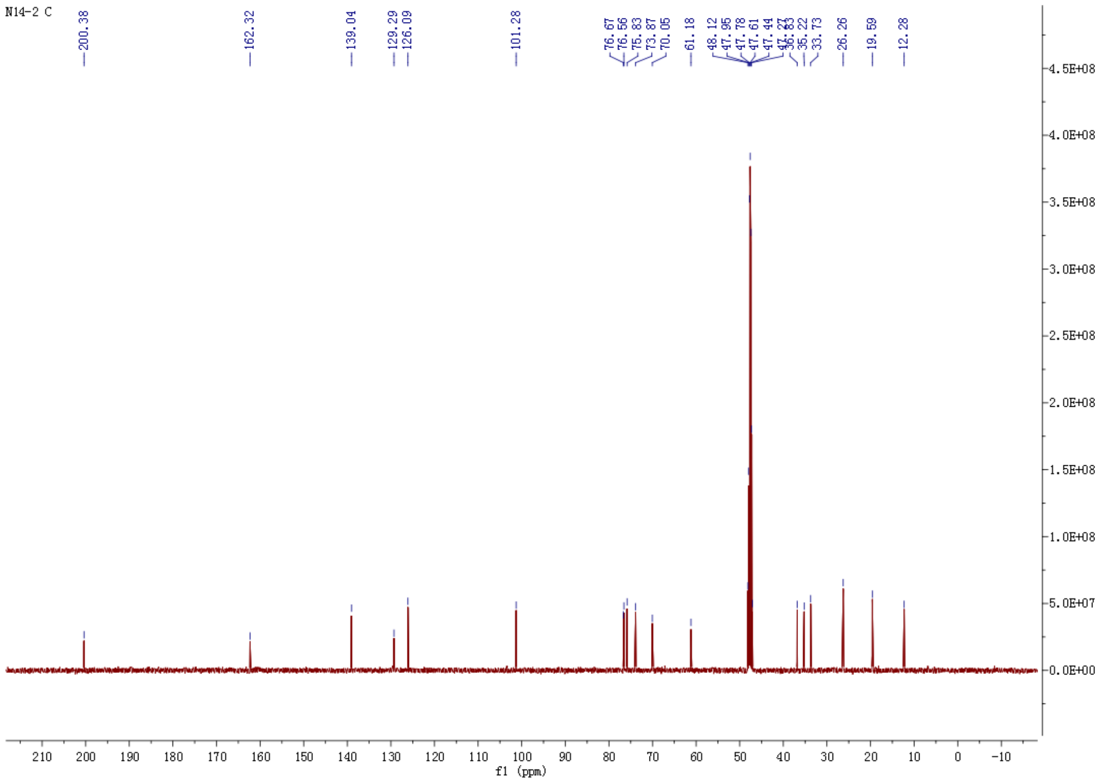

Figure S 22. ^{13}C-NMR spectrum (125 MHz) of compound 6 in CD_3OD.

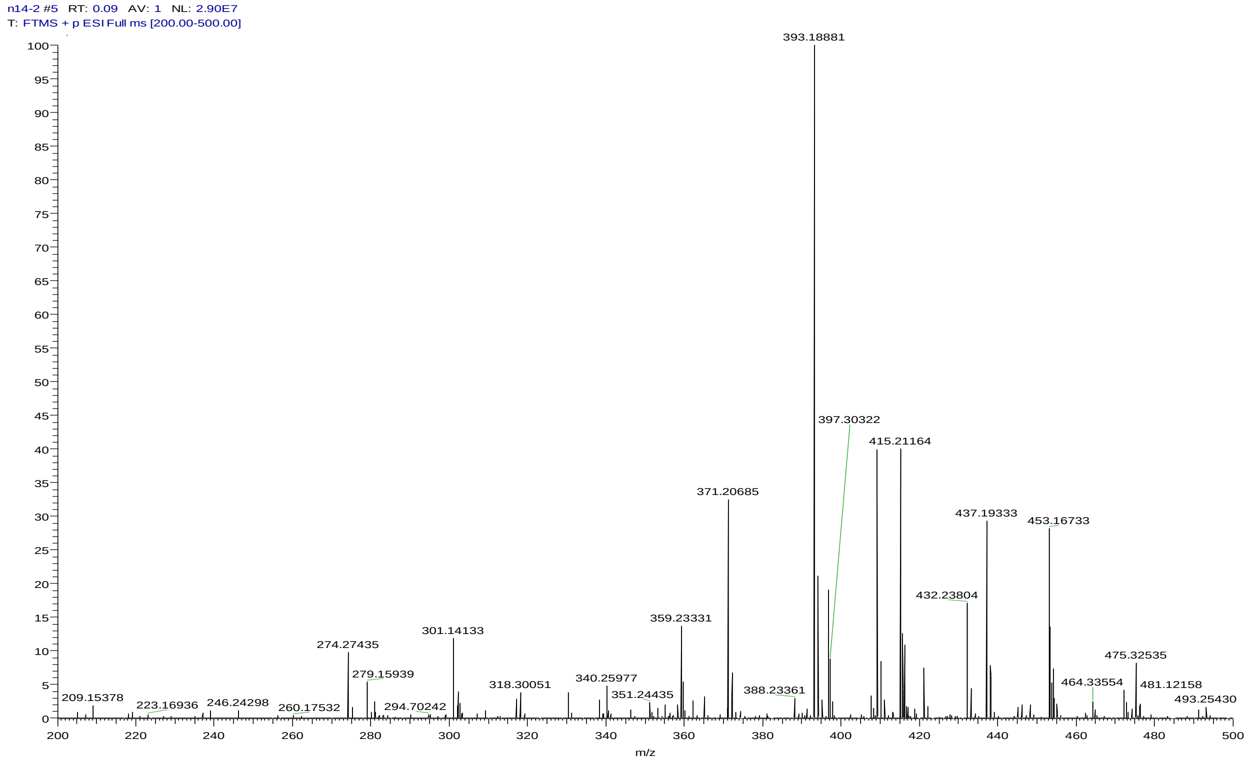

Figure S23. HR ESI (+) MS spectrum of compound 6.

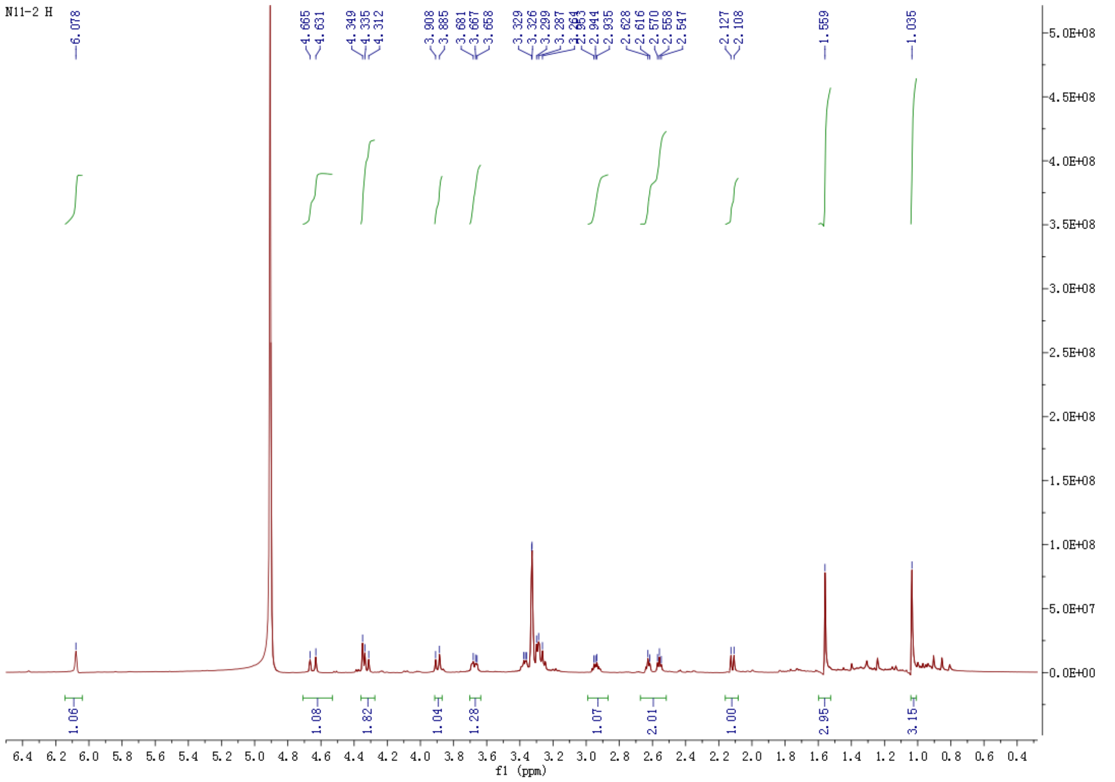

Figure S24. ^{1}H-NMR spectrum (500 MHz) of compound 7 in CD_3OD.

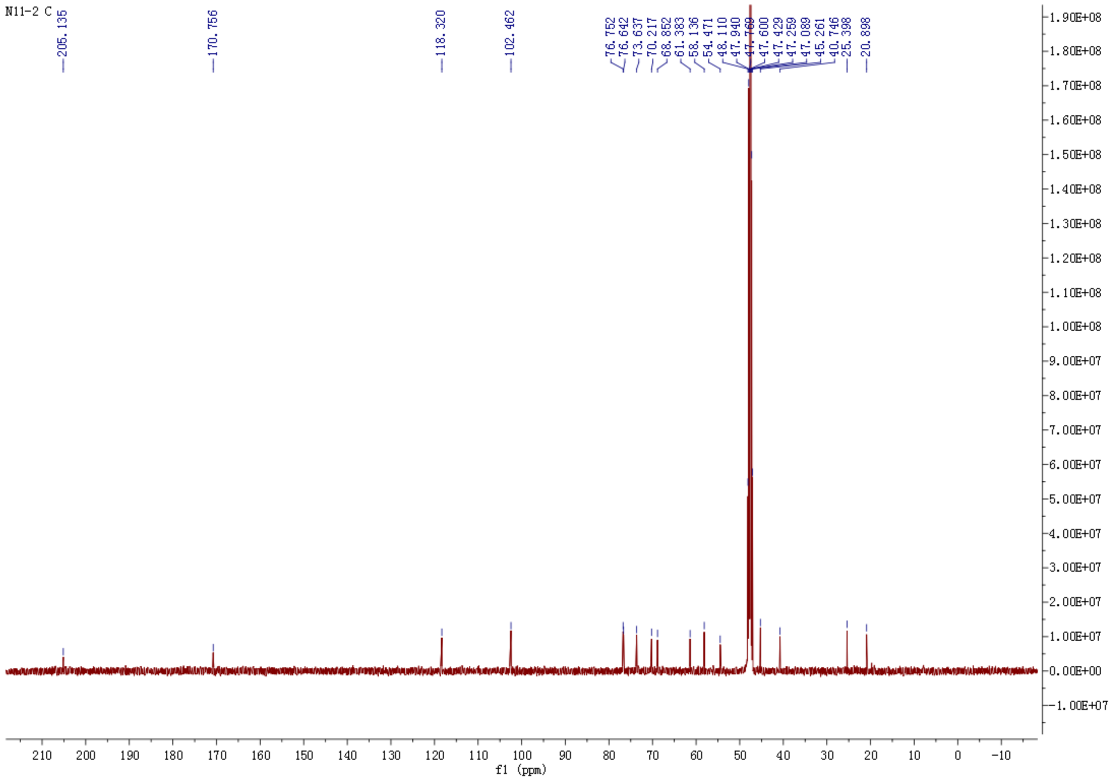

Figure S 25. ^{13}C-NMR spectrum (125 MHz) of compound 7 in CD_3OD.

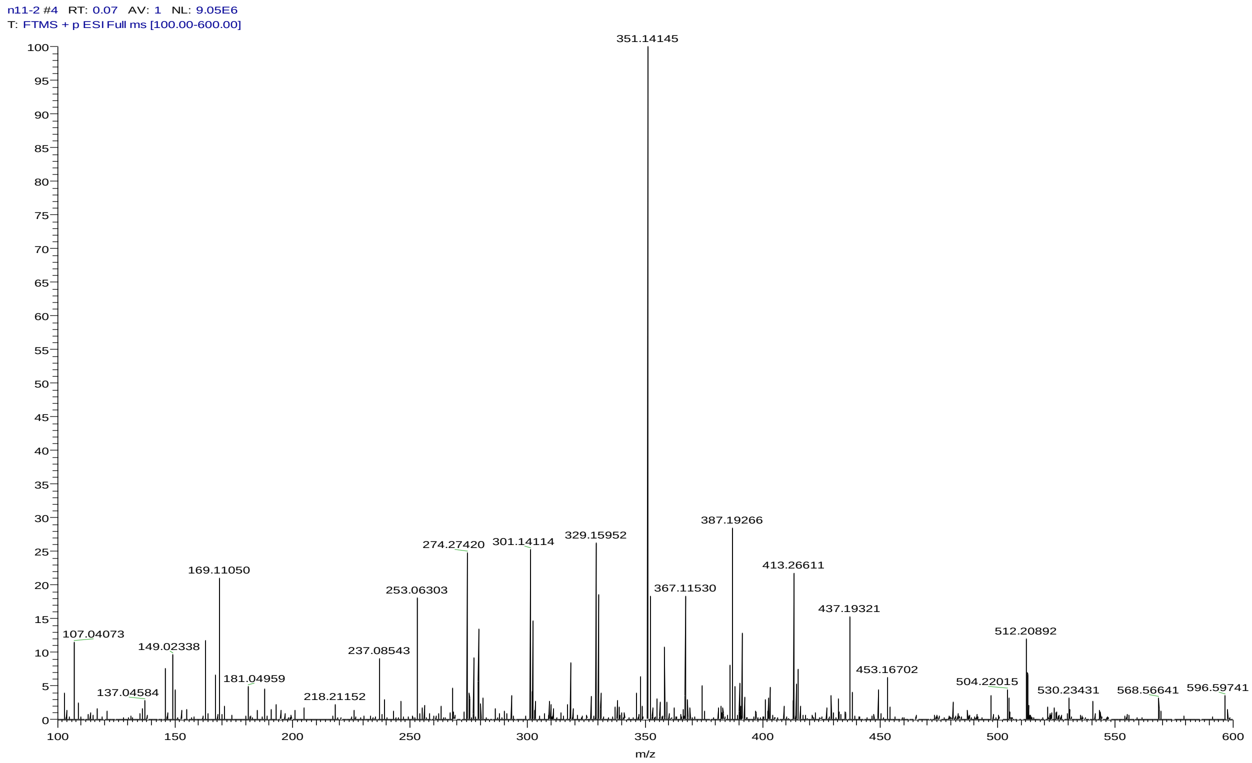

Figure S26. HR ESI (+) MS spectrum of compound 9.

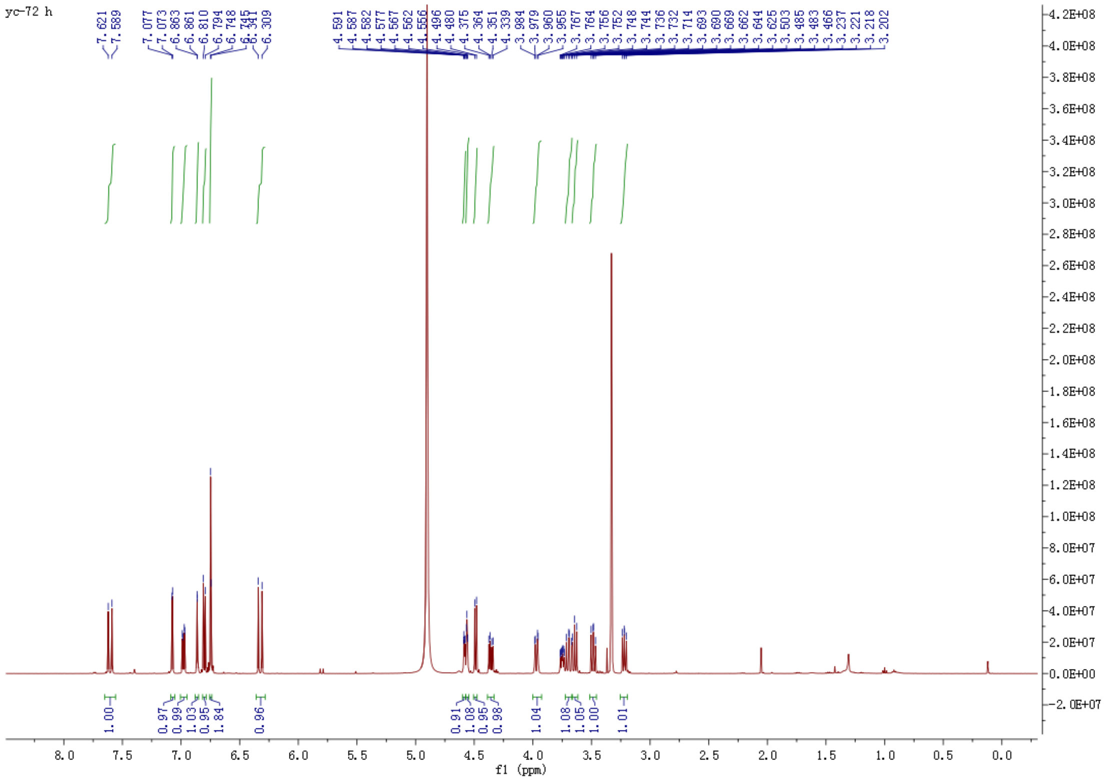

Figure S27. ^{1}H-NMR spectrum (500 MHz) of compound 8 in CD_3OD.

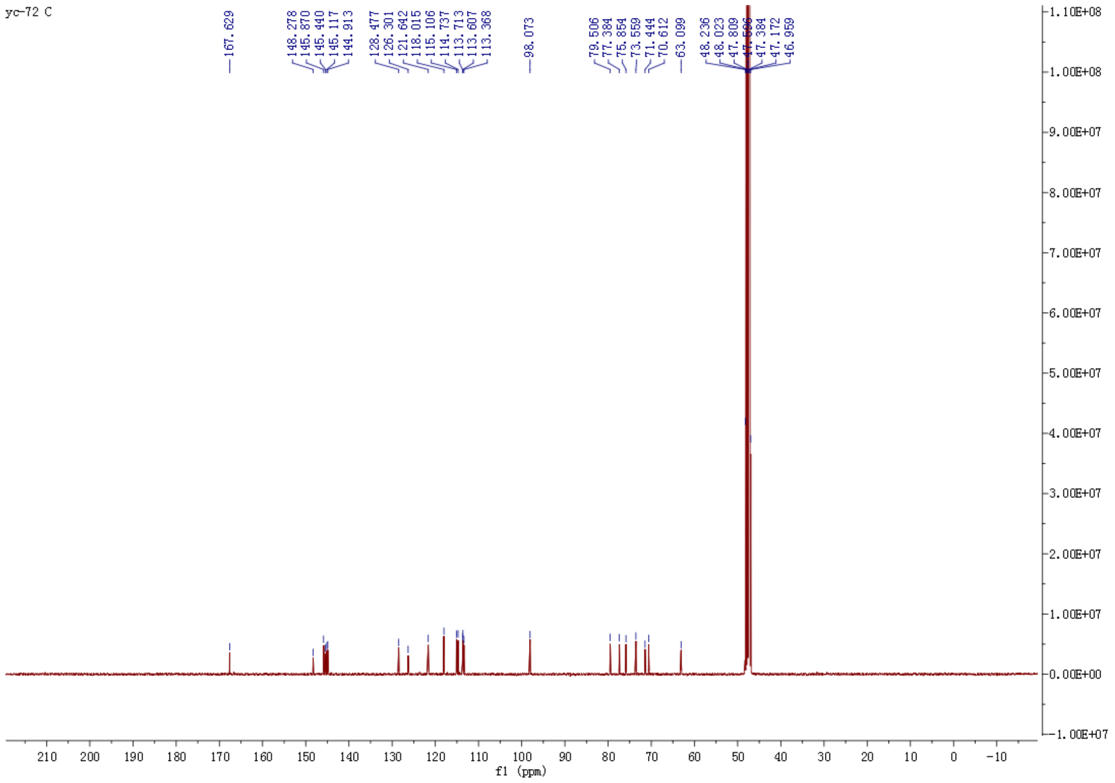

Figure S 28. ^{13}C-NMR spectrum (125 MHz) of compound 8 in CD_3OD.

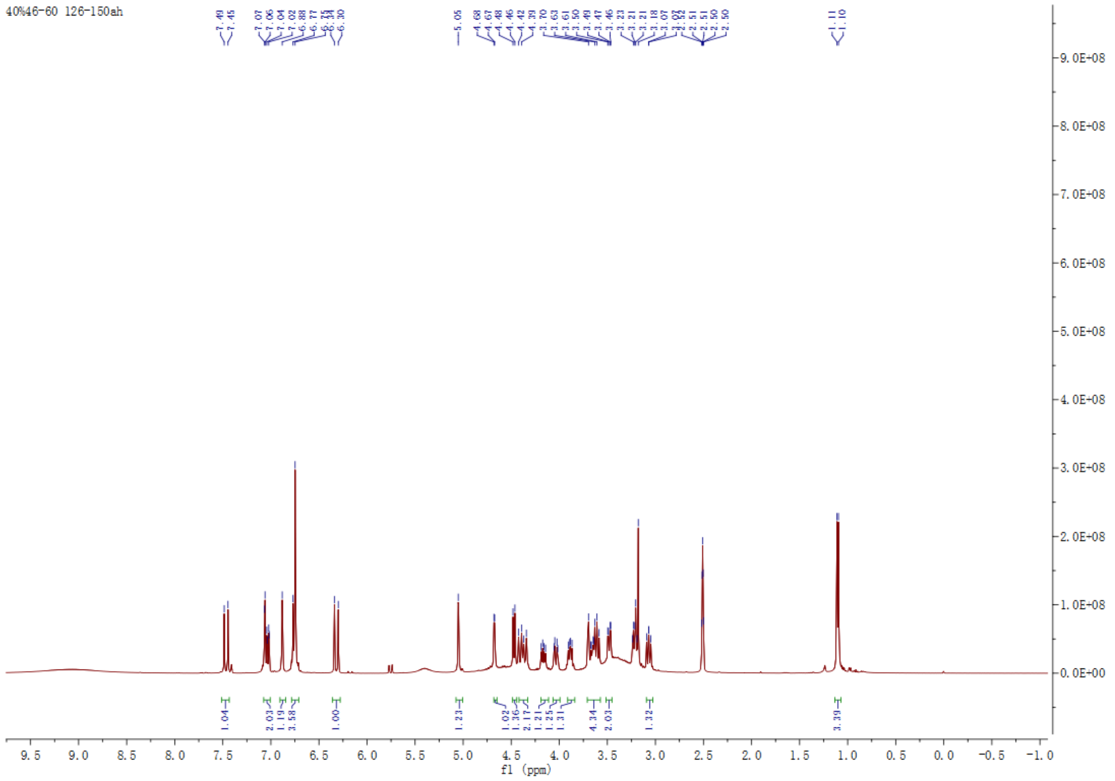

Figure S29. ^{1}H-NMR spectrum (500 MHz) of compound 9 in DMSO.

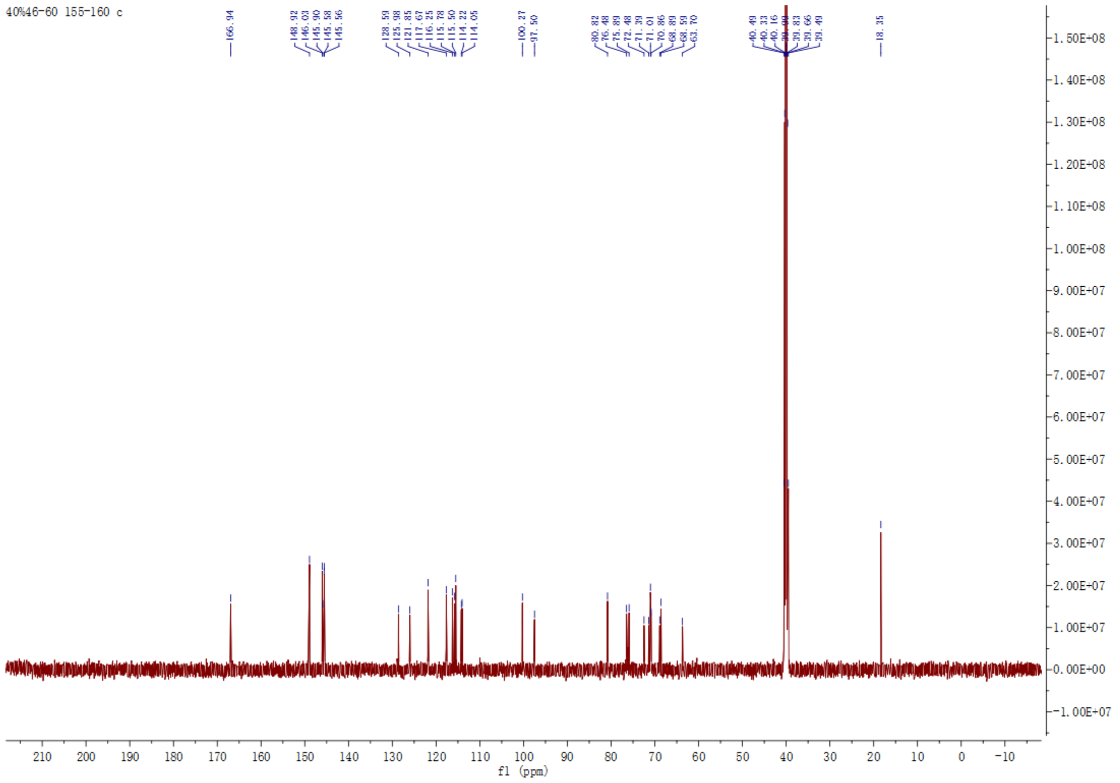

Figure S 30. ^{13}C-NMR spectrum (125 MHz) of compound 9 in DMSO.

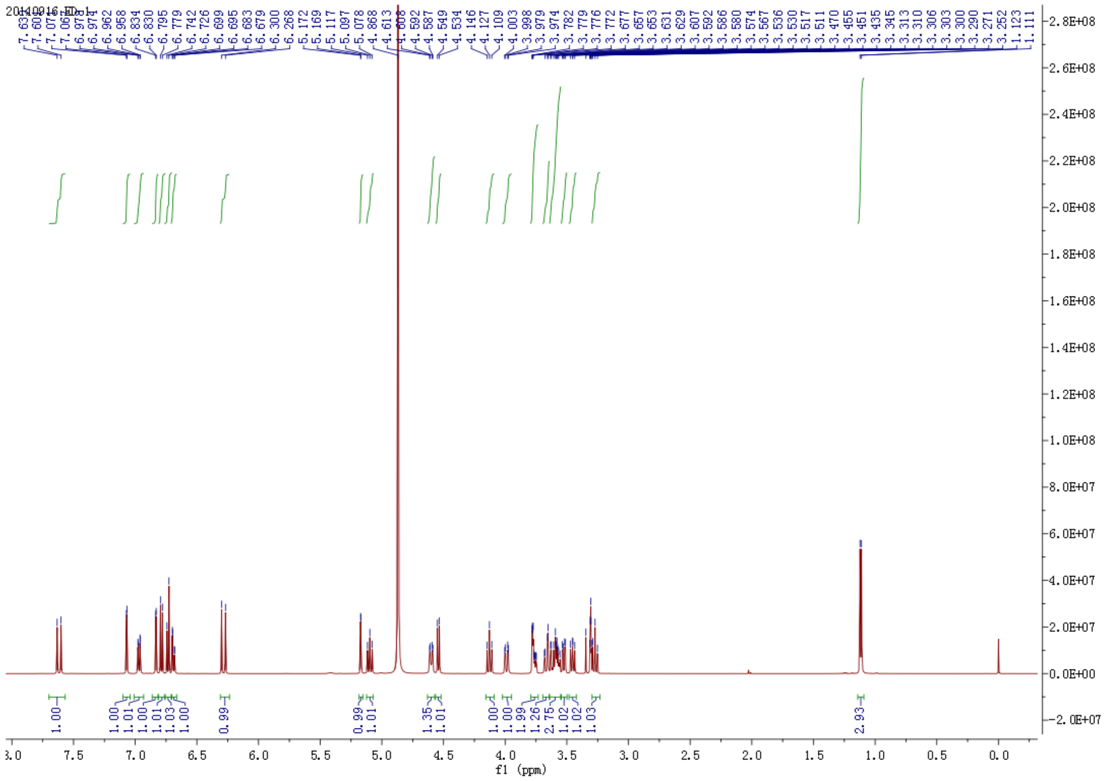

Figure S31. ^{1}H-NMR spectrum (500 MHz) of compound 10 in CD_3OD.

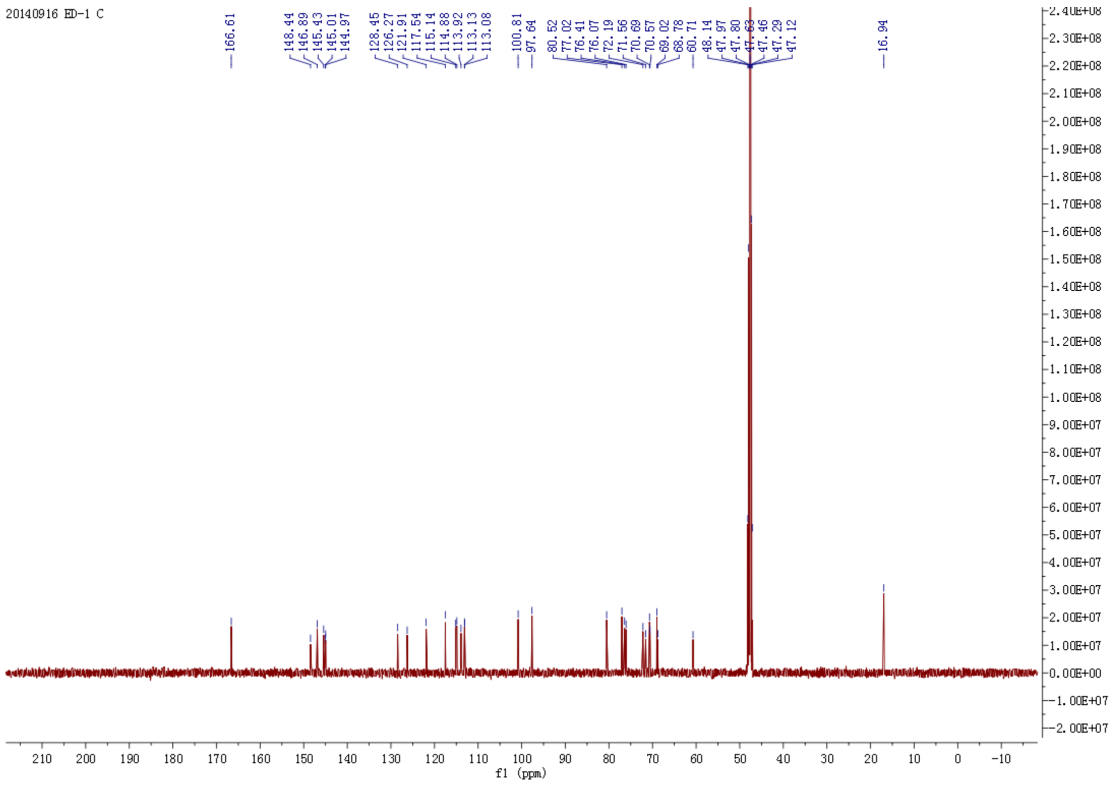

Figure S 32. ^{13}C-NMR spectrum (125 MHz) of compound 10 in CD_3OD.

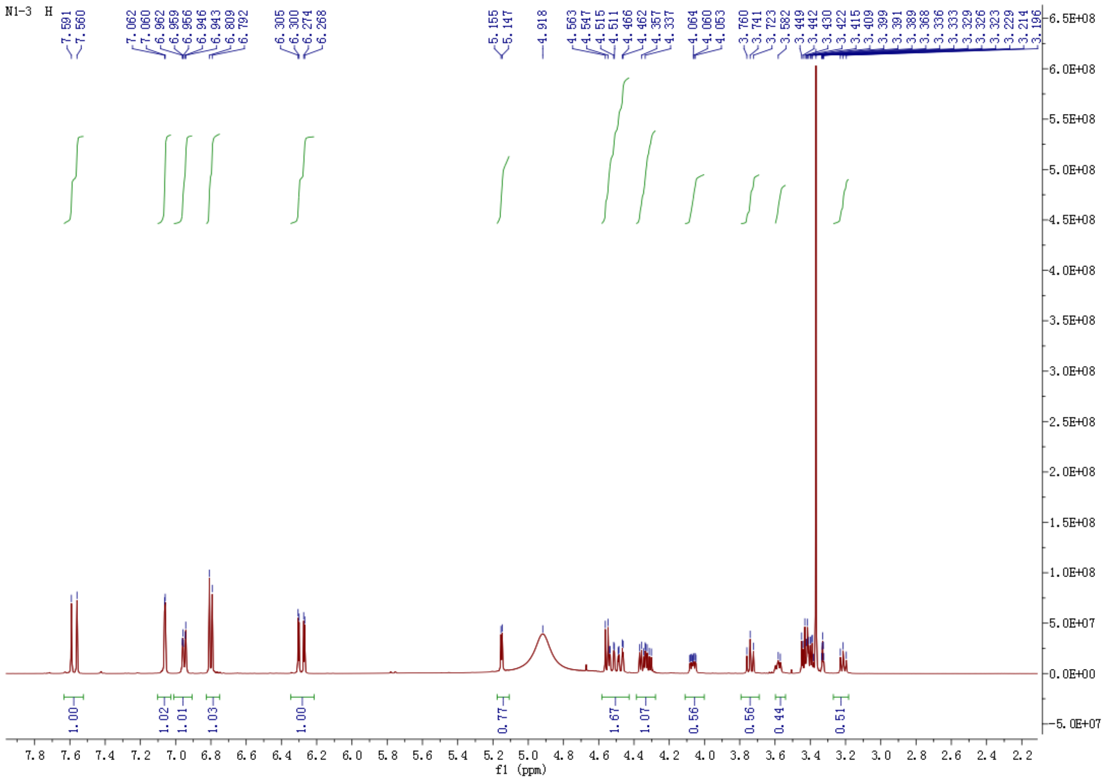

Figure S33. ^{1}H-NMR spectrum (500 MHz) of compound 11 in CD_3OD.

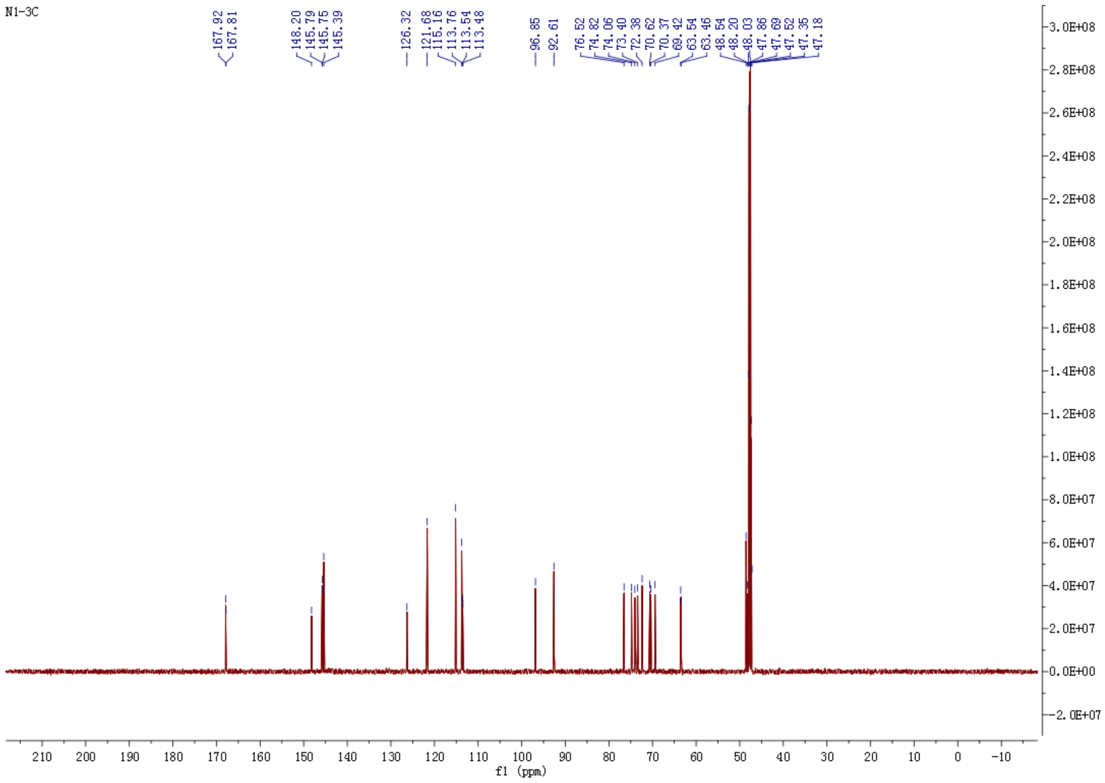

Figure S 34. ^{13}C-NMR spectrum (125 MHz) of compound 11 in CD_3OD.

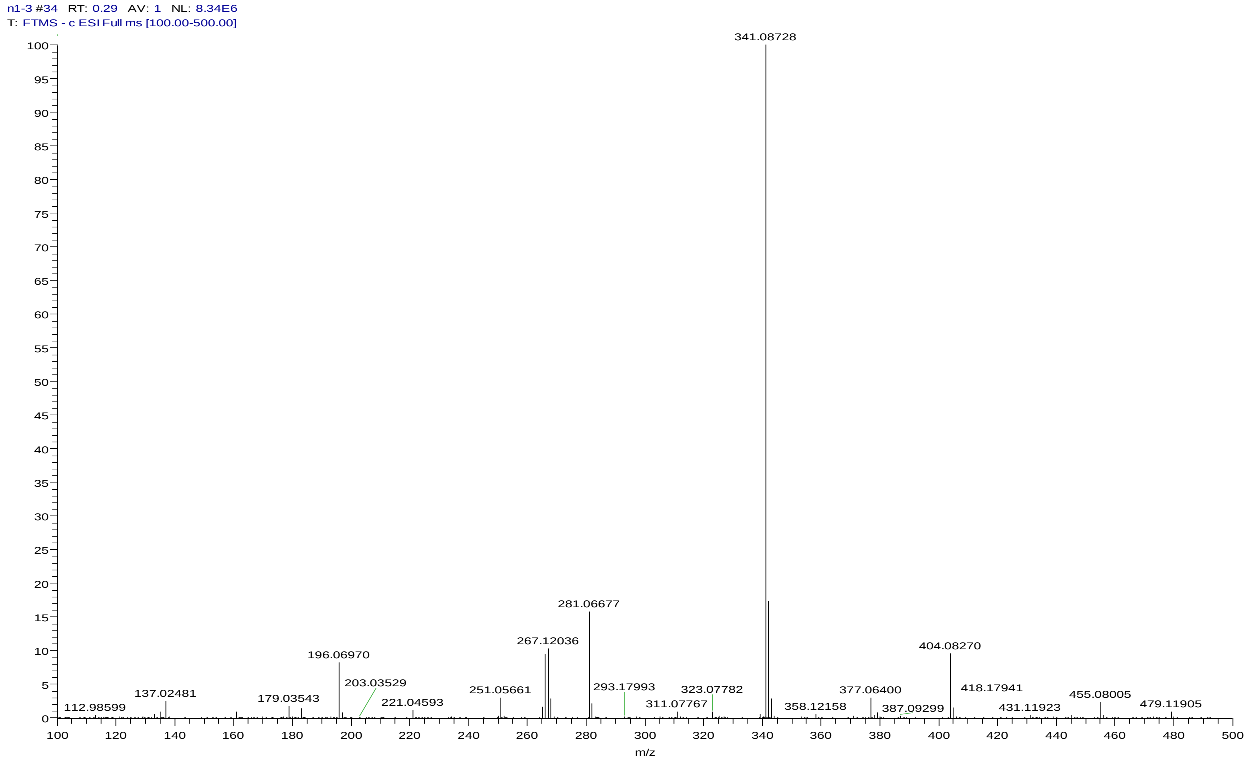

Figure S35. HR ESI (+) MS spectrum of compound 11.

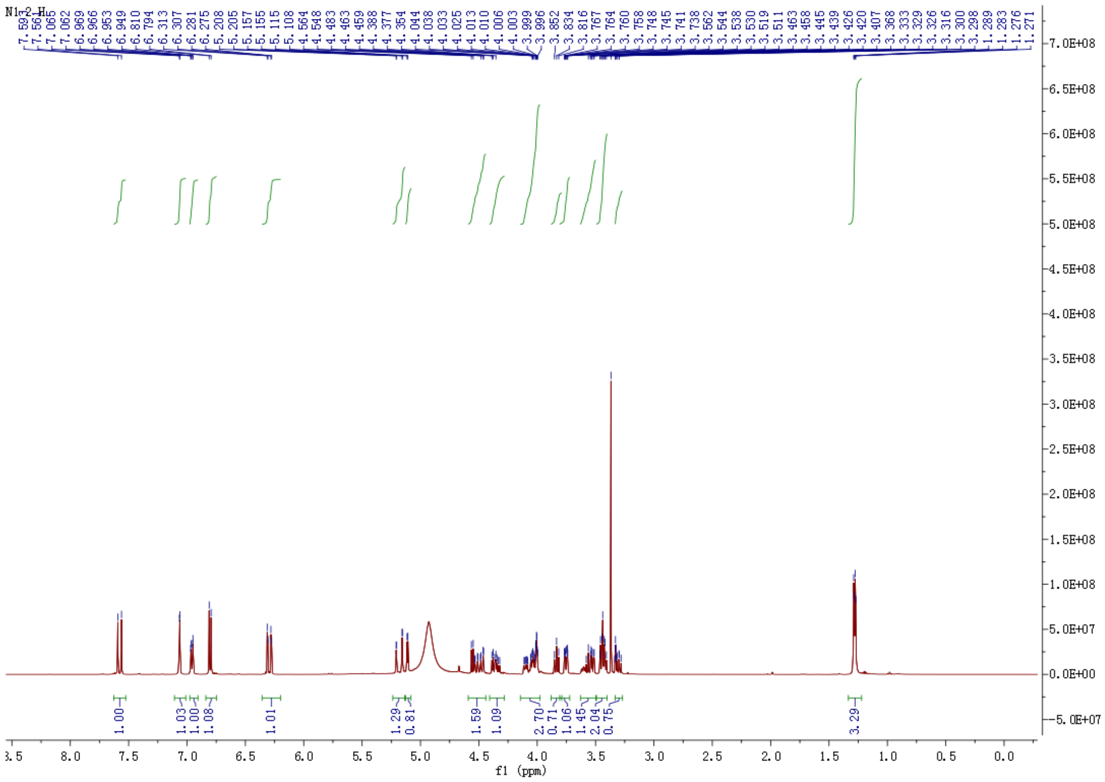

Figure S36. ^{1}H-NMR spectrum (500 MHz) of compound 12 in CD_3OD.

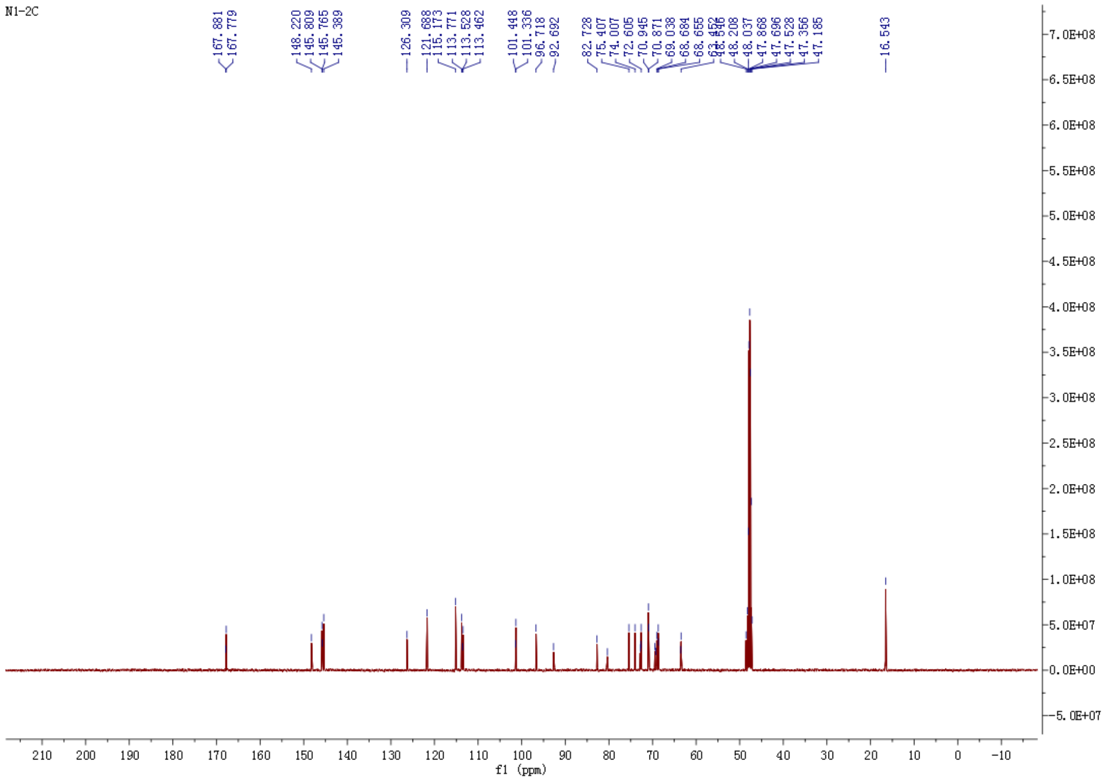

Figure S 37. ^{13}C-NMR spectrum (125 MHz) of compound 12 in CD_3OD.

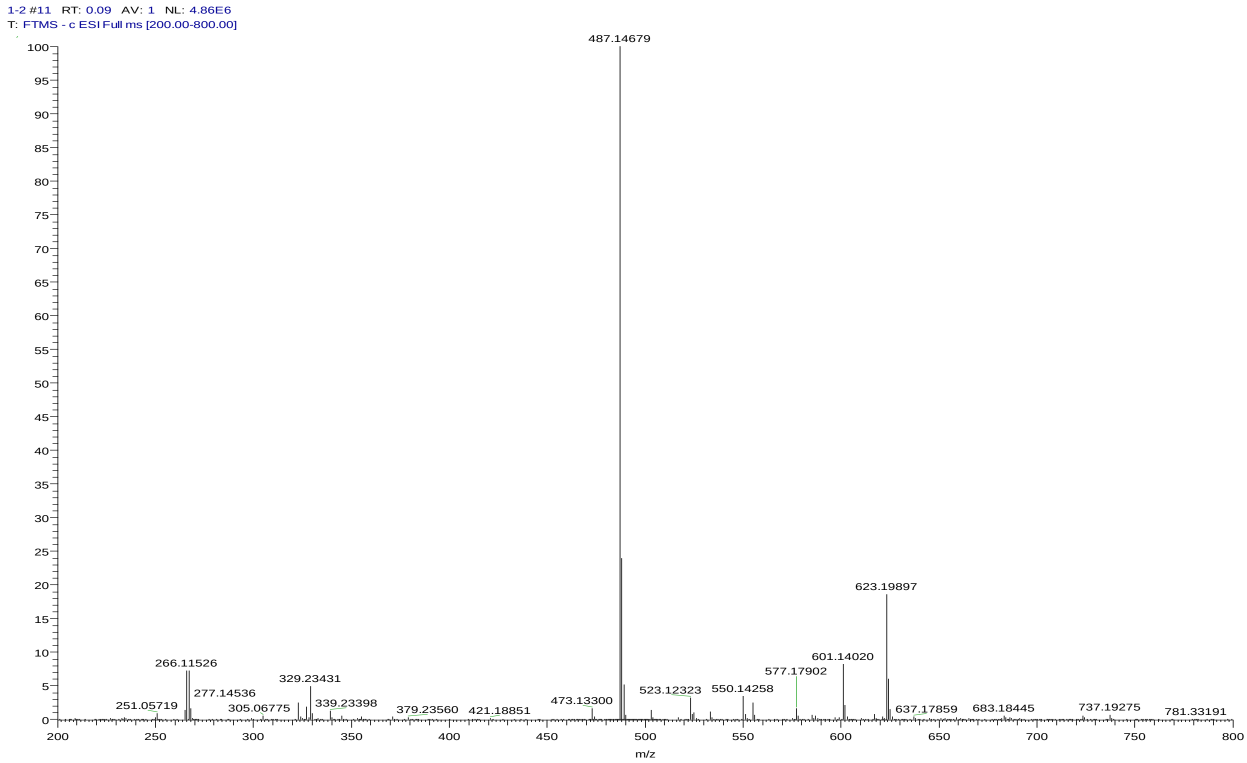

Figure S 38. HR ESI (+) MS spectrum of compound 12.

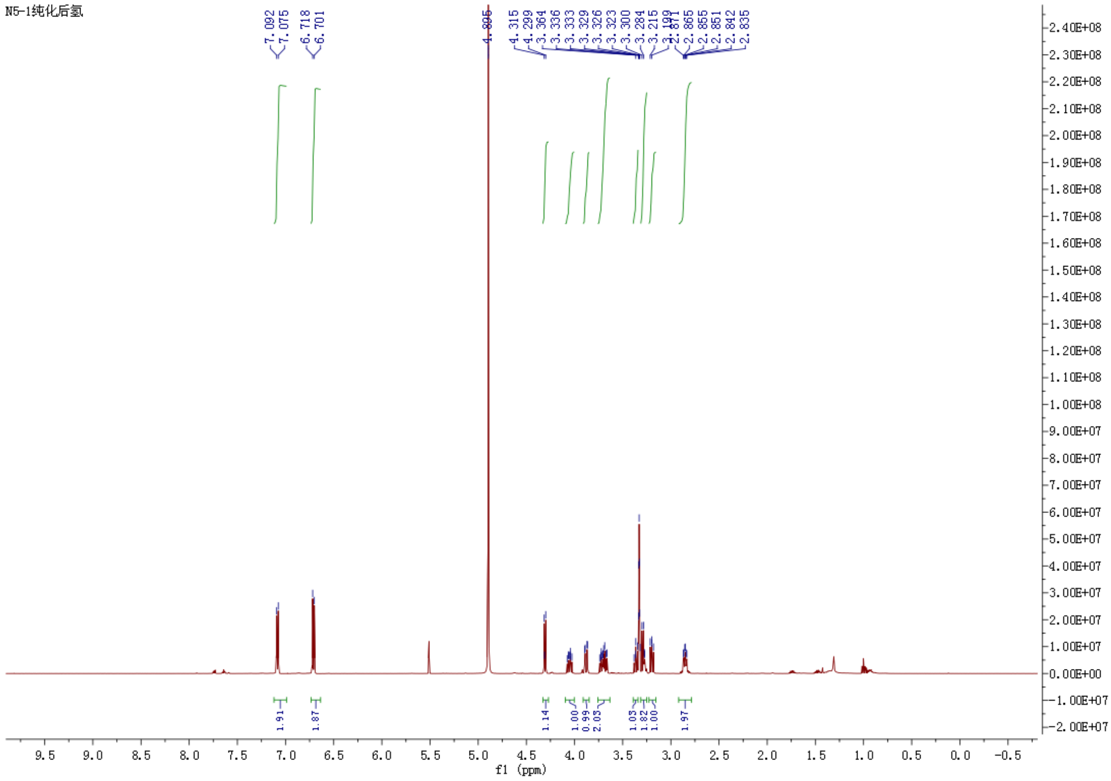

Figure S 39. ^{1}H-NMR spectrum (500 MHz) of compound 13 in CD_3OD.

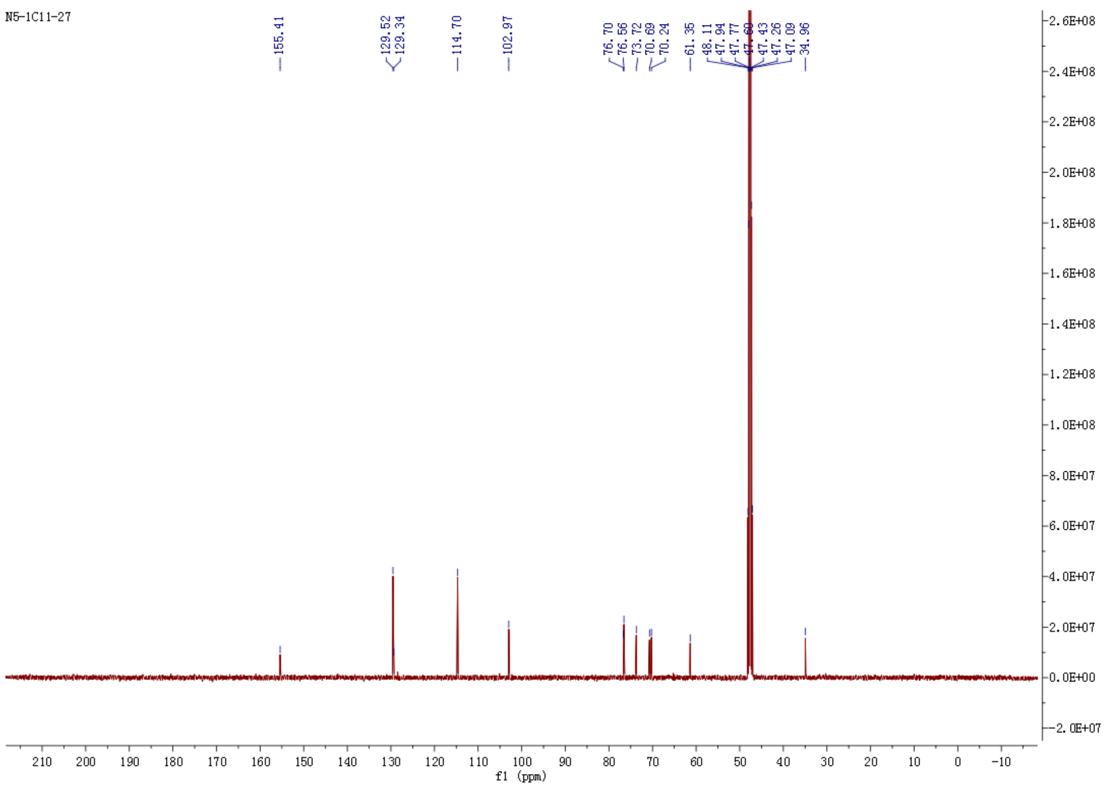

Figure S 40. ^{13}C-NMR spectrum (125 MHz) of compound 13 in CD_3OD.

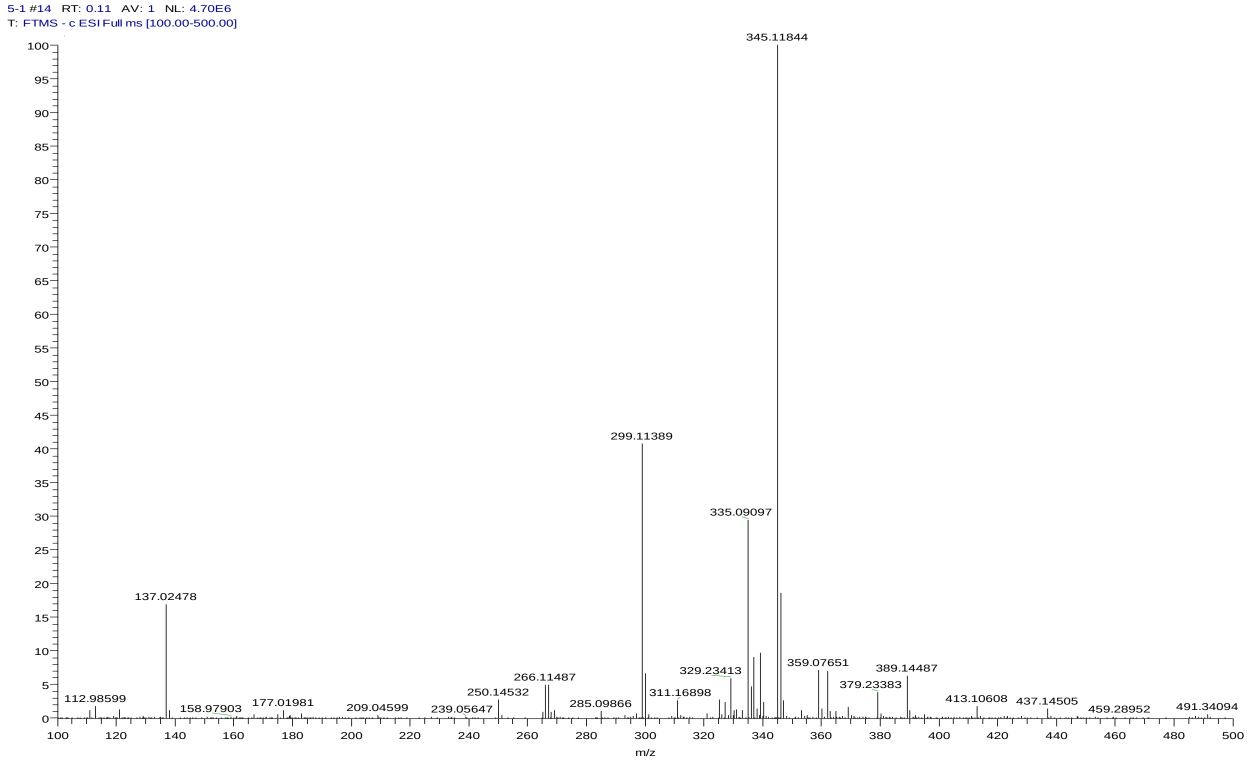

Figure S41. HR ESI (+) MS spectrum of compound 3.

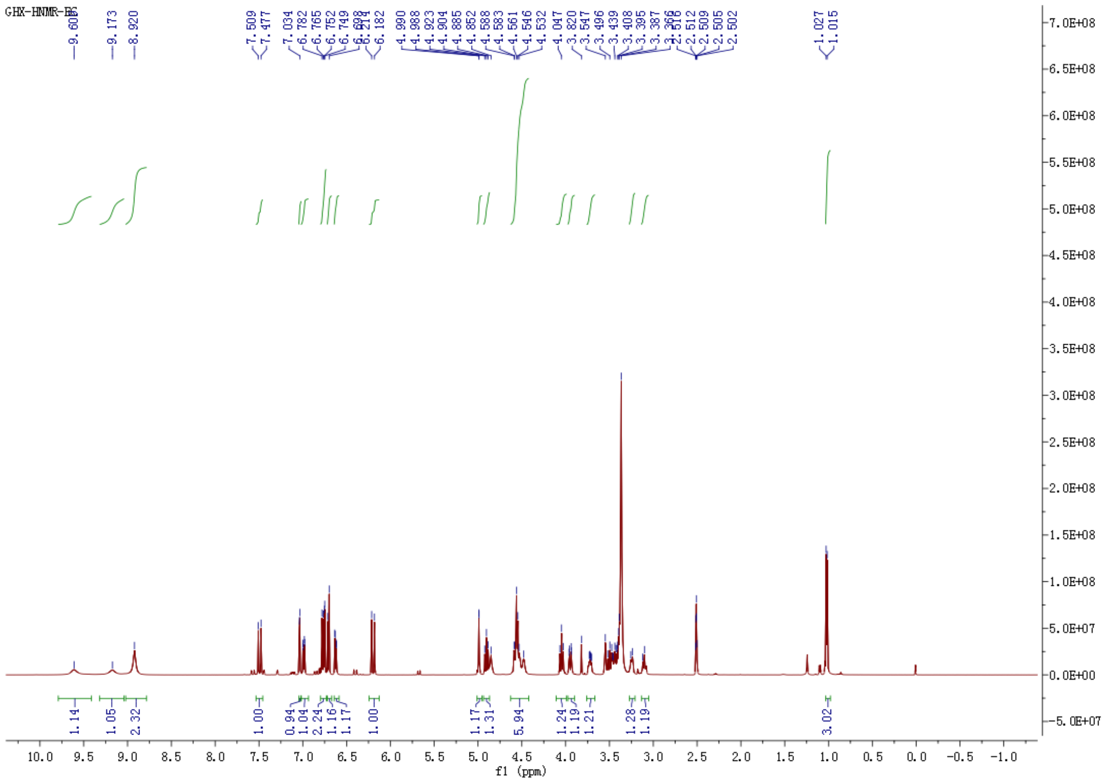

Figure S42. ¹H-NMR spectrum (500 MHz) of compound 14 in DMSO.

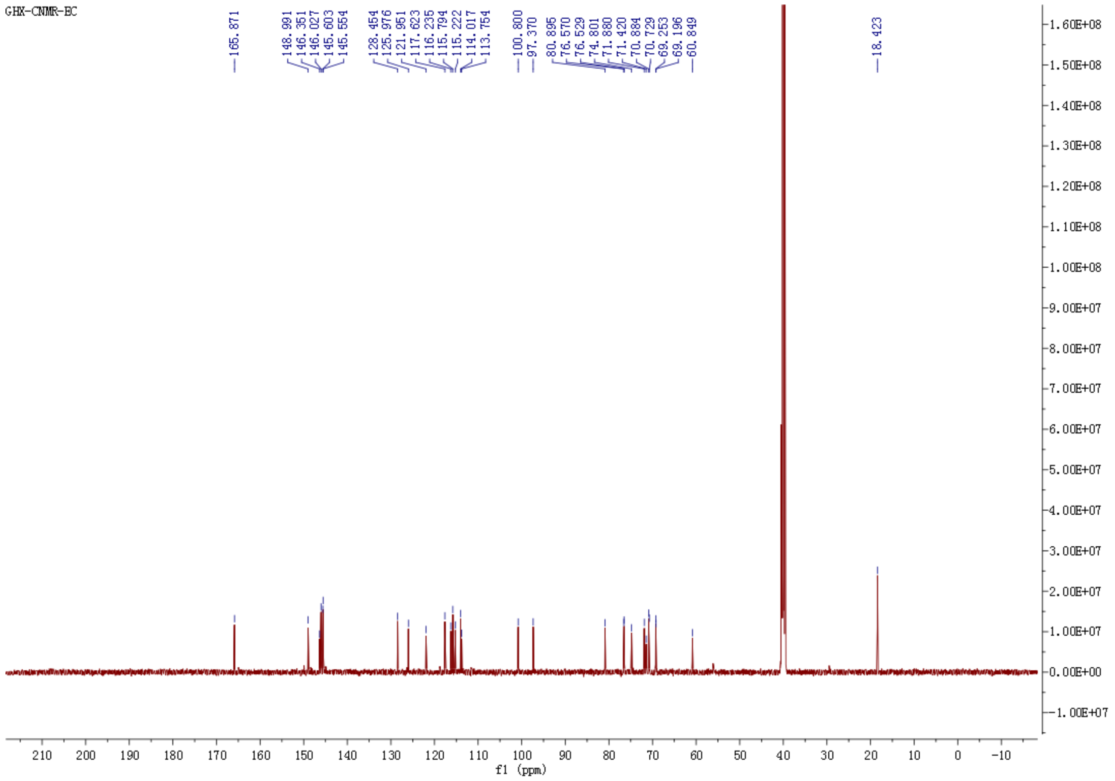

Figure S 43. ¹³C-NMR spectrum (125 MHz) of compound 14 in DMSO.

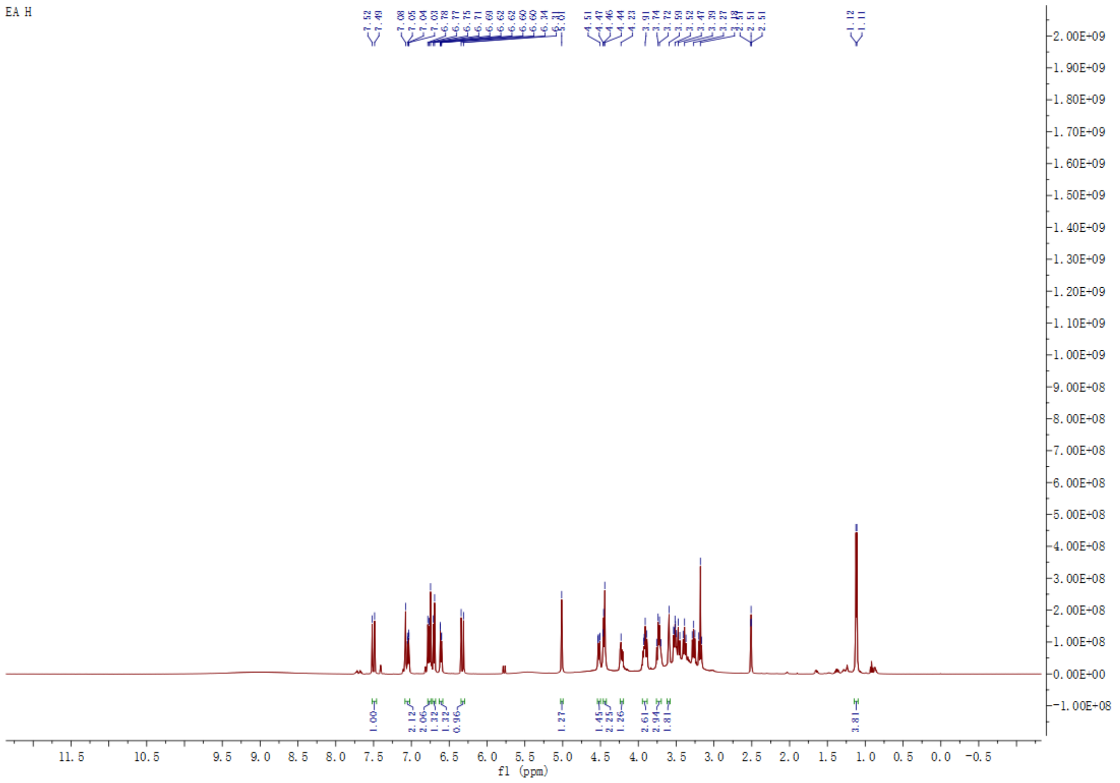

Figure S44. ^{1}H-NMR spectrum (500 MHz) of compound 15 in DMSO

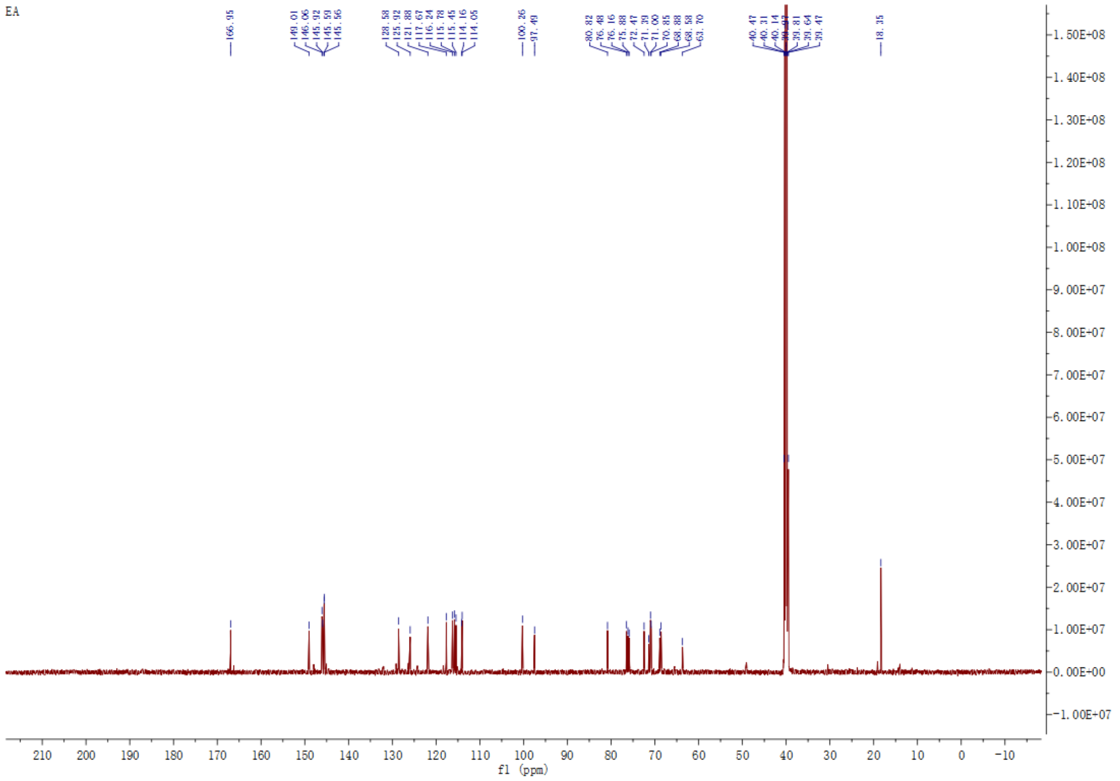

Figure S 45. ^{13}C-NMR spectrum (125 MHz) of compound 15 in DMSO.

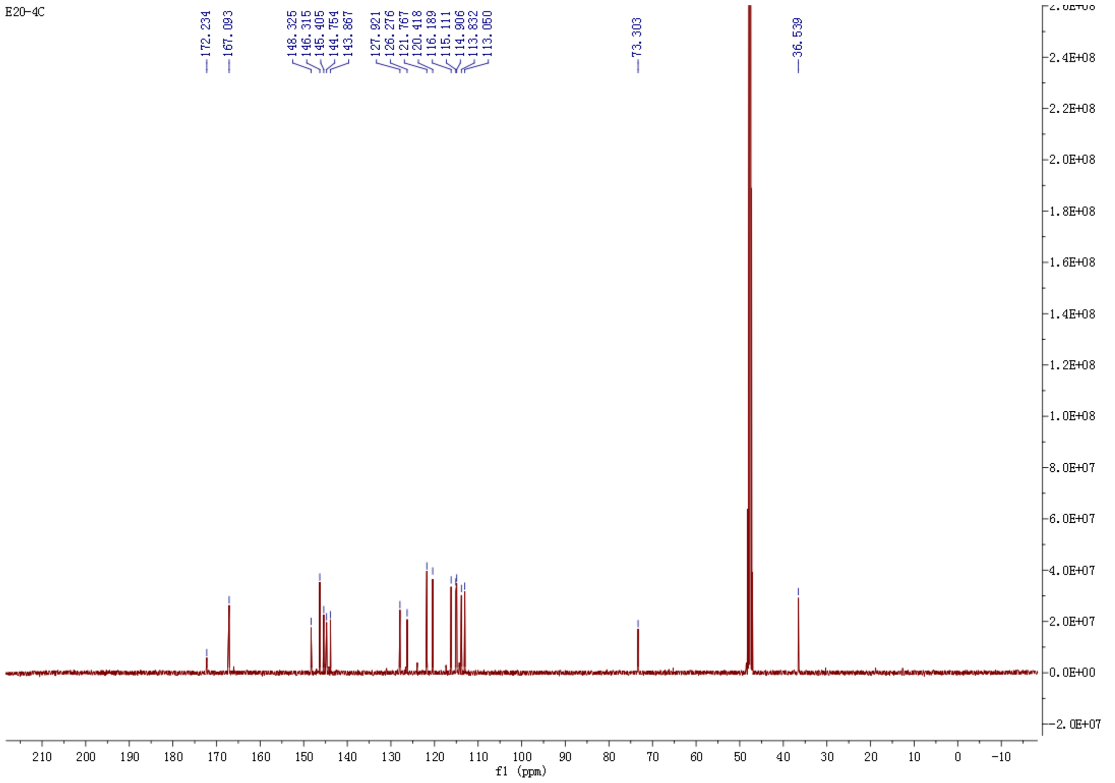

Figure S46. ^{1}H-NMR spectrum (500 MHz) of compound 16 in CD_3OD.

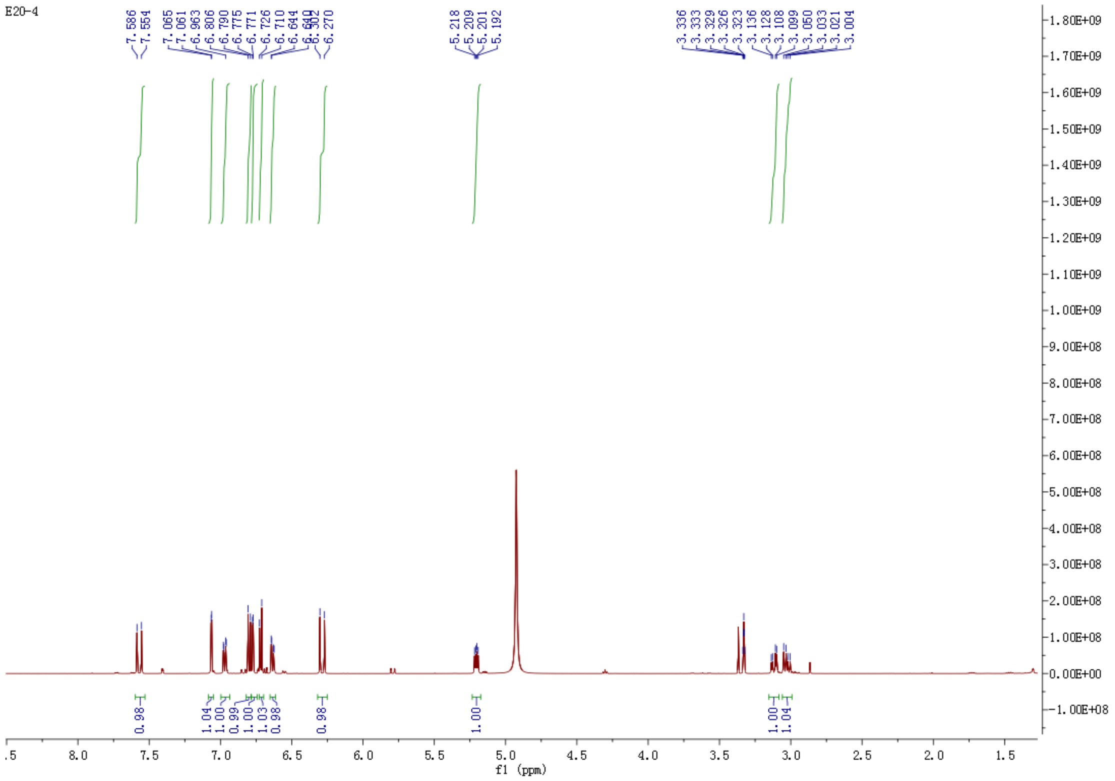

Figure S 47. ^{13}C-NMR spectrum (125 MHz) of compound 16 in CD_3OD.

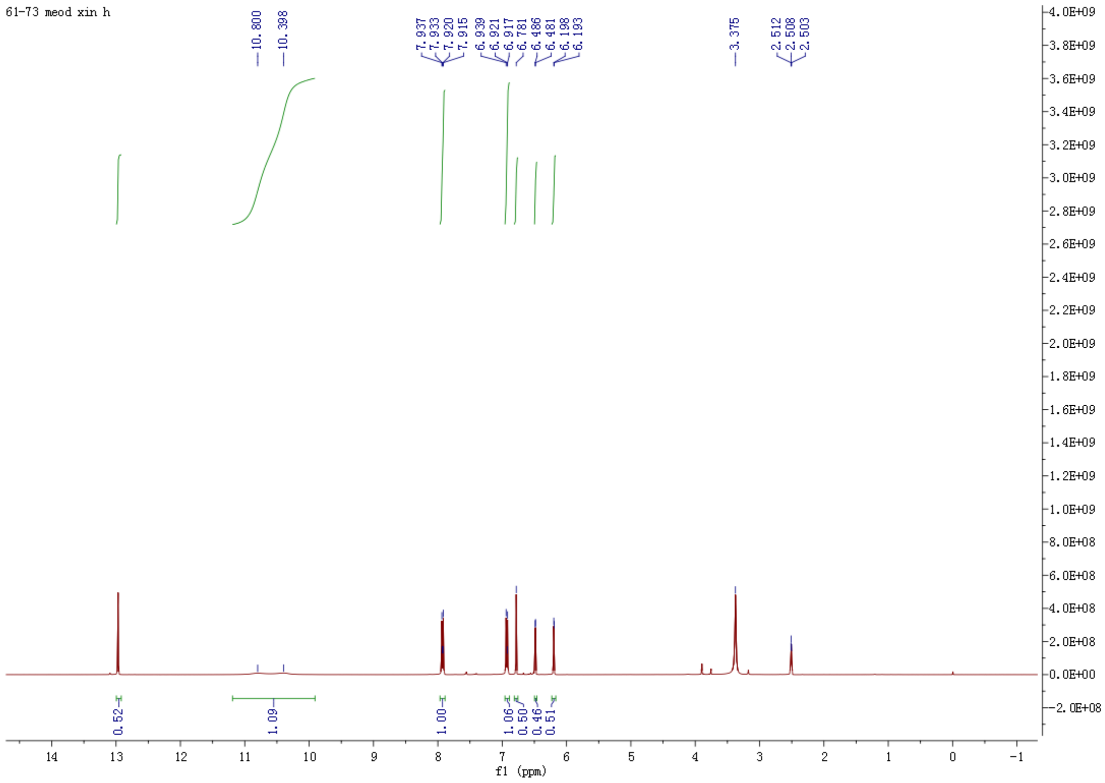

Figure S48. ^{1}H-NMR spectrum (500 MHz) of compound 17 in DMSO.

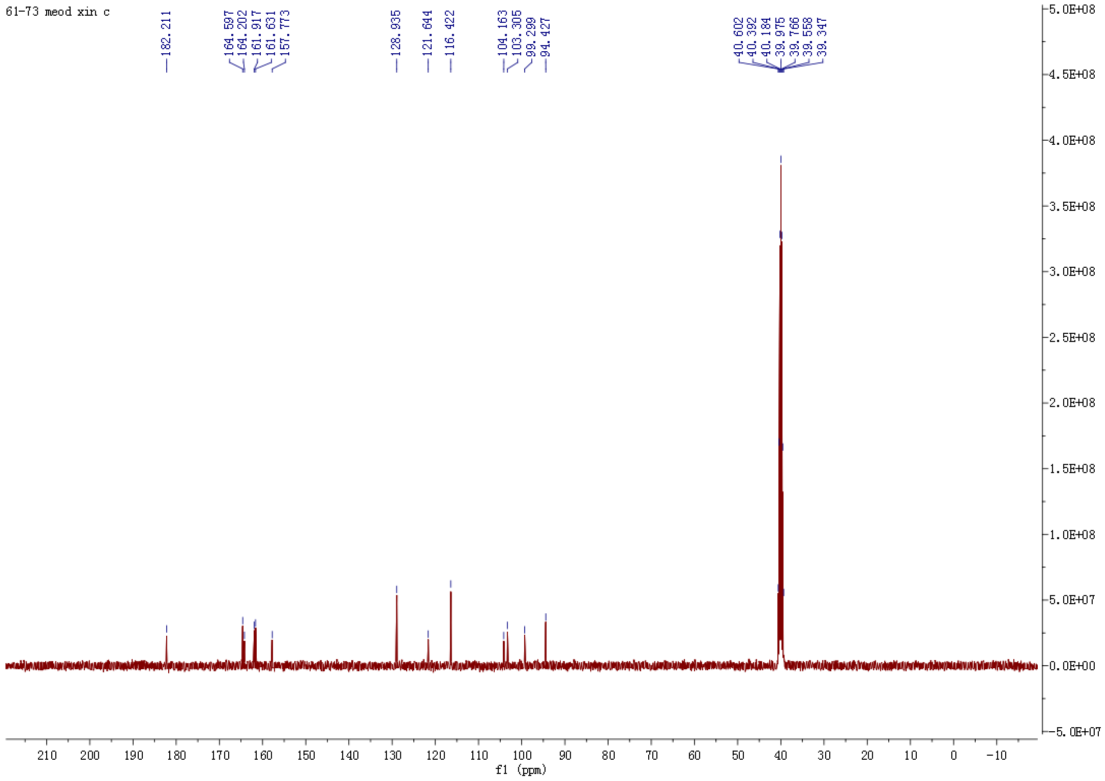

Figure S 49. ^{13}C-NMR spectrum (125 MHz) of compound 17 in DMSO.

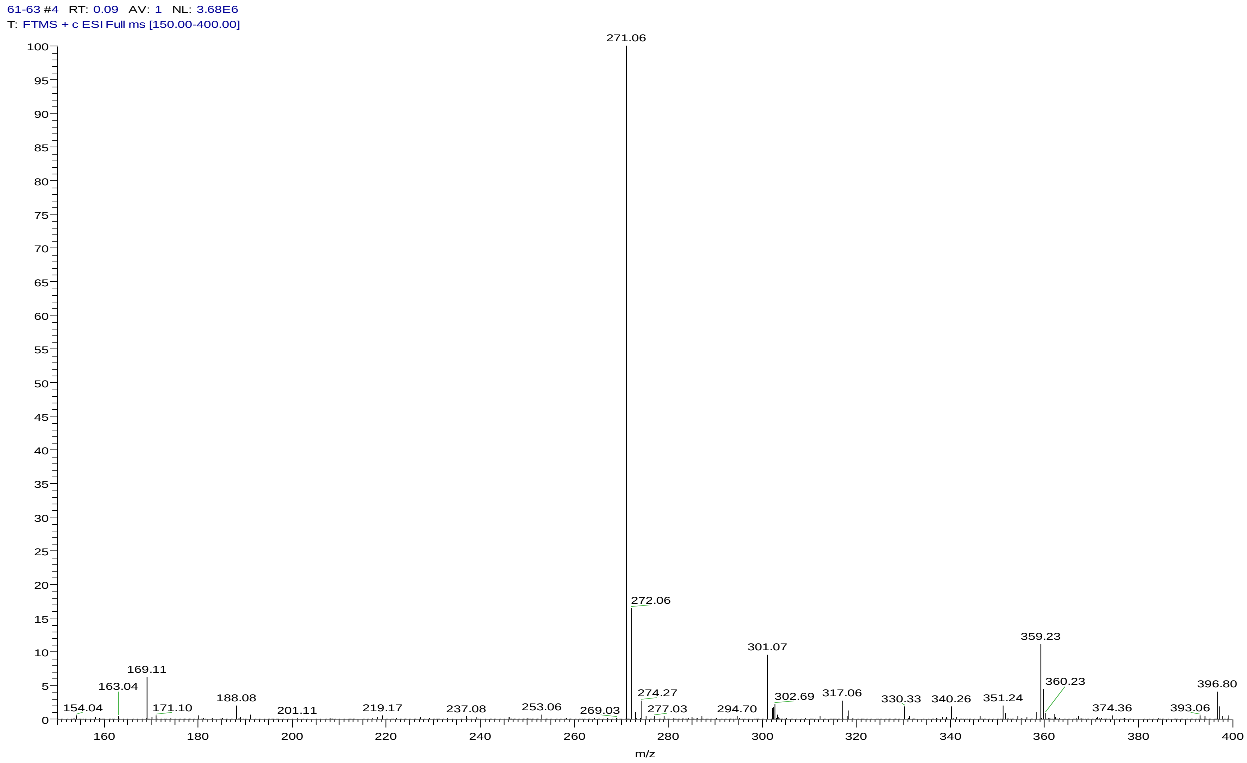

Figure S50. HR ESI (+) MS spectrum of compound 17.

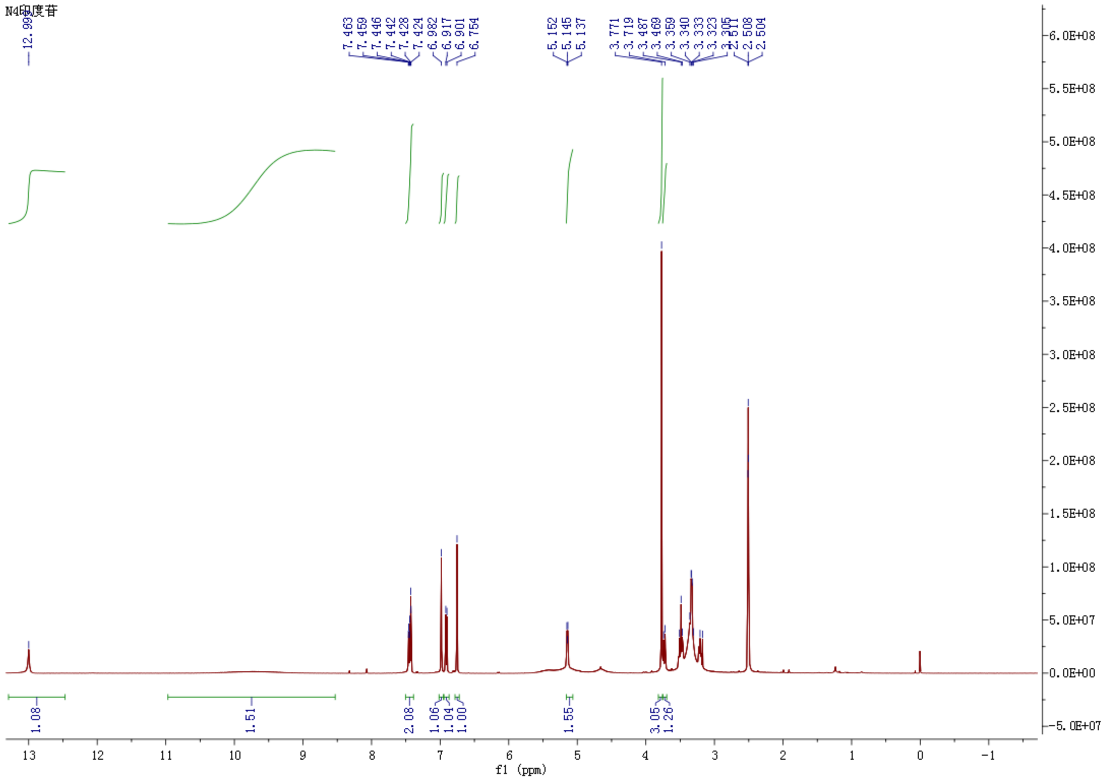

Figure S51. ^{1}H-NMR spectrum (500 MHz) of compound 18 in DMSO.

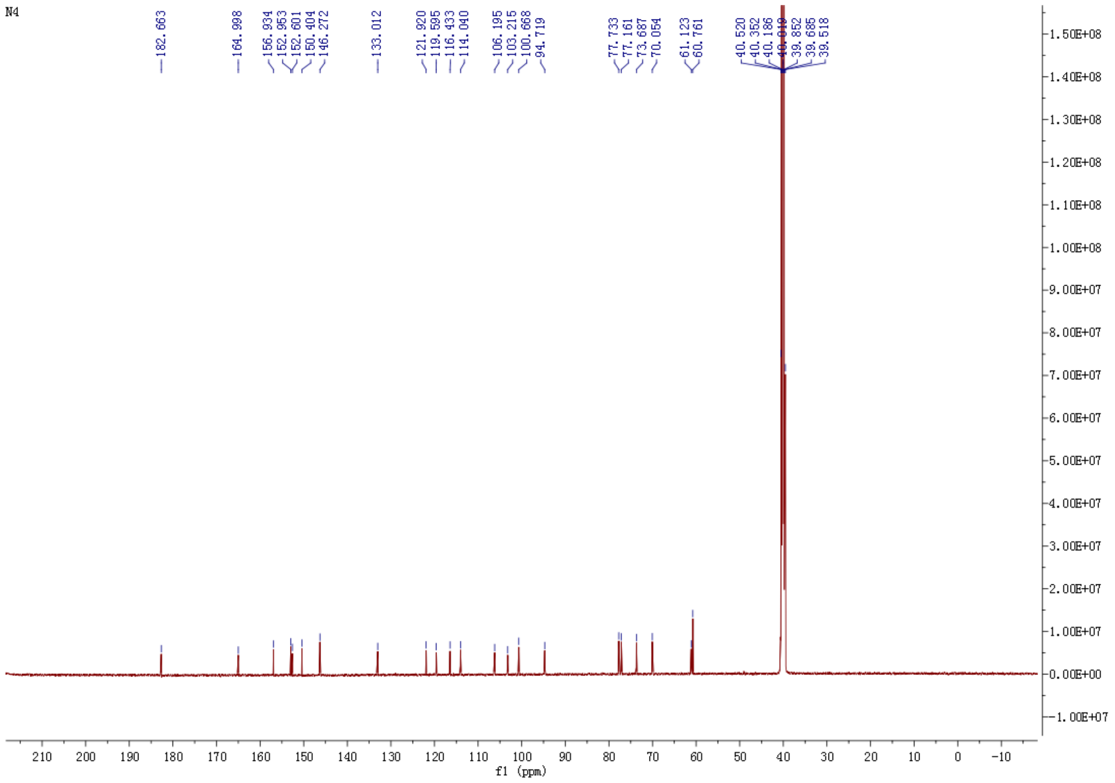

Figure S 52. ^{13}C-NMR spectrum (125 MHz) of compound 18 in DMSO.

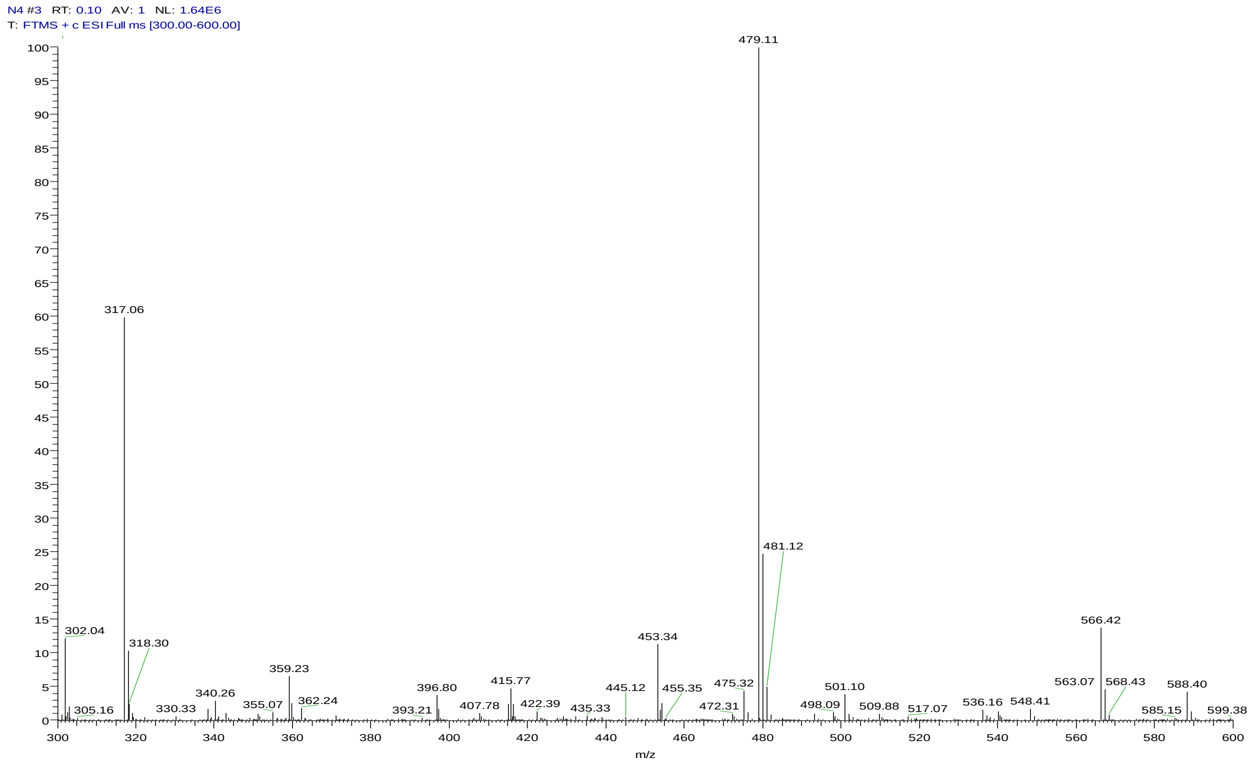

Figure S53. HR ESI (+) MS spectrum of compound 18.

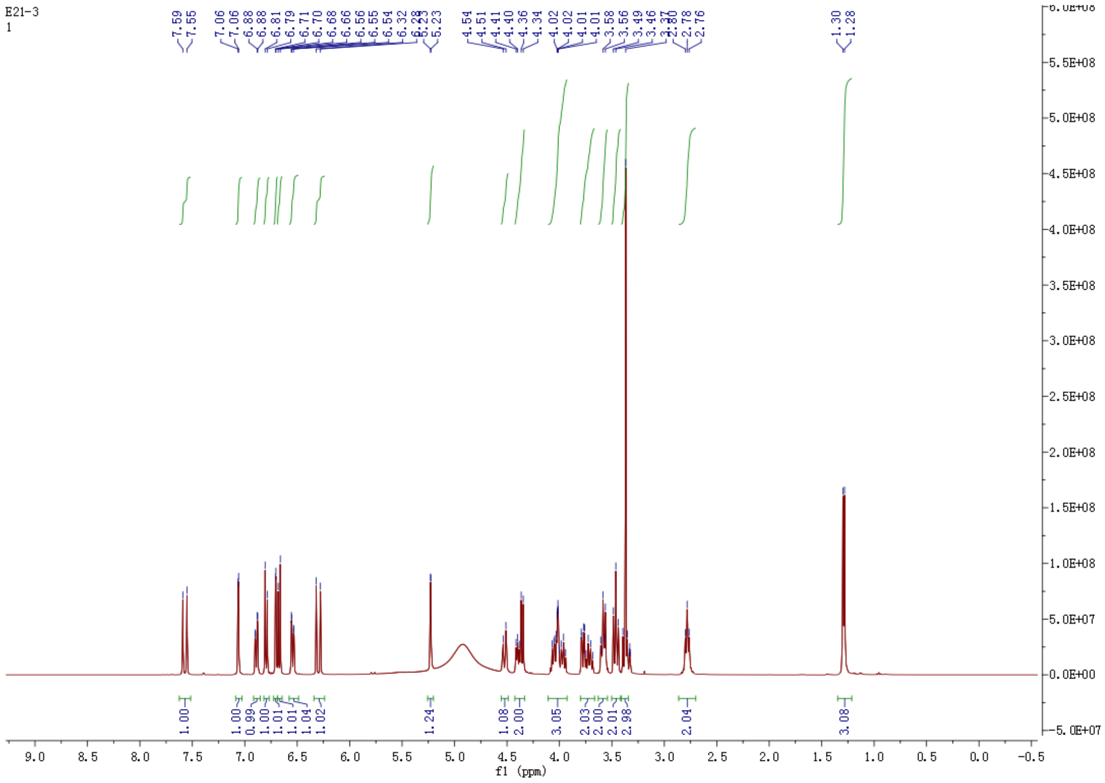

Figure S54. ^{1}H-NMR spectrum (500 MHz) of compound 19 in CD_3OD.

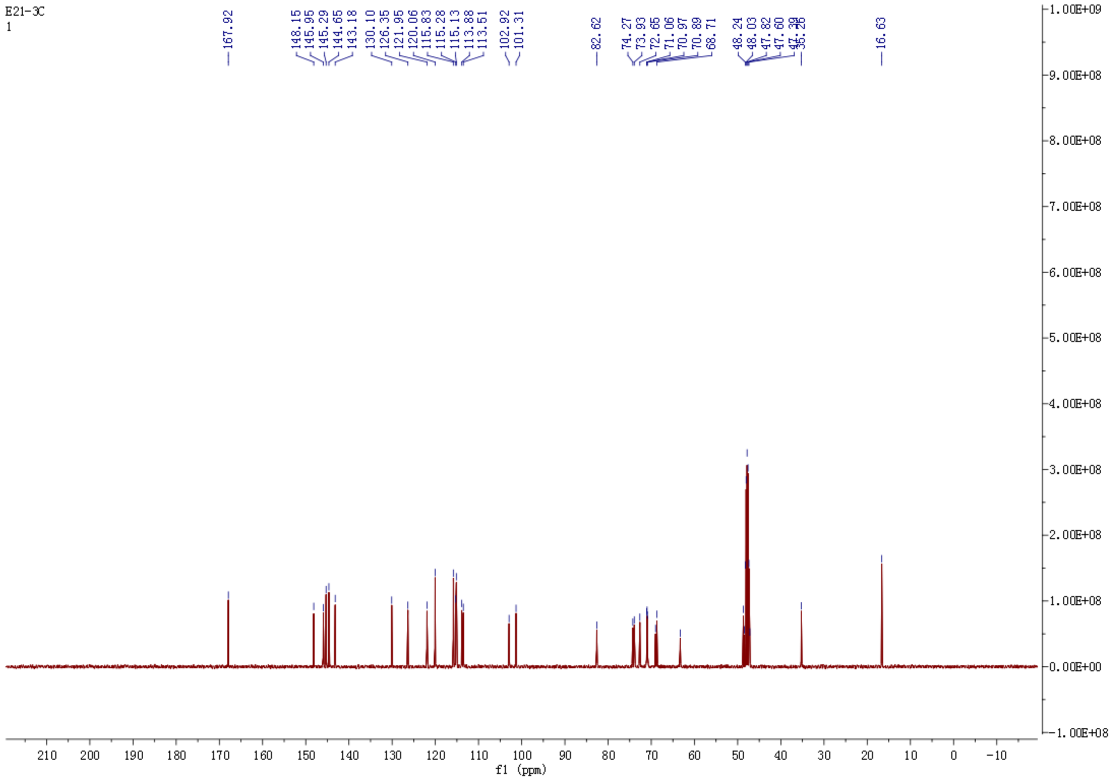

Figure S 55. ^{13}C-NMR spectrum (125 MHz) of compound 19 in CD_3OD.

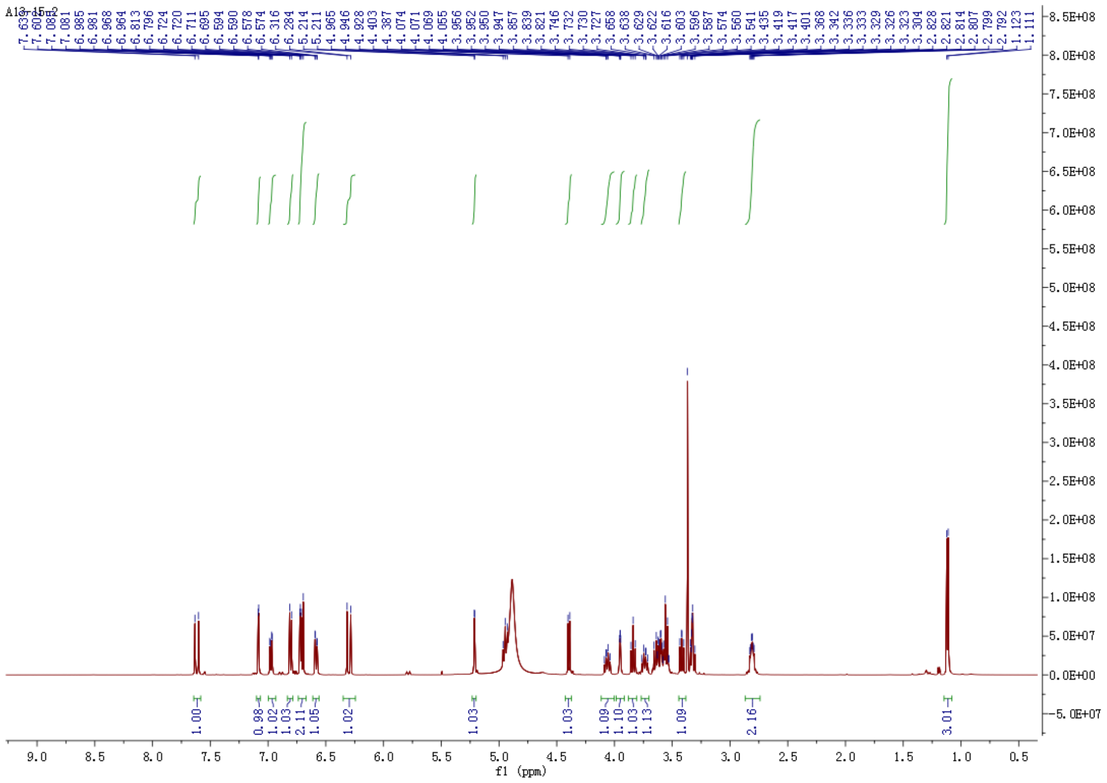

Figure S56. ^{1}H-NMR spectrum (500 MHz) of compound 20 in CD_3OD.

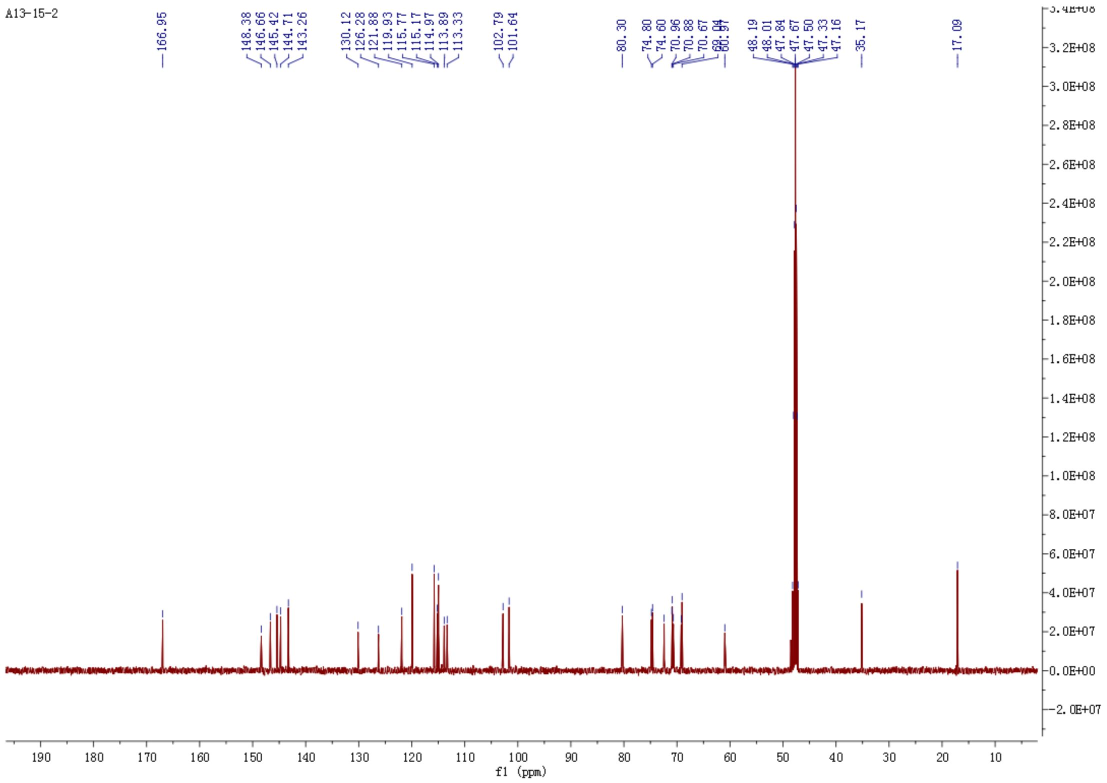

Figure S 57. ^{13}C-NMR spectrum (125 MHz) of compound 20 in CD_3OD.

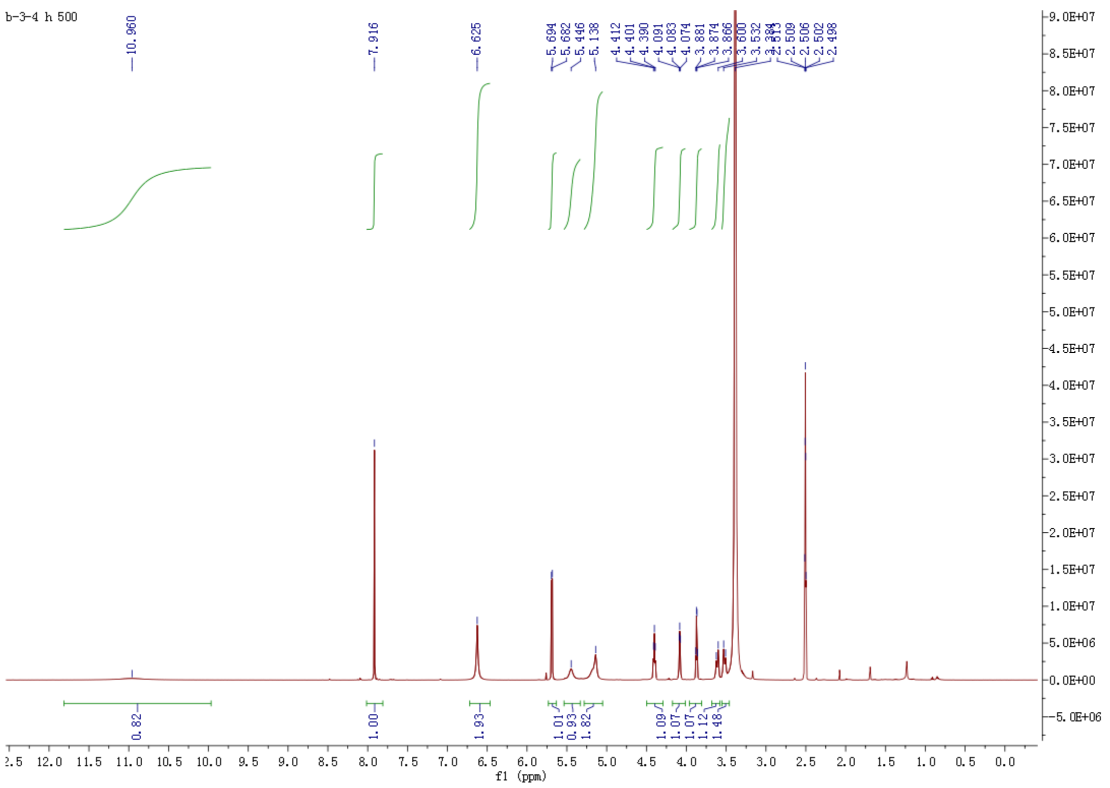

Figure S58. ^{1}H-NMR spectrum (500 MHz) of compound 21 in DMSO.

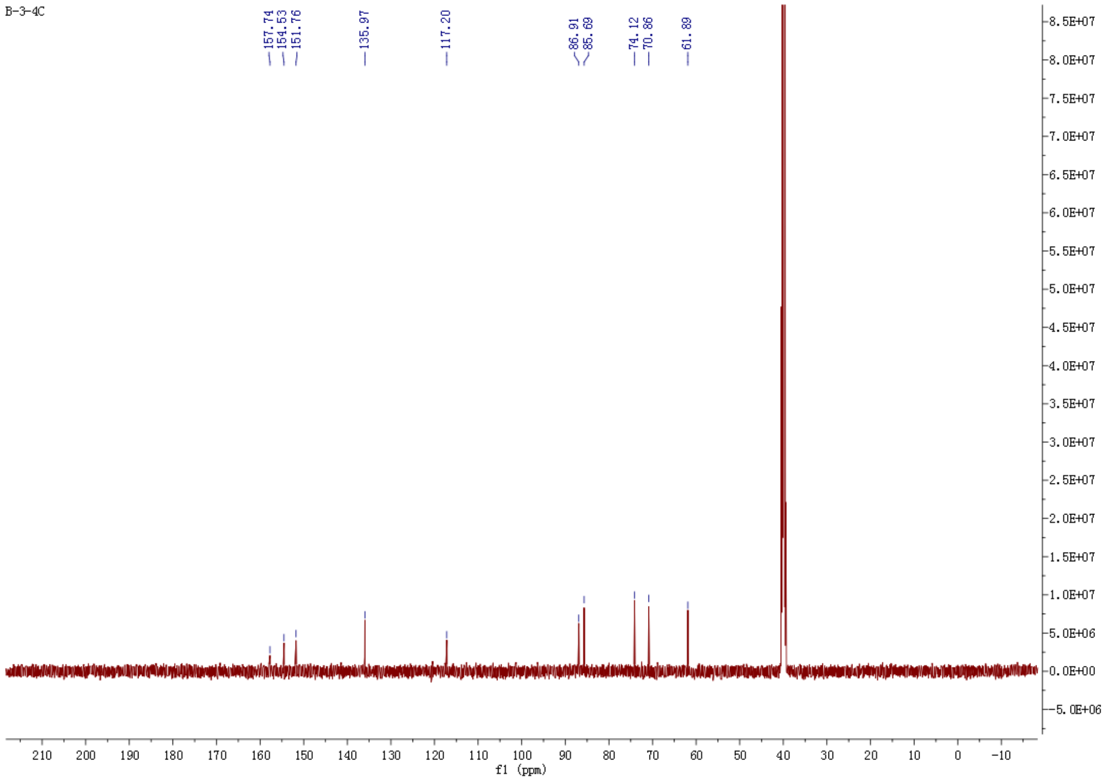

Figure S 59. ^{13}C-NMR spectrum (125 MHz) of compound 21 in DMSO.

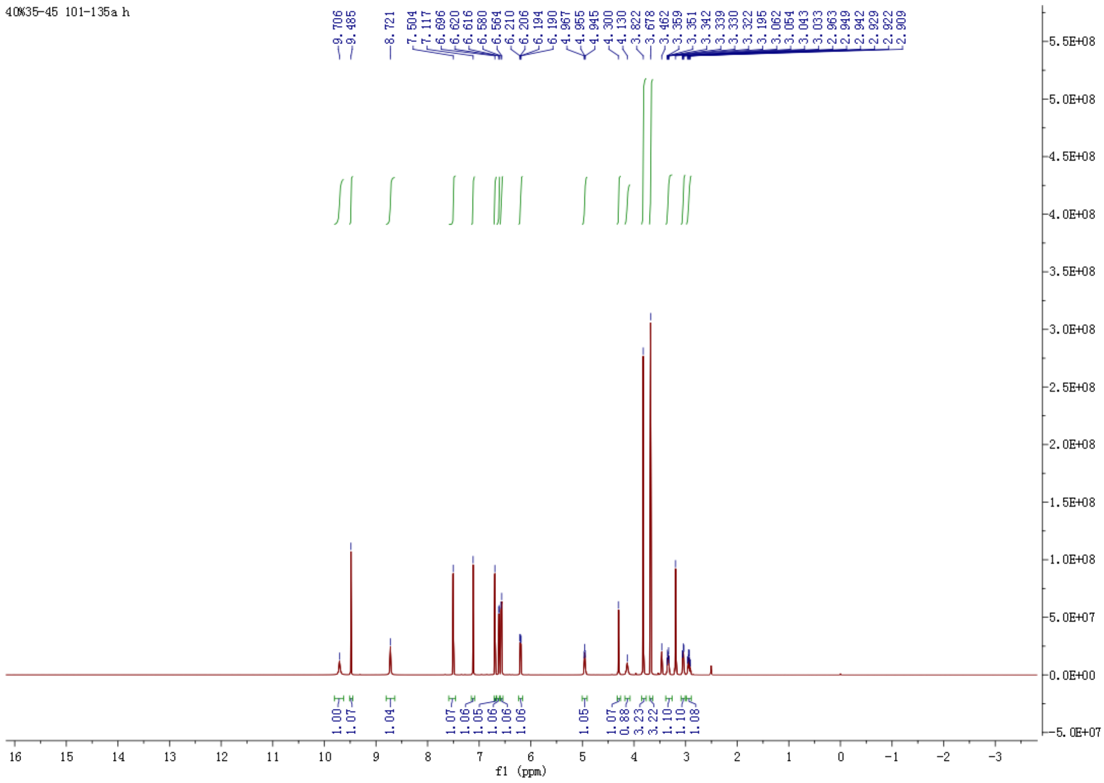

Figure S60. ^{1}H-NMR spectrum (500 MHz) of compound 22 in DMSO.

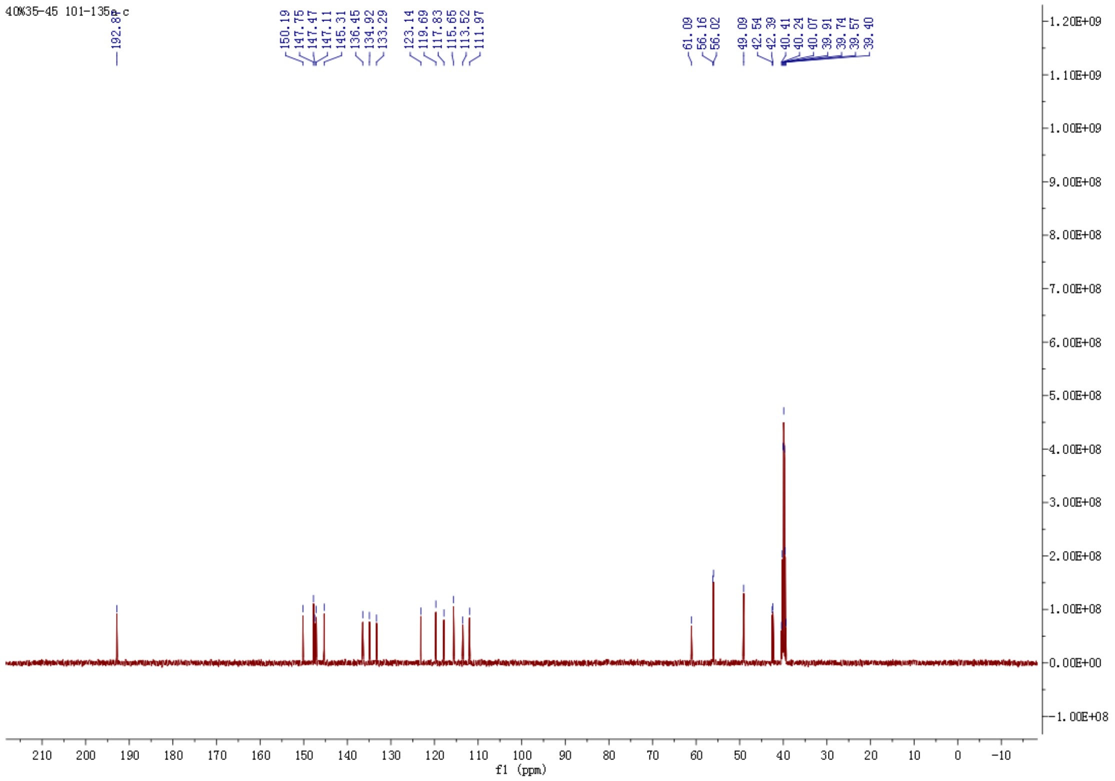

Figure S 61. ^{13}C-NMR spectrum (125 MHz) of compound 22 in DMSO.

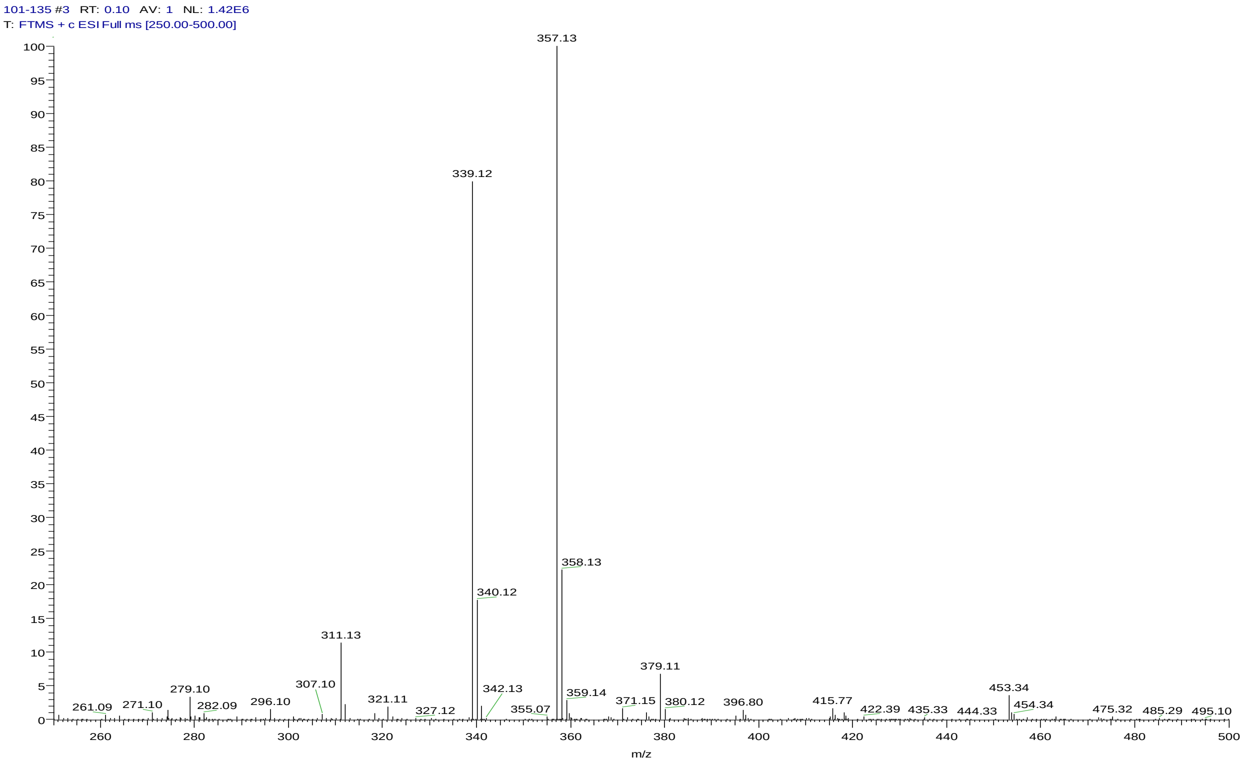

Figure S62. HR ESI (+) MS spectrum of compound 22.
